# Donor−acceptor catalysis with defective MoS_2_ and Fe^0^ breaks barriers to perchlorate reduction under mild conditions

**DOI:** 10.1038/s41467-026-73219-5

**Published:** 2026-05-20

**Authors:** Hejie Qin, Han Yang, Zhenmin Zhang, Yiran Feng, Yuankui Sun, Peng Fan, Zhimin Ao, T. David Waite, Xiaohong Guan

**Affiliations:** 1 Shanghai Engineering Research Center of Biotransformation of Organic Solid Waste, School of Ecological and Environmental Sciences, East China Normal University, Shanghai, P. R. China; 2 Shanghai Synchrotron Radiation Facility, Shanghai Advanced Research Institute, Chinese Academy of Sciences, Shanghai, P. R. China; 3 Advanced Interdisciplinary Institute of Environment and Ecology, Guangdong Provincial Key Laboratory of Wastewater Information Analysis and Early Warning, Beijing Normal University, Zhuhai, P. R. China; 4 Water Research Centre, School of Civil and Environmental Engineering, University of New South Wales, Sydney, NSW Australia; 5 Institute of Eco-Chongming, Shanghai, P. R. China

**Keywords:** Heterogeneous catalysis, Catalytic mechanisms, Pollution remediation

## Abstract

Perchlorate (ClO_4_^−^) contamination in water poses global health risks, yet its efficient reduction to harmless Cl^−^ under mild conditions remains challenging. Here, we report a donor−acceptor catalytic system comprising defective MoS_2_ on N-doped carbon (MoS_2_−NC) coupled with zerovalent iron (Fe^0^), which enables rapid ClO_4_^−^ reduction at near-neutral pH (rate constant, 2.36 h^−1^), yielding Cl^−^ as the sole product. In the MoS_2_−NC/Fe^0^ system, Fe^0^ acts as the electron donor, while undercoordinated Mo atoms in defective MoS_2_ serve as the active sites, and N-doped carbon mediates electron transfer and optimizes the electronic environment for ClO_4_^−^ reduction. The reduction proceeds via oxygen atom transfer, involving Cl−O bond cleavage, O binding to Mo sites, and hydrodeoxygenation of the Mo-bound O atoms. Our observations offer a practical strategy for ClO_4_^−^ reduction without harsh conditions or noble metals and underscore the promise of donor−acceptor-based, defect-engineered catalysts for reductive transformation of challenging oxyanions.

## Abstract (web summary)

Removing perchlorate from water is difficult without harsh conditions or precious metals. A defective MoS₂ catalyst paired with iron rapidly converts perchlorate to chloride near neutral pH by channeling electrons to active molybdenum sites.

## Introduction

Perchlorate (ClO_4_^−^) has emerged as a widespread contaminant in groundwater, surface water, and soil, due to its extensive release as a result of its use in the aerospace sector, military, fireworks, and other industries^1^. This highly mobile and toxic compound can impede human growth, metabolism, and development, posing threats to human health and also environmental safety^2,3^. Therefore, the World Health Organization (WHO) and many countries have set maximum contamination levels for perchlorate in drinking water, ranging from 2 to 70 μg/L^4,5^. Interestingly, perchlorate is also abundant in both Martian and lunar soils, with its presence related to photochemical reactions involving chlorine and ozone^6^. Due to both concerns regarding its adverse effects on human health and its prevalence in Martian and lunar soils, the elimination of ClO_4_^−^ contamination has recently attracted significant attention in environmental engineering and space exploration.

Although physical separation methods (e.g., ion exchange and membrane filtration) can effectively remove ClO_4_^−^ from water^3,7^, they do not reduce it to harmless chloride (Cl^−^). The reduction of ClO_4_^−^ to Cl^−^ is the only pathway to eliminate its environmental risks, but it is notoriously resistant to reduction under mild conditions. Despite its strong thermodynamic oxidizing potential (E^0^(ClO_4_^−^/Cl^−^) = 1.37 V), ClO_4_^−^ exhibits remarkable kinetic inertness due to two intrinsic molecular characteristics: (i) the high strength of Cl−O bonds (~400 kJ/mol)^8^ and (ii) the symmetric, charge-delocalized tetrahedral structure. Therefore, effective abiotic reduction of ClO_4_^−^ requires catalysts that can bond and abstract one O atom of ClO_4_^−^, thereby distorting the symmetrical geometry and weakening the Cl−O bond^9^.

However, this presents a fundamental challenge: catalytic sites must possess sufficient oxygen affinity to enable ClO_4_^−^ activation, while avoiding excessive metal−oxygen bond strength that leads to site poisoning by stable intermediates^10–12^. In aqueous media, noble metals are typically required either (i) to provide active sites for ClO_4_^−^ binding or (ii) to enable the reductive removal of oxygen atoms from intermediates formed on the catalysts, using H_2_ as the terminal electron donor (as in the Re-Pd/H_2_ system). These systems generally require acidic conditions (pH <4.0) to maintain activity^13,14^, whereas ClO_4_^−^ generally exists in neutral to alkaline environments, such as in wastewater and natural waters. Noble-metal-free catalysts (e.g., those based on Fe or Ti) operate under even harsher conditions, including low pH (pH <3.0)^15,16^ and elevated temperatures (*T* > 90 °C)^17^. Therefore, the practicality of current aqueous-phase catalytic systems is limited by the mismatch between their treatment conditions and actual contaminated water matrices.

In contrast, biological systems provide a blueprint for efficient ClO_4_^−^ reduction under mild conditions via Mo-dependent perchlorate reductase (PcrAB)^18^. The PcrAB features a Mo(IV) active site that catalyzes the key oxygen atom transfer (OAT) step in the reduction of ClO_4_^−^ to chlorate (ClO_3_^−^)^19^, followed by regeneration of the oxidized Mo sites through electron donation from electron-rich co-factors, such as Fe-S clusters^20,21^. This biological system underscores the feasibility of Mo-based sites, and its paradigm reveals two essential design principles for abiotic catalysts: (i) an accessible Mo active site with optimal affinity that enables ClO_4_^−^ bonding while allowing facile release of reduced products, and (ii) a robust electron donor to reduce the Mo site and sustain catalytic turnover.

To meet the demands of activation and electron delivery, we constructed a heterogeneous donor−acceptor (D−A) catalytic system, composed of defective MoS_2_ supported on N-doped carbon (MoS_2_−NC) and zerovalent iron (Fe^0^). The D−A catalyst concept has been widely adopted in molecular catalysis and organic photovoltaics for enabling spatial charge separation and directional electron flow^22–24^, which offers a suitable framework for addressing the challenges of ClO_4_^−^ reduction. In this system, Fe^0^ serves as a sacrificial electron donor (E^0^(Fe^2+^/Fe^0^) = −0.44 V), functionally analogous to Fe−S co-factors, while MoS_2_−NC acts as the electron acceptor and catalytic domain. The undercoordinated Mo sites exposed in defective MoS_2_ provide oxygen-affinitive centers for ClO_4_^−^ adsorption and activation, mimicking the functional geometry of PcrAB^21^. Simultaneously, the N-doped carbon facilitates efficient electronic coupling between Fe^0^ and the Mo active sites, promoting sustained electron injection while preventing over-reduction or site passivation. This D−A configuration enables spatial and electronic separation of reduction and activation functions, thereby satisfying the redox and structural demands of ClO_4_^−^ catalysis under mild conditions. By bridging bioinspired catalysis with the D−A design paradigm, our work offers a new strategy for noble-metal-free ClO_4_^−^ reduction. The MoS_2_−NC/Fe^0^ system operates effectively under mild conditions, offering practical implications for scalable water treatment and advancing the broader development of functional D−A heterogeneous catalysts for oxyanion conversion.

## Results

### Density functional theory analysis of the MoS_2_−NC/Fe^0^ system

To evaluate the design rationale of our MoS_2_−NC/Fe^0^ D−A system, we performed density functional theory (DFT) calculations to validate the hypothesis that this system can replicate the mechanistic steps of the OAT pathway proposed in biological systems (detailed in Supplementary Methods). The calculations focused on these key steps (electron transfer, ClO_4_^−^ adsorption, Cl−O cleavage, followed by O atom dissociation from reactive sites) and also aimed to clarify the role of each component in facilitating these processes. Accordingly, we constructed slab models representing MoS_2_, MoS_2_|NC, and MoS_2_|Fe^0^ and MoS_2_|NC|Fe^0^ to compare the electron migration behavior and reaction energetics across different systems (Supplementary Fig. 1).

**Fig. 1:**
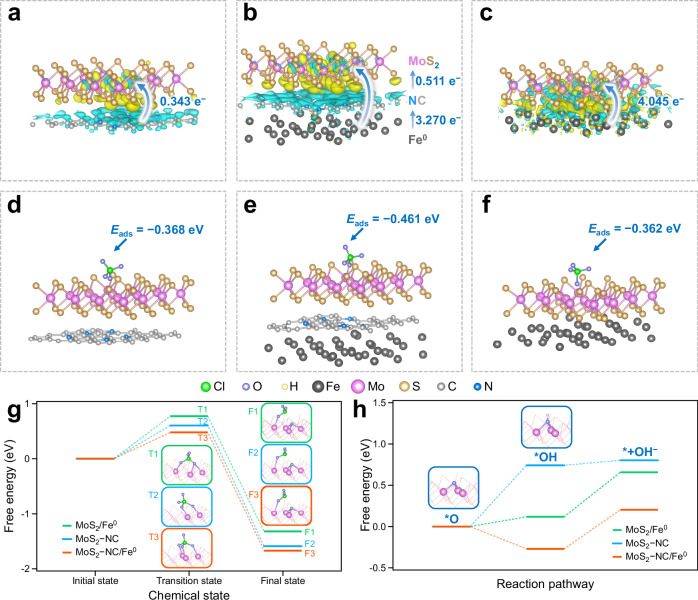
DFT calculations of MoS_2_−NC/Fe^0^. **a**–**c** Charge density difference plot for MoS_2_|NC (**a**), MoS_2_|Fe^0^ (**b**), and MoS_2_|NC|Fe^0^ (**c**) models. **d**–**f** Adsorption energies and configurations of ClO_4_^−^ on MoS_2_|NC (**d**), MoS_2_|Fe^0^ (**e**), and MoS_2_|NC|Fe^0^ (**f**). **g** Free energy barriers for first oxygen atom cleavage from adsorbed ClO_4_^−^ on different surfaces. **h** Energy diagrams for the dissociation of the O atom from the Mo sites at pH 6.0.

Density of states (DOS) analysis for the MoS_2_ slab alone confirms its semiconducting feature^25^, with the Fermi level located within the band gap (Supplementary Fig. 2). When combining with NC, electronic states appear near the Fermi level in the total DOS. Layer-projected DOS indicates that these near-Fermi states are not solely contributed by NC, but also by MoS_2_, which is attributed to the interfacial charge redistribution. Similar trends are also observed for MoS_2_|Fe^0^ and MoS_2_|NC|Fe^0^ models. To further investigate the electron migration behavior, we conducted charge density difference analysis and quantified net electron transfer by Bader analysis. The MoS_2_ layer receives 0.343 e^−^ (0.257 e^−^/nm^2^) in MoS_2_|NC and 0.511 e^−^ (0.383 e^−^/nm^2^) in MoS_2_|NC|Fe^0^, consistent with charge density difference plots (Figs. 1a and 1b) that show electron accumulation on MoS_2_ (yellow) and depletion in the NC layer (cyan). The charge density difference plot of MoS_2_|NC|Fe^0^ relative to NC (Supplementary Fig. 3) further shows that electrons donated from Fe^0^ accumulate primarily within the NC interlayer. Quantitatively, Bader analysis indicates that Fe^0^ donates 3.270 e^−^ to NC (2.453 e^−^/nm^2^), of which 0.511 e^−^ are transferred to MoS_2_ (0.383 e^−^/nm^2^) in MoS_2_|NC|Fe^0^. These results confirm that the electron transfer from Fe to the catalytic MoS_2_ domain is effectively mediated by the interlayer NC, consistent with the D−A design. In contrast, direct contact in MoS_2_|Fe^0^ yields a larger electron transfer of 4.045 e^−^ (3.034 e^−^/nm^2^) to MoS_2_ (Fig. 1c), but this does not result in improved ClO_4_^−^ activation (as discussed below), suggesting that catalytic promotion requires optimized electronic redistribution rather than simply increasing electron density of MoS_2_.

We then compared the adsorption energy of ClO_4_^−^ at undercoordinated Mo sites for the three models (Supplementary Fig. 4). Introducing Fe^0^ to MoS_2_|NC enhances ClO_4_^−^ adsorption, with adsorption energy decreasing from −0.368 eV to −0.461 eV (Fig. 1d, e). In comparison, despite the stronger electron transfer in MoS_2_|Fe^0^, ClO_4_^−^ adsorption is not facilitated (Fig. 1f). This can be attributed to the overly electron-rich environment in MoS_2_|Fe^0^ that results in electrostatic repulsion toward the anionic oxyanion (ClO_4_^−^)^26–28^. In comparison, the NC interlayer mediates electron transfer but avoids unfavorable electrostatic effects toward an anionic substrate, providing a more suitable electronic environment for ClO_4_^−^ binding. In addition, in all the models, ClO_4_^−^ preferentially adsorbs at undercoordinated Mo sites over S sites. For example, the ClO_4_^−^ adsorption energy on the S sites of MoS_2_|NC|Fe^0^ model is unfavorable ( + 1.917 eV).

To probe OAT reactivity, we calculated the energy barrier for the cleavage of the first Cl−O bond of adsorbed ClO_4_^−^ for the three models (Fig. 1g). In the absence of Fe^0^, MoS_2_|NC exhibits an energy barrier of 0.603 eV for O atom abstraction from ClO_4_^−^, whereas introducing Fe^0^ lowers this barrier to 0.479 eV for MoS_2_|NC|Fe^0^. By contrast, MoS_2_|Fe^0^ model exhibits the highest barrier among the three models. These results demonstrate that the Mo−O bond formation coupled with Cl−O cleavage for initiating OAT was most favorable in the MoS_2_|NC|Fe^0^ configuration. We further evaluated the subsequent dissociation of the Mo-bound O atom from Mo sites, which involves the hydrogenation of O atom (*O → *OH) and the release of OH^−^ (*OH → * + OH^−^) (Fig. 1h). In these steps, the MoS_2_|NC|Fe^0^ model shows the most favorable energetics, with the lowest Δ*G* for the hydrogenation step (−0.271 eV) and the total energy for the two step (0.203 eV). It should also be noted that although the dissociation energy of the O atom from Mo sites remains thermodynamically uphill for all three systems, the overall reaction free energy accounting for both Cl−O bond cleavage and its O atom dissociation is negative, particularly in MoS_2_|NC|Fe^0^, which indicates the ClO_4_^−^ reduction is thermodynamically favorable in the MoS_2_|NC|Fe^0^ model.

In addition, during structural optimization, in a test model constructed with fewer vacancies (two S atoms removed), adsorption at the more highly coordinated Mo sites led to spontaneous cleavage of the Cl−O bond, indicating an unrealistically barrierless pathway, whereas this behavior was not observed for our selected vacancy model (three S atoms removed). This suggests that an appropriate coordination environment is required to enable efficient ClO_4_^−^ reduction. Taken together, these DFT results demonstrate the functional role of the D−A configuration, where Fe^0^ enables sustained electron transfer, and MoS_2_ provides favorable adsorption and reactive Mo sites, while NC acts as an electronic mediator that optimizes (rather than maximizes) electron transfer to the catalytic domain, enhancing ClO_4_^−^ adsorption and promoting OAT energetics. These results provide theoretical support for the design rationale of MoS_2_−NC/Fe^0^ as a D−A system for efficient ClO_4_^−^ reduction.

### Characterization of the MoS_2_−NC catalysts

**Fig. 2:**
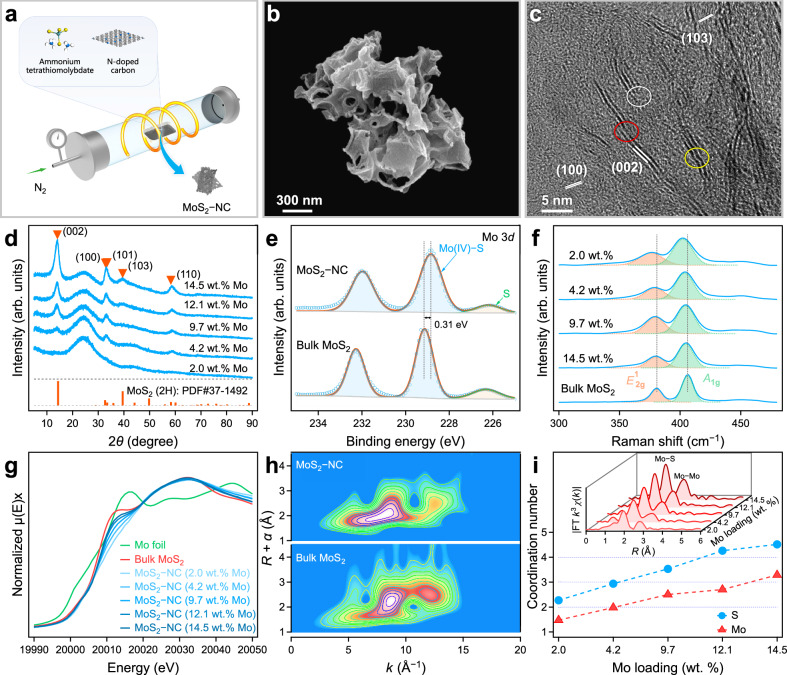
Characterization of MoS_2_−NC. **a** Illustration of the synthetic process of MoS_2_−NC. **b**, **c** SEM (**a**) and HRTEM (**c**) images of MoS_2_−NC. **d** XRD patterns of MoS_2_−NC with various Mo loadings. **e** XPS spectra for MoS_2_−NC and bulk MoS_2_. **f** Raman spectra of MoS_2_−NC and bulk MoS_2_. **g**–**i** XANES (**g**), wavelet transform (WT) of the EXAFS spectra (**h**), and Fourier-transformed (FT) *k*^3^-weighted EXAFS spectra (**i**) at the Mo *K*-edge of MoS_2_−NC and reference sample(s). The coordination numbers in **i** are obtained from the fitting of EXAFS spectra.

Based on the insights from the DFT predictions, we synthesized MoS_2_−NC catalysts featuring defective Mo sites and a NC support. The catalysts were prepared via a facile pyrolysis method using ammonium tetrathiomolybdate and NC as precursors under a N_2_ atmosphere (Fig. 2a). A series of MoS_2_−NC catalysts with varying Mo loading (2.0–14.5 wt.%) were obtained, as determined by inductively coupled plasma-optical emission spectrometry (ICP-OES). Detailed characterization of these materials was performed. Scanning electron microscopy (SEM, Fig. 2b) reveals that all MoS_2_−NC catalysts exhibit a similar crumpled, porous sheet-like morphology. Therefore, representative results for the catalyst with 4.2 wt.% Mo loading is shown for SEM and other morphological characterizations. Energy-dispersive X-ray spectroscopy (EDS, Supplementary Fig. 5) confirms the presence of C, N, Mo, and S in the catalyst. The transmission electron microscope (TEM) analysis further verifies the sheet-like morphology (Supplementary Fig. 5f), and the high-resolution TEM (HRTEM) image clearly resolves the characteristic lamellar fringes of MoS_2_ crystallites (Fig. 2c), consistent with their expected crystal structure. Furthermore, the HRTEM image also displays a transition between lattice domains, distorted lattice fringes, and discontinuous fringes, marked by red, yellow, and white circles, respectively, suggesting the structural disorder and defective domains of the MoS_2_ phase^29,30^.

X-ray diffraction (XRD) patterns (Fig. 2d) show broad humps around ~26°, which can be ascribed to the (002) plane of graphite-like NC support^31^. In addition, the intensity of peaks corresponding to the MoS_2_ (2H phase) increases with increasing Mo loading, confirming the formation of crystalline MoS_2_. Raman spectra (Fig. 2e) further support the presence of MoS_2_ and exhibit characteristic peaks of MoS_2_ at ~378 cm^−1^, and ~404 cm^−1^, corresponding to the in-plane *E*^1^_2g_ and out-of-plane *A*_1g_ vibration modes of MoS_2_^32,33^, respectively. Compared to bulk MoS_2_, both peaks exhibit clear redshifts, which could be ascribed to the defects in the MoS_2_ lattice^34^. These redshifts may result from the overlapping of various defect-induced vibration modes^35^. Lower Mo loading leads to more pronounced redshifts and broader peaks, indicating a higher density of defects^36^. X-ray photoelectron spectroscopy (XPS, Fig. 2f) spectra of Mo 3 *d* show a doublet peak at ~229 eV (Mo(IV) 3*d*_5/2_.) and ~232 (Mo(IV) 3*d*_3/2_). The 3*d*_5/2_ peak shows a significant 0.31 eV relative to bulk MoS_2_, demonstrating that defects alter the electronic structures of Mo atoms and increase the local electron density^34,37^. Moreover, the C 1 *s* spectrum of MoS_2_−NC exhibits the same peak shape and position as bulk MoS_2_, with no feature attributable to Mo−C (or C−S) bonds. Comparison of N 1 *s* spectra of MoS_2_−NC, together with the fitting results of MoS_2_−NC spectrum, shows no detectable Mo−N bond (Supplementary Fig. 6). Therefore, the absence of detectable Mo−C/Mo−N bonds implies that the interfacial contact between MoS_2_ and NC is dominated by noncovalent interactions (likely via van der Waals forces). Meanwhile, the DFT results indicate interfacial charge redistribution, implying that this predominantly noncovalent interface still involves electronic coupling between two components.

X-ray absorption near-edge structure (XANES) and extended X-ray absorption fine structure (EXAFS) spectra at Mo *K*-edge were collected to probe the atomic coordination environment. The XANES absorption edge position of MoS_2_−NC (determined by the maximum of the first derivative) is consistent with that of bulk MoS_2_, indicating a Mo oxidation state of +4 across all Mo loadings (Fig. 2g). Meanwhile, the characteristic feature at ~20010 eV becomes more pronounced with increasing Mo loading, verifying the emergence of bulk MoS_2_-like structures^33^, which aligns with the XRD results. Wavelet transform (WT) analysis of the EXAFS spectra was employed to further elucidate the local coordination environment of the Mo atom. As shown in Fig. 2h, the WT−EXAFS spectrum of bulk MoS_2_ exhibits primary and secondary maxima at 8.7 Å^−1^ and 12.1 Å^−1^, corresponding to Mo−S and Mo−Mo scattering paths, respectively. MoS_2_−NC displays similar spectral features at ~8 Å^−1^ and ~12 Å^−1^, confirming analogous scattering paths to those of MoS_2_. These results confirm that the two prominent peaks in Fourier-transformed (FT) *k*^3^-weighted EXAFS spectra can be attributed to Mo−S and Mo−Mo coordination, and their intensities increase with increasing Mo loading (Fig. 2i).

Quantitative EXAFS fitting further confirms that the interatomic Mo−S and Mo−Mo distances are consistent with those of bulk MoS_2_ (Supplementary Fig. 7 and Table S1)^38^. Nevertheless, the corresponding CNs (Fig. 2i) are significantly lower than those of bulk MoS_2_, which are 6 for both first-shell S and second-shell Mo. This indicates the presence of uncoordinated Mo sites associated with defects^39,40^. The CNs increase with increasing Mo loadings, implying a decrease in defect density, which is consistent with the Raman results. Together, these results confirm that the MoS_2_ on NC supports contains abundant defects and that the defect density can be tuned by adjusting the Mo loading.

### ClO_4_^−^ reduction in MoS_2_−NC/Fe^0^ systems

**Fig. 3:**
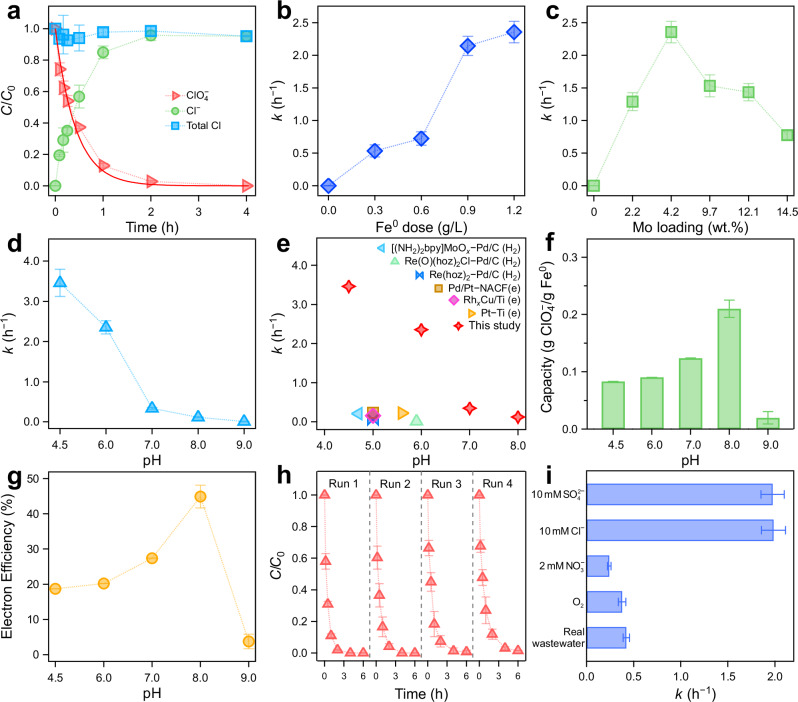
Catalytic performance of MoS_2_−NC for ClO_4_^−^ reduction. **a** Reduction kinetics of ClO_4_^−^ in MoS_2_−NC/Fe^0^ system and the generation of its products at pH 6.0. The solid line is the pseudo-first-order fit. **b**–**d** Influences of Fe^0^ dose (**b**), Mo loading (**c**), and pH (**d**) on the pseudo-first-order rate constants. **e**, Comparison of the ClO_4_^−^ reduction rates in MoS_2_−NC/Fe^0^ systems with other aqueous catalytic systems at pH range of 4.0 to 8.0^14,44,63–68^. **f**, **g** Capacity (**f**) and electron efficiency (**g**) of Fe^0^ for ClO_4_^−^ reduction. **h** ClO_4_^−^ reduction kinetics in four cycles. **i** Influence of water matrix on ClO_4_^−^ reduction. Reaction conditions: [ClO_4_^−^]_0_ = 1.0 mM, [Fe^0^]_0_ = 1.2 g/L, [MoS_2_−NC] = 4.0 g/L, the Mo loading is 4.2 wt.% except as denoted in (**b**), pH is 6.0 except as denoted in **d**–**g**, pH is maintained with 150 mM buffer, *T* = 25 °C. Error bars represent standard deviations from two independent experiments.

To construct the D−A catalytic system, Fe^0^ and MoS_2_−NC were combined by simple mixing (Supplementary Fig. 8). The Fe^0^ used here was synthesized via borohydride reduction, resulting in nanoscale zerovalent iron (known as nZVI) particles. These particles were characterized by TEM, XRD, and XPS, and the corresponding results provided in the Supplementary Figs. 9 and 10, which are consistent with commonly reported nZVI properties^41–43^. This system combining Fe^0^ and MoS_2_−NC enabled efficient ClO_4_^−^ reduction, whereas systems containing only Fe^0^ or only MoS_2_−NC showed negligible adsorption or reactivity toward ClO_4_^−^ (Supplementary Fig. 11). Reduction kinetics at pH 6.0 were measured under varying Fe^0^ and MoS_2_−NC doses, as well as different Mo loadings, and representative results are presented in Fig. 3a. Cl^−^ was the only detectable product, and the mass balance accounting for ClO_4_^−^ and Cl^−^ showed a small deficit (< 10%) in the early stage of the reaction, which gradually recovered to ~100%, implying transient adsorption of intermediates. The disappearance of ClO_4_^−^ followed pseudo-first-order kinetics under all tested conditions. The rate constants (*k*) increased with increasing Fe^0^ dose (0.3–1.2 g/L, Fig. 3b and Supplementary Fig. 12) and MoS_2_−NC dose (0.5–4.0 g/L, Supplementary Fig. 13). We did not further increase their doses to remain within the typical concentration range. Under the selected condition (1.2 g/L Fe^0^ and 4.0 g/L MoS_2_−NC), the effect of Mo loading in MoS_2_−NC was evaluated (Supplementary Fig. 14). The rate constants initially increased with Mo loading but decreased beyond 4.2 wt.% (Fig. 3c). This suggests that excessive Mo loading reduces defect density, thereby diminishing catalytic performance. To better reflect intrinsic activity, rate constants were normalized by Mo content (*k*_Mo_) and correlated with the CN of Mo−S (Supplementary Fig. 15). *k*_Mo_ declined when the CN increases above ~3, indicating that a more undercoordinated (defect-rich) Mo environment is associated with more efficient ClO_4_^−^ reduction. At the optimal Mo loading of 4.2 wt.%, the rate constant for ClO_4_^−^ reduction was 2.36 h^−1^ (Fig. 3a). Over 95% of 1.0 mM ClO_4_^−^ was reduced within 2 h, and the concentration dropped below the detection limit (~1 μg/L or ~0.01 μM) within 4 h. In addition, varying Fe^0^ particle size (220–1555 nm) showed that the ClO_4_^−^ reduction rate decreases with increasing Fe^0^ particle size (Supplementary Figs. 16 and 17). This trend suggests that Fe^0^ surface availability is a critical factor for the reactivity, further supporting the role of Fe^0^ as the direct electron donor in this system.

The reactivity of the MoS_2_−NC/Fe^0^ system toward ClO_4_^−^ decreased as pH increased from 4.5 to 9.0 (Fig. 3d and Supplementary Fig. 18), similar to the trends for other reductive systems^14^. This pH dependence likely arises from several factors. First, H^+^ may (i) serve as the source of reducing species (e.g., atomic H, a known possible secondary reductant generated from Fe^0^)^44^, or (ii) directly participate in the hydrodeoxygenation pathway, as discussed below. Second, higher pH leads to a more negative zeta potential (Supplementary Fig. 19), which reduces the electrostatic affinity of the catalyst surface to ClO_4_^−^ anions. In addition, Mo leaching was negligible across the tested pH range (Supplementary Fig. 20), ruling it out as a factor of activity loss. Despite these pH effects, the MoS_2_−NC/Fe^0^ system exhibited high activity under near-neutral to mildly basic conditions. Notably, all experiments were conducted under mild conditions, i.e., without high temperatures or acidic conditions. To our knowledge, this is the first non-noble metal system to achieve such high ClO_4_^−^ reduction rates in water under near-neutral conditions, outperforming even noble metal catalysts tested under comparable conditions (Fig. 3e and Supplementary Table 2).

During the reaction, the concentrations of dissolved Fe^2+^ and Fe^3+^ from Fe^0^ were synchronously monitored (Supplementary Fig. 21). Dissolved Fe^3+^ was ignorable in all cases, while Fe^2+^ appeared more rapidly at a lower pH. Based on the quantification for both dissolved and solid-phase Fe species generated from Fe^0^ (Supplementary Fig. 22), we calculated the specific capacity (mmol ClO_4_^−^/g Fe^0^) and electron efficiency at the end of reaction (Fig. 3f, g). The electron efficiency, which is defined as the fraction of Fe^0^-donated electrons that were used for ClO_4_^−^ reduction (rather than side reactions such as hydrogen evolution), ranged from 20.2 to 44.5% at near-neutral pH values (6.0–8.0). These high efficiencies imply that Fe^0^ doses only 2.2–5.0 times the molar amount of ClO_4_^−^ are needed to meet treatment goals, suggesting that the system remains practical for application even under less favorable (higher pH) conditions.

We evaluated the stability of MoS_2_−NC via cycling tests (Fig. 3h). Although a slight loss of activity was observed over repeated cycles, ClO_4_^−^ was still reduced to below detection limit within 6 h during the first four cycles. During the cycling tests, 4.87 mM ClO_4_^−^ was reduced with Cl^−^ as the only detectable product. Based on these results, the turnover number for Mo sites was estimated to be ~3, assuming a 1:1 stoichiometry between Mo and ClO_4_^−^. It should be noted that this turnover number was underestimated, because only a small fraction of total Mo atoms are exposed as surface catalytic sites. The influence of water matrices on the performance was also examined (Fig. 3i and Supplementary Fig. 23). Sulfate (10 mM) and chloride (10 mM) showed little impact on the reduction rates of ClO_4_^−^, even at concentrations exceeding those typically detected in natural waters or wastewater. Nitrate lowered the reduction rate by ~ninefold, due to its competition effect for electrons from Fe^0^. This competition effect was directly evidenced by the markedly accelerated Fe^2+^ release in the presence of nitrate (Supplementary Fig. 24), demonstrating increased Fe^0^ consumption that consequently reduced the electron availability for ClO_4_^−^ reduction. Similarly, O_2_ inhibited ClO_4_^−^ reduction when the reactor was open to the air. However, this inhibitory effect was less severe compared to other systems, which typically require fully deoxygenated conditions for efficient reduction^45^. Furthermore, we assessed the effectiveness of the MoS_2_−NC/Fe^0^ system in real wastewater without any pretreatment. The system achieved a rate constant of 0.377 h^−1^ and the ClO_4_^−^ concentration dropped from 2.1 mM to below the detection limit in 8 h, at the initial pH of 6.2. Although further investigation is needed to address catalyst deactivation and interference from competing oxidants, these results demonstrate that MoS_2_−NC/Fe^0^ systems can achieve desirable performance even under challenging conditions.

### Catalytic mechanisms of ClO_4_^−^ reduction

**Fig. 4:**
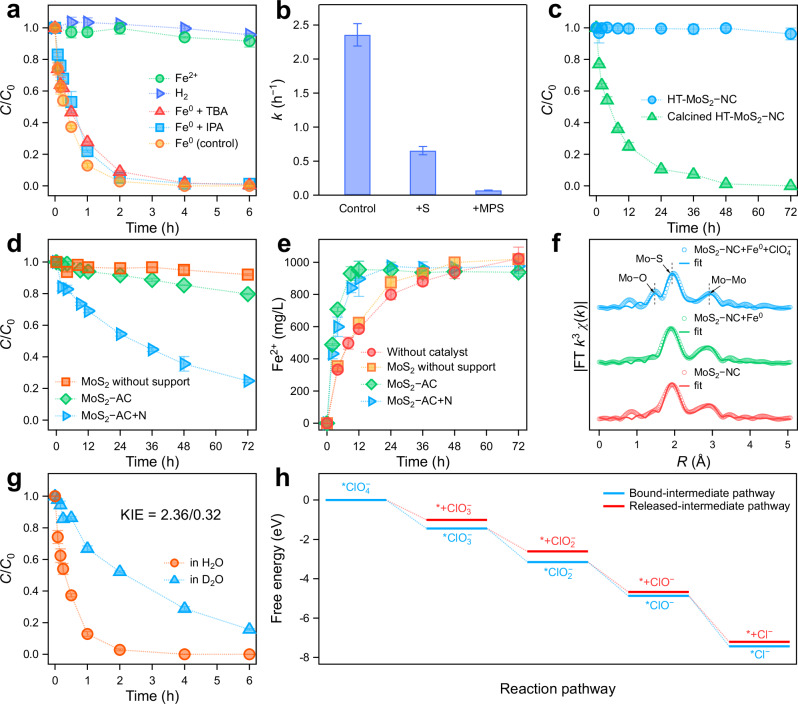
Catalytic mechanism of ClO_4_^−^ reduction in MoS_2_−NC/Fe^0^ system. **a** ClO_4_^−^ reduction kinetics with Fe^2+^ and H_2_ as the electron donor, and with Fe^0^ in the presence of *tert*-butanol (TBA, a scavenger for atomic H) and isopropanol (IPA, source of atomic H). **b** Influence of S addition and (3-mercaptopropyl)trimethoxysilane (MPS, a repair agent for S vacancies) pretreatment to MoS_2_−NC on ClO_4_^−^ reduction rate constants. **c** Kinetics of ClO_4_^−^ reduction catalyzed by hydrothermally synthesized MoS_2_ on NC (HT-MoS_2_−NC) and its calcined counterpart. **d**, **e** Influence of supports on the catalytic activity of MoS_2_ (**d**) and Fe^2+^ release from Fe^0^ (**e**), where AC denotes activated carbon. **f** EXAFS spectra of MoS_2_−NC during reaction. **g** Influence of D_2_O on ClO_4_^−^ reduction, where KIE denotes the kinetic isotope effect. **h** Energy diagrams and intermediate states of ClO_4_^−^ reduction in MoS_2_−NC/Fe^0^ system. Reaction conditions: [ClO_4_^−^]_0_ = 1.0 mM, [Fe^0^]_0_ = 1.2 g/L for a and b, [Fe^0^]_0_ = 1.0 g/L for **c**–**e**, [catalyst] = 4.0 g/L, the Mo loading is 4.2 wt.% for all catalysts, pH is maintained at 6.0 with 150 mM buffer, *T* = 25 °C. Error bars represent standard deviations from two independent experiments.

In previous studies^46,47^, Fe^0^ has been proposed to donate electrons directly or indirectly via secondary products, including Fe^2+^, H_2_, and atomic H. To identify the electron donor responsible in the MoS_2_−NC/Fe^0^ system, control experiments were conducted using Fe^2+^ and H_2_ alone (Fig. 4a). Neither Fe^2+^ or H_2_ could reproduce the reduction activity achieved with Fe^0^ under identical conditions. Additional experiments with *tert*-butanol (a scavenger of atomic H) and isopropanol (a source of atomic H)^48^ showed negligible effects on ClO_4_^−^ reduction kinetics or product distribution. These results collectively exclude atomic H-mediated pathways. Therefore, reduction in this system proceeds through an OAT mechanism, with Fe^0^ serving as the direct electron donor. To provide direct evidence for this electron transfer mechanism, a two-compartment galvanic cell was constructed using Fe^0^ as the anode, and MoS_2_−NC as the cathode (Supplementary Fig. 25)^47^. The anode and cathode compartments were separated by a proton exchange membrane, with the electrodes connected via an external Ti wire. ClO_4_^−^, present exclusively in the cathode compartment at an initial concentration of 1 mM, achieved 23.6% reduction over 72 h, confirming that the electrons were supplied solely by Fe^0^. All lines of evidence demonstrate that Fe^0^ serves as the direct electron donor for ClO_4_^−^ reduction in this system. The Fe^2+^, as the main product released from Fe^0^, can be used to probe the Fe^0^ interactions with the MoS_2_−NC.

We further conducted experiments to identify the catalytic sites of MoS_2_−NC. As shown in Supplementary Fig. 14a, NC without Mo loading did not show any catalytic activity, indicating that MoS_2_ in the catalyst provides the active sites. Due to the ineffectiveness of bulk MoS_2_, the DFT predictions and the characterization of defect structures, it is hypothesized that the defects of MoS_2_ are responsible for ClO_4_^−^ reduction. These defects, mainly S vacancies, could expose undercoordinated Mo sites and adjacent S sites^49^. To identify their role, S powder was added during catalyst synthesis to increase the abundance of S sites and coordinate Mo sites. This treatment significantly suppressed activity (Fig. 4b and Supplementary Fig. 26), suggesting that the exposed undercoordinated Mo sites, not S sites, are responsible for catalysis. We further pretreated MoS_2_−NC with a targeted agent to repair S vacancies and thereby to increase Mo coordination, i.e., (3-mercaptopropyl)trimethoxysilane (MPS)^50^. MPS pretreatment strongly deactivated the MoS_2_−NC, confirming that S vacancies that expose Mo atoms are the catalytic sites. In addition, Fe^2+^ release remained unaffected by these treatments (Supplementary Fig. 27), ruling out their interference with the role of Fe^0^ in electron donation.

To provide more evidence for the role of Mo sites in defective MoS_2_, we synthesized a catalyst by growing MoS_2_ on NC support with a hydrothermal method (noted as HT-MoS_2_−NC)^51^. The as-prepared HT-MoS_2_−NC showed no activity. In contrast, upon calcination to remove S atoms to induce S vacancies, the material exhibited excellent catalytic activity (Fig. 4c). The electron paramagnetic resonance (EPR)^52^ spectroscopy confirmed the presence of S vacancies in both MoS_2_−NC and calcined HT-MoS_2_−NC (Supplementary Fig. 28). Taken together, these results demonstrate that the activity of MoS_2_−NC arises from defective MoS_2_, where S vacancies create undercoordinated Mo sites that serve as the active centers for ClO_4_^−^ reduction. Together with the role of Fe^0^ as the direct electron donor, these results provide experimental validation for the D−A catalytic configuration designed in this study.

To elucidate the role of the NC support beyond structural stabilization of MoS_2_, additional experiments were conducted. First, MoS_2_ without any support exhibited negligible catalytic activity (Fig. 4d), whereas the incorporation of the NC support, even at a low content (~50 wt.%), significantly enhanced ClO_4_^−^ reduction activity (Supplementary Fig. 14e). These roles are further supported by control experiments (Supplementary Fig. 29). Although ball milling can introduce defects into MoS_2_, ball-milled MoS_2_ (without NC) alone showed negligible ClO_4_^−^ removal upon Fe^0^ addition. In contrast, co-milling MoS_2_ with NC enabled measurable ClO_4_^−^ reduction in the presence of Fe^0^, indicating that NC is required for the catalytic reactivity of defect-rich MoS_2_ in this system.

During the reaction, the Fe^2+^ release in the system with unsupported MoS_2_ was very similar to that without any material (Fig. 4e), and was significantly slower than that observed with MoS_2_−NC (Supplementary Fig. 30). Since Fe^2+^ is almost the sole product of Fe^0^ oxidation under these conditions, its release rate reflects the rate of electron donation from Fe^0^. Mo loading did not affect Fe^2+^ release, but significantly influenced the rate of ClO_4_^−^ reduction, indicating that the electron-donating behavior of Fe^0^ is governed by the NC support. Notably, the similar Fe^2+^ release in the presence of unsupported MoS_2_ does not imply that it cannot receive electrons; rather, it likely reflects the lack of effective conductive contact or electron-consumption pathway without NC. These observations validate the role of NC as a mediator that supports the electron transfer and enables efficient utilization of Fe^0^-derived electrons for ClO_4_^−^ reduction at the MoS_2_ domain. To further explore the support effect, MoS_2_ was loaded onto activated carbon (AC). Although the catalyst with AC support (noted as MoS_2_−AC) exhibited an enhanced Fe^2+^ release rate (Fig. 4e), it showed only weak ClO_4_^−^ reduction activity (Fig. 4d), indicating that AC can mediate electron transfer, but MoS_2_−AC does not provide enough active sites. In contrast, N doping (MoS_2_−AC + N) significantly enhanced the activity, while the Fe^2+^ release was not promoted. This suggests that N doping modifies the MoS_2_ active sites rather than affecting electron mediation. Although XPS results validated the absence of the Mo−N bond in this study (likely due to N stripping during high-temperature synthesis), previous studies have demonstrated that N doping increases S vacancies by facilitating Mo−N bond formation^53,54^. To provide direct experimental support, we performed EPR analysis, which revealed a stronger S vacancies signal for MoS_2_−AC + N compared to MoS_2_−AC (Supplementary Fig. 31), confirming that N doping increases S-vacancy concentrations. This suggests that the NC support fulfills two key roles: it serves as an electron acceptor to facilitate electron transfer, and its N atoms increase the S vacancies in MoS_2_. These dual roles of NC further reinforce the functional separation of electron donation and catalytic activation that defines the D−A system.

We employed semi-in situ XAFS to track the dynamic evolution of Mo coordination environments during the reaction. The emergence of a distinct peak at ~1.5 Å in the EXAFS spectra during ClO_4_^−^ reduction directly confirmed the formation of Mo−O bonds (Fig. 4f), consistent with the DFT results (Fig. 1e). The Mo-bound O atom is hypothesized to undergo hydrodeoxygenation, releasing OH^−^ via a stepwise hydrogenation process (*O → *OH → * + OH^−^). To experimentally validate this proposed mechanism, we further evaluated the solvent kinetic isotope effect (KIE) by comparing reaction rates in H_2_O and D_2_O systems (Fig. 4g). A significant decrease in the reduction rate (KIE = 7.38) was observed, while Fe^2+^ release remained largely unchanged (Supplementary Fig. 32), indicating the involvement of H^+^ in the OAT process. Integrating experimental observations with DFT calculations, we propose a surface-bound reaction mechanism (Fig. 4h, blue path) comprising three key steps: initial adsorption of ClO_4_^−^ at undercoordinated Mo sites, O atom abstraction via Cl−O bond cleavage (*ClO_4_^−^→*ClO_3_^−^+*O), and sequential hydrogenation of the Mo-oxo species (*O → *OH → OH^−^). The deoxygenated, surface-bound intermediates (*ClO_*x*_) undergo further reduction until the release of Cl^−^. This bound-intermediate pathway was supported by the calculated free energy diagram, where it is more thermodynamically favorable than the released-intermediate one (Fig. 4h, red path), aligning with the experimental observations that Cl^−^ is the sole product and no ClO_*x*_ species are detected.

### Technoeconomic analysis for the MoS_2_−NC/Fe^0^ system

A preliminary technoeconomic analysis (TEA) was conducted to compare the MoS_2_−NC/Fe^0^ system with representative Pd/H_2_-based catalytic systems for ClO_4_^−^ reduction. They share a similar conceptual process design (fixed-bed or slurry contactor followed by solids separation), making the Pd/H_2_-based systems the most representative comparator among abiotic ClO_4_^−^ reduction approaches. In Pd/H_2_-based systems, the typical catalyst loading is 0.2 g/L containing 5 wt.% Pd and 5 wt.% Re or Mo^14,55^, operating under 1 atm H_2_ at pH 3. For the MoS_2_−NC/Fe^0^ system, we evaluated a coarser Fe^0^ powder (20 mesh) to reflect practical cost considerations. At 4.0 g/L MoS_2_−NC (4.2 wt.% Mo) with 12 g/L Fe^0^, the system achieved ~80% ClO_4_^−^ removal in 18 h (Supplementary Fig. 33), and all costs were normalized to an equivalent basis (per 1 mM removed), and the detailed TEA is provided in Supplementary Note 1.

On a 1 L treatment basis (1 mM initial ClO_4_^−^), a consumables-based evaluation using bulk commercial pricing yields a total materials cost of $0.084–$0.229 per L for MoS_2_−NC/Fe^0^, versus $4.84–$7.35 per L for the typical Pd/H_2_-based comparator when including typical acidification/neutralization and H_2_ supply. The cost of MoS_2_−NC/Fe^0^ is dominated by Fe^0^ and the N-doped carbon fraction, while the Pd/H_2_-based system cost is dominated by the noble-metal catalyst and required ligand. Since these values represent a first-run estimate (i.e., full material inputs charged to a single treatment run), we further extended the analysis to operating expenditure (OPEX) for continuous operation on a 1 mM removed per m^3^ basis. Under the assumptions described in the Supplementary Information, MoS_2_−NC/Fe^0^ is estimated at $6.27–$28.25 per m^3^, dominated by net Fe^0^ consumption (Fe^0^ supplementation) and slurry management^56,57^, whereas Pd/H_2_ is estimated at $76.0–$116.8 per m^3^, dominated by catalyst supplementation, with H_2_ and pH-control costs. It should be noted that these estimates should be interpreted as order-of-magnitude benchmarks, instead of absolute values, because pricing and operating conditions are site-specific and the calculations adopt conservative assumptions on losses and deactivation, but the results consistently indicate a cost advantage for MoS_2_−NC/Fe^0^ under practical pricing.

From a capital expenditure (CAPEX) perspective, the MoS_2_−NC/Fe^0^ system is expected to be driven mainly by reactor volume (long hydraulic retention time (HRT)) and solids separation/dewatering infrastructure^58–60^. In comparison, the Pd/H_2_-based system has the advantage of shorter HRT but requires H_2_ supply and safety systems to prevent the accumulation of flammable H_2_ mixtures, which increase mechanical and permitting complexity. In addition, the acid/base storage and dosing infrastructure contributes substantially to the CAPEX. Overall, the MoS_2_−NC/Fe^0^ system offers advantages in safety and operational simplicity (near-neutral pH, no combustible gas), supporting its potential for cost-effective and scalable ClO_4_^−^ treatment. In future studies, the Fe^0^ could be replaced by an electrochemical cathode as the electron donor, which could further increase the cost-effectiveness by reducing Fe^0^ consumption, solid generation, and the associated slurry management.

## Discussion

In conclusion, we have developed the first noble-metal-free system, MoS_2_−NC/Fe^0^ for efficient ClO_4_^−^ reduction in water under mild conditions. MoS_2_−NC serves as the electron acceptor and catalytic domain, while Fe^0^ serves as the sacrificial electron donor. The system exhibited a rate constant as high as 2.36 h^−1^ at near-neutral pH, achieving over 95% removal of ClO_4_^−^ in 2 h, with Cl^−^ identified as the only product. This performance surpasses that of noble metal catalysts under comparable conditions, without requiring acidic pH or elevated temperatures, and only 2.2–5.0 times the molar amount of Fe^0^ relative to ClO_4_^−^ is needed. Mechanistic investigations and DFT calculations revealed that undercoordinated Mo atoms in defective MoS_2_ serve as the active sites, while Fe^0^ acts as the electron donor. The NC support of MoS_2_−NC mediates the electron transfer from Fe^0^ to the active sites, and optimizes the electronic environment for ClO_4_^−^ reduction. Together, these findings validate our D−A catalytic design, in which electron donation by Fe^0^ and substrate activation at defective Mo sites are spatially and electronically coupled through the NC support. The reduction proceeds via an OAT mechanism, in which Cl−O bonds in ClO_4_^−^ are cleaved, and the resulting O atoms bind to Mo sites, followed by hydrodeoxygenation of the Mo-bound O atoms to regenerate the active sites. This work not only demonstrates a highly effective and practical strategy for ClO_4_^−^ reduction, but also provides design principles for developing defect-engineered catalysts for the reduction of persistent oxyanions.

## Methods

### Chemicals

All chemicals employed in this study were used without purification, mainly including ferrous sulfate heptahydrate (analytical reagent, Aladdin), sodium borohydride (98%, Sinopharm), D-(+)-glucose (ACS grade, Aladdin), ammonium tetrathiomolybdate (99.95%, Aladdin), melamine (99%, Aladdin), cyanuric acid (98%, Macklin), ethanol (99.7%, Macklin), acetic acid (99.5%, Aladdin), 2-(*N*-morpholino)ethanesulfonic acid (MES, 99%, Aladdin), *N*-(2-hydroxyethyl)piperazine-*N*′−2-ethanesulfonic acid (HEPES, 99%, Aladdin), tris(hydroxymethyl)-aminomethane (Tris, 99%, Macklin), sodium hydroxide (NaOH, 96%, Aladdin), sodium perchlorate monohydrate (AR, Sinopharm). All solutions were prepared with deoxygenated deionized (DO/DI) water in an anaerobic chamber (100% N_2,_ O_2_ < 0.8 ppm), unless otherwise specified. DO/DI water was prepared by sparging Milli-Q water with N_2_ for at least 0.5 h and left in the anaerobic chamber overnight.

### Materials synthesis

Fe^0^ was prepared via the conventional borohydride-reduction method, which yields nanoscale Fe^0^ particles. Briefly, a sodium borohydride solution was added dropwise to 100 mL of 17.86 mM ferrous sulfate solution under vigorous stirring. The amount of borohydride was twice the stoichiometric requirement for the complete reduction of ferrous ion to Fe^0^. Then the Fe^0^ particles were separated by vacuum filtration and washed thoroughly with DO/DI water to remove impurities in the solution. Finally, the particles were re-dispersed in DO/DI water to prepare Fe^0^ suspensions for further use. All these procedures were conducted in the anaerobic chamber. The synthesized Fe^0^ particles exhibited a mean particle size of 220 nm and a specific surface area of 3.74 m^2^/g. These particles were further characterized by TEM, XRD, and XPS, with the corresponding results provided in the Supplementary Information.

For MoS_2_−NC synthesis, 0.0471 g ammonium tetrathiomolybdate and 0.4 g N-doped carbon were dispersed in 15.0 mL DI water in a polytetrafluoretyhylene (PTFE) beaker, in a typical synthesis. The mixture was freeze-dried for 48 h and the dried solid was ground to obtain the precursor. The precursor was heated at the rate of 5 °C/min to 600 °C under a gas flow of 0.2 L/min N_2_ and pyrolyzed at this temperature for 2 h. The resulting product was ground to obtain MoS_2_−NC. The catalysts with different MoS_2_ loading were similarly synthesized by a similar procedure, except for adding different amounts of ammonium tetrathiomolybdate into the precursor solution. N-doped carbon without MoS_2_ (denoted as NC) was prepared by the same procedure but without ammonium tetrathiomolybdate.

C_3_N_4_ was synthesized using cyanuric acid and melamine complex as the precursor^61,62^. Cyanuric acid (4 g) and melamine (4 g) were mixed in 400 mL of ethanol for 0.5 h, and the mixture was sonicated for 0.5 h to develop the cyanuric acid−melamine complex. After centrifugation at 11,300×*g* for 5 min, the supernatant was discarded, and the precipitate was resuspended in ethanol again. This wash process was repeated three times, and the resulting white powders were dried at 60 °C in a vacuum oven and calcined at 550 °C for 6 h under nitrogen conditions with the heating rate of 2.3 °C/min. The resulting sample was ground using a mortar for further use. For the preparation of N-doped carbon used in the catalyst precursor, 1.2 g glucose and 0.8 g C_3_N_4_ were mixed in water in a PTFE beaker. This mixture was freeze-dried and pyrolyzed with the same protocol as that elaborated above for the catalyst.

### Characterization

SEM images were acquired using a Zeiss SIGMA HD electron microscope operated at an accelerating voltage of 5.0 kV with an InLens detector and a working distance of 7.4 mm. Energy-dispersive X-ray spectroscopy (EDS) was performed using an Oxford Instruments X-Max detector under an accelerating voltage of 15.0 kV. For SEM/EDS analyses, the catalyst samples were ultrasonically dispersed in ethanol, drop-cast onto clean Si wafers, and dried at room temperature. Transmission electron microscopy (TEM), high-angle annular dark-field scanning TEM (HAADF-STEM), and EDS elemental mapping of Fe^0^ particles were carried out using a FEI Talos F200x microscope operated at an accelerating voltage of 200 kV and equipped with a Super-X EDS system. For MoS_2_−NC, STEM was performed using a JEOL JEM-2100F operated at 200 kV. All TEM samples were prepared by ultrasonic dispersion in ethanol, drop-casting onto carbon-coated copper grids, and air drying.

X-ray diffraction (XRD) patterns were recorded on a Rigaku DXR-8000 diffractometer using Cu Kα radiation (40 kV, 40 mA) with a diffracted-beam graphite monochromator. Data were recorded over 2*θ* of 5°−90° with a step size of 0.01° and a scan rate of 5°/min. X-ray photoelectron spectroscopy (XPS) was conducted using a Thermo Scientific K-Alpha+ instrument equipped with a monochromated Al Kα X-ray source (1486.6 eV, 12 kV). High-resolution spectra were collected with a pass energy of 50 eV and a step size of 0.1 eV. Raman spectra were collected on a LabRAM HR Evolution confocal Raman microscope (Horiba) with a 514 nm excitation laser, and the spectral range was 50–500 cm^−1^. Zeta potentials were measured on a Zetasizer NANO-ZS (Malvern Panalytical) using electrophoretic light scattering (laser Doppler micro-electrophoresis, M3-PALS). Samples were dispersed in the same buffer solutions used in the batch experiments to maintain pH (provided below), and the suspension was loaded into a disposable folded capillary cell (DTS1070) using a syringe.

X-ray Absorption Fine Structure (XAFS) spectra were collected in transmission mode at beam line BL11B of the Shanghai Synchrotron Radiation Facility (SSRF), China. For the semi-in situ XAFS spectra, the MoS_2_−NC (4.2 wt.%) was collected on a 0.22-μm membrane filter after the reaction was carried out for 60 min. The sample was placed on an aluminum sample holder and sealed using Kapton tape film. The procedures of the experiments for collecting samples for XAFS analysis were similar to those for catalytic performance, except that the concentration of ClO_4_^−^ was 5.0 mM.

### Experimental procedures for ClO_4_^−^ reduction

In the anaerobic chamber, 36 mL solution contained ClO_4_^−^ and buffer, 4 mL suspension of Fe^0^, and a certain amount of MoS_2_−NC was added into a 60 mL serum bottle (Supplementary Fig. 8). Then the bottle was crimp sealed with a PTFE-coated septum, which would not adsorb any reactant or product in the bottle. The resulting buffer concentration of sodium acetate (for pH 4.5), HEPES (for pH 7.0 and 8.0), and Tris (for pH 9.0) were 150 mM and the pH could be maintained (pH change was less than 0.2) during the reaction. Then, the bottle was moved into a dark incubator (25 °C) and placed on a rotator (60 rpm, with a vertical plane). At gradually increasing time intervals, the bottle was taken down from the rotator into the anaerobic chamber for sampling. 1 mL N_2_ was injected into the bottle, a 1-mL liquid sample was withdrawn from the bottle and filtered through a 0.22-μm membrane filter immediately. Half of the liquid sample was filtered over a solid phase extraction Na cartridge to remove Fe^2+^ ions. This filtrate was used for the detection of ClO_4_^−^ and its daughter products by an ion chromatograph (IC). The other half of the liquid sample was then acidified with 5 μL H_2_SO_4_ to preserve it until Fe^2+^ determination. Control experiments without Fe^0^ or MoS_2_−NC showed there was no significant loss of ClO_4_^−^ from the sealed bottles (Supplementary Fig. 11), indicating the reduction or adsorption of ClO_4_^−^ by Fe^0^ or MoS_2_−NC can be negligible. Experiments on the effects of oxygen gas were carried out with the solution open to the air. The catalytic stability was tested by cycling tests. MoS_2_−NC was collected on membrane filters (0.22 μm), washed with DO/DI water, and dried at 60 °C in an oven after each run of the experiment. Excess parallel experiments were conducted to compensate for the loss of catalyst and collect enough catalyst for the next run. All other experimental details were the same as those mentioned above.

### Aqueous sample analysis

A Dionex Aquion RFIC IC equipped with an AS16 column and a conductivity detector was utilized to detect ClO_4_^−^ and chlorate. Chlorite and chloride ions were detected with an AS11 column. A KOH eluent generator was used to produce a gradient of KOH solution. Fe^2+^ concentration was determined by 1,10-phenanthroline colorimetric method with an ultraviolet−visible spectrophotometer (Cary 60, Agilent) at 510 nm.

## Supplementary information

Supplementary Information

Transparent Peer Review file

## Source data

Source Data

## Data Availability

The data supporting the findings of this study are included in the paper and its Supplementary Information. Additional data related to this paper are available from the corresponding author upon request. Source data are provided with this paper.
