# Supplementary material for: Donor−acceptor catalysis with defective MoS2 and Fe0 breaks barriers to perchlorate reduction under mild conditions
Source: Nat Commun. 2026 May 20;17:6721. doi: 10.1038/s41467-026-73219-5 (PMC13385889; doi:10.1038/s41467-026-73219-5)
Supplement: Supplementary file 1 — Supplementary Information [file 41467_2026_73219_MOESM1_ESM.pdf]

Supplementary Information for

Donor−acceptor catalysis with defective MoS_2 and Fe^0 breaks barriers to perchlorate reduction under mild conditions

Hejie Qin[1], Han Yang[1], Zhenmin Zhang[1], Yiran Feng[1], Yuankui Sun[1], Peng Fan[2], Zhimin Ao[3], T. David Waite[4], and Xiaohong Guan[1,5]*

[1]Shanghai Engineering Research Center of Biotransformation of Organic Solid Waste, School of Ecological and Environmental Sciences, East China Normal University, Shanghai 200241, PR China

[2] Shanghai Synchrotron Radiation Facility, Shanghai Advanced Research Institute, Chinese Academy of Sciences, Shanghai 201204, P. R. China

[3]Advanced Interdisciplinary Institute of Environment and Ecology, Guangdong Provincial Key Laboratory of Wastewater Information Analysis and Early Warning, Beijing Normal University, Zhuhai 519087, P. R. China

[4]Water Research Centre, School of Civil and Environmental Engineering, University of New South Wales, Sydney, NSW 2052, Australia

[5]Institute of Eco-Chongming, Shanghai 200062, P. R. China

*Corresponding author: Xiaohong Guan

Email: xhguan@des.ecnu.edu.cn

Contents

Method Details S3
Structural models of MoS_2−NC/Fe^0 (**Figures 1**) S6
Density of states (DOS) for the models (**Figures 2**) S7
Charge density difference plot of MoS_2|NC|Fe^0 relative to NC (**Figures 3**) S8
Optimized adsorption configurations of ClO_4^- on the models (**Figure 4**) S9
SEM and TEM images of MoS_2−NC (**Figure 5**) S10
XPS spectra of bulk MoS_2 and MoS_2−NC (**Figure 6**) S11
EXAFS spectra at the Mo *K*-edge of MoS_2−NC (**Figure 7**) S12
Illustration of the ClO_4^- reduction assay (**Figure 8**) S13
TEM images of Fe^0 particles (**Figure 9**) S14
XRD pattern and XPS spectrum of Fe^0 particles (**Figure 10**) S15
ClO_4^- concentration in control experiments (**Figure 11**) S16
Influence of initial concentration of Fe^0 on ClO_4^- reduction (**Figure 12**) S17
Influence of MoS_2−NC dosage on ClO_4^- reduction (**Figure 13**) S18
Influence of Mo loading of MoS_2−NC on ClO_4^- reduction (**Figure 14**) S19
The correlation of Mo−S CNs with k_{Mo} (**Figure 15**) S20
Particle size distributions of Fe^0 samples for particle-size effects (**Figure 16**) S21
Influence of particle size on ClO_4^- reduction (**Figure 17**) S22
Influence of pH on ClO_4^- reduction (**Figure 18**) S23
Influence of pH on zeta potentials of MoS_2−NC and Fe^0. (**Figure 19**) S24
Mo leaching after reaction (**Figure 20**) S25
Release of dissolved Fe^{2+} and Fe^{3+} during ClO_4^- reduction. (**Figure 21**) S26
The distribution of Fe species after reaction (**Figure 22**) S27
Influence of water matrices on ClO_4^- reduction (**Figure 23**) S28
Fe^{2+} release in the presence of NO_3^- and O_2 (**Figure 24**) S29
Schematic diagram of the galvanic cell (**Figure 25**) S30
Influence of S addition and MPS treatment for MoS_2−NC on ClO_4^- reduction (**Figure 26**) S31
Fe^{2+} release in the system of MoS_2−NC with S addition and MPS treatment. (**Figure 27**) S32
EPR spectra for MoS_2−NC and calcined HT-MoS_2−NC (**Figure 28**) S33
ClO_4^- removal in control systems with ball-milled MoS_2 (**Figure 29**) S34
Influence of Mo loading of MoS_2−NC on the Fe^{2+} release (**Figure 30**) S35
EPR spectra for MoS_2−AC and calcined MoS_2−AC+N (**Figure 31**) S36
ClO_4^- reduction using a coarser Fe^0 powder (**Figure 32**) S37
Influence of D_2O on Fe^{2+} release (**Figure 33**) S38
Fitting results of the Mo *K*-edge EXAFS data at *R* space (**Table 1**) S39
Comparison of reduction rate constants in other catalytic systems (**Table 2**) S40
Technoeconomic Analysis (**Note 1**) S41
Supplementary References S46

Method Details

DFT calculations.

Model construction. Structural models included MoS_2 alone (pure MoS_2 slab), MoS_2|NC, MoS_2|Fe^0, and MoS_2|NC|Fe^0 configurations (Supplementary Fig. 1). The MoS_2 slab was modeled as a 4×4 supercell of monolayer 2H-MoS_2, containing 16 Mo atoms and 32 S atoms in its pristine form. Sulfur vacancies were introduced in the top S layer by removing 3 S atoms, yielding 29 S atoms in the slab and creating undercoordinated Mo sites.[1,2] The NC layer was modeled using a 5×5 graphene-based sheet (50 C atoms). Two C atoms were removed to introduce structural vacancies, and five N atoms were doped into the layer by replacing C atoms.[3] The resulting NC model contained 43 C atoms and 5 N atoms, consistent with the XPS results showing the C/N ratio of approximately 8:1. The Fe^0 layer was modeled as a 3×3 Fe(110) slab consisting of 36 Fe atoms.[4] These model sizes (4×4 for MoS_2, 5×5 for NC, and 3×3 for Fe) were selected to enable lateral alignment across layers with minimal lattice mismatch (<10%) after strain optimization. The MoS_2|NC|Fe^0 structure was constructed by vertically stacking the layers in the order of MoS_2, NC, and Fe slabs,[5] with a vacuum region of 14 Å above the MoS_2 surface along the z-axis to avoid periodic interlayer interactions. To represent the aqueous reaction environment, we used the VASPsol implicit solvation model in all DFT calculations,[6-8] therefore, the region above the surface represents a continuum solvent environment rather than a true vacuum. The MoS_2|NC and MoS_2|Fe^0 models were constructed similarly, with MoS_2 directly interfaced with the NC or Fe layer, respectively.

Computational settings. Spin-polarized density functional theory (DFT) calculations were performed using the Vienna Ab-initio Simulation Package (VASP, version 6.2.1) with projector augmented wave (PAW) pseudopotentials.[9] The exchange-correlation effects were treated using the generalized gradient approximation (GGA) with the Perdew-Burke-Ernzerhof (PBE) functional.[10] A plane-wave energy cutoff of 450 eV was applied. The sampling of the Brillioun zone was performed using a Monkhorst-Pack *k*-points grid of 2 × 2 × 1,[1] corresponding to a k-point separation length of 0.05 $Å^{-1}$. Structural optimizations were carried out using the quasi-Newton l-BFGS method until the energy and force converged to less than 1.0×10^{-5} eV and 0.01 eV $Å^{-1}$, respectively. Long-range dispersion interactions were included using Grimme's DFT-D3 correction[11,12] (VASP tag IVDW = 12) to account for noncovalent interactions at the MoS_2−NC, MoS_2−Fe^0, and NC−Fe^0 interfaces. All calculations were spin-polarized (ISPIN = 2) with

MAGMOM initialized for Fe-containing slabs. To consider the solvation of the system, the value of EK_B for VASPsol was 78 to represent the water condition.[13]

Charge analysis and electronic structure. Bader charge analysis was used to quantify charge transfer between layers.[14] To enable comparison across models, the net transferred electrons (Δq, in e^{-}) were further normalized by the interfacial area of the simulation cell (A) in the a-b plane, calculated as $A = a \cdot b \cdot \sin\gamma$ (for the present hexagonal cell with $a = b = 12.41$ Å and $\gamma = 120°$). The area-normalized charge transfer was reported as $\Delta q/A$ (e^{-}/nm^2). Charge density difference plots were calculated using two-fragment decompositions in the same simulation cell and atomic geometry as the corresponding combined system (single-point charge densities). This approach highlights electron redistribution within the donor−acceptor structure. Spin density was obtained from CHGCAR and visualized to confirm the spin distribution. The converged magnetic moments were obtained from OUTCAR. Density of states (DOS) and projected DOS (PDOS) were calculated to evaluate the electronic conductivity and contributions from specific elements near the Fermi level. All visualizations were generated using VESTA. The files of CDD and DOS were generated from VASPKIT.[15]

Processing of free energies. Adsorption energies (E_{ads}) were calculated as:

$$E_{ads} = E_{surface + adsorbate} - E_{surface} - E_{adsorbate}$$

where $E_{surface + adsorbate}$ and $E_{surface}$ are the total energies of the relaxed adsorbate-surface complex and the clean surface, respectively, and $E_{adsorbate}$ is the energy of the isolated adsorbate computed in the same settings.

To simulate aqueous-phase conditions, all adsorption and reaction calculations were performed with the VASP implicit solvation model (VASPsol). For ClO_4^- adsorption on Mo sites, we examined several initial configurations, including adsorption at different undercoordinated Mo sites and different initial orientations of the ClO_4^-. The configuration presented in the manuscript was selected because it converged to a stable adsorbed state and enabled a physically meaningful evaluation of the subsequent reaction steps. For anionic adsorbates (e.g., ClO_4^-), a Na^+ counterion was introduced in the cell to maintain charge neutrality.[16] Bader charge analysis further confirmed that the introduced Na counterion exists essentially as Na^+ (+0.9996 e), while the perchlorate moiety retains an overall negative charge (−0.9092 e), supporting the intended ionic description of Na^+ as the counterion and ClO_4^- as the anionic adsorbate in the charge-compensated calculations.

The Na^+ was initially placed in the solvent-accessible region away from the adsorption site and allowed to relax during geometry optimization.[17,18] In optimized structures, Na^+ did not form direct coordination bonds to the active site and remained solvated (Supplementary Fig. 4). In Fig. 1, the Na^+ was not included for clarity, because it resides far from the surface and may misleadingly suggest the direct involvement in the reaction mechanism.

Reaction energetics were reported using a free-energy approximation. Gibbs free energies at 298 K ($U = 0$ V) were calculated as $G(T) = E_{DFT} + E_{ZPE} + \Delta H(T) - TS(T)$.[19] The ZPE-corrected energy was used as the 0 K enthalpy reference. The ZPE-corrected energy was used as the 0 K enthalpy reference. E_{ZPE}, ΔH, and S values were obtained from vibrational frequency calculations using VASPKIT.[15] The energy barriers for Cl−O bond cleavage from adsorbed ClO_4^- ($*ClO_4 \rightarrow *ClO_3 + *O$) were computed using the climbing image nudged elastic band (CI-NEB) method.[20,21] Subsequent steps including $*O \rightarrow *OH$ hydrogenation and OH^- desorption, were evaluated using free energy changes (ΔG) with ZPE and entropy contributions at 298 K. For the hydrogenation of surface O atom (e.g., $*O + H^+ + e^-+\rightarrow *OH$), the reaction energy change was calculated by $\Delta E = E_{OH*} - E_{O*} - (E_{H^+} + E_{e^-})$, where E_{OH*} is the total energy of the surface with OH, and, according to the reaction of $H^+ + e^- \rightarrow 1/2H_2{}^-$, $E_{H^+} + E_{e^-}$ is estimated from the half the energy of H_2 gas ($1/2E_{H_2} - kT\ln[H+]$).[22] Accordingly, pH effects were included by applying a thermodynamic correction of 0.0592×pH at 298 K per transferred (H^++e^-).[23,24] Unless otherwise noted, pH was set to 6.0 to match experimental conditions.

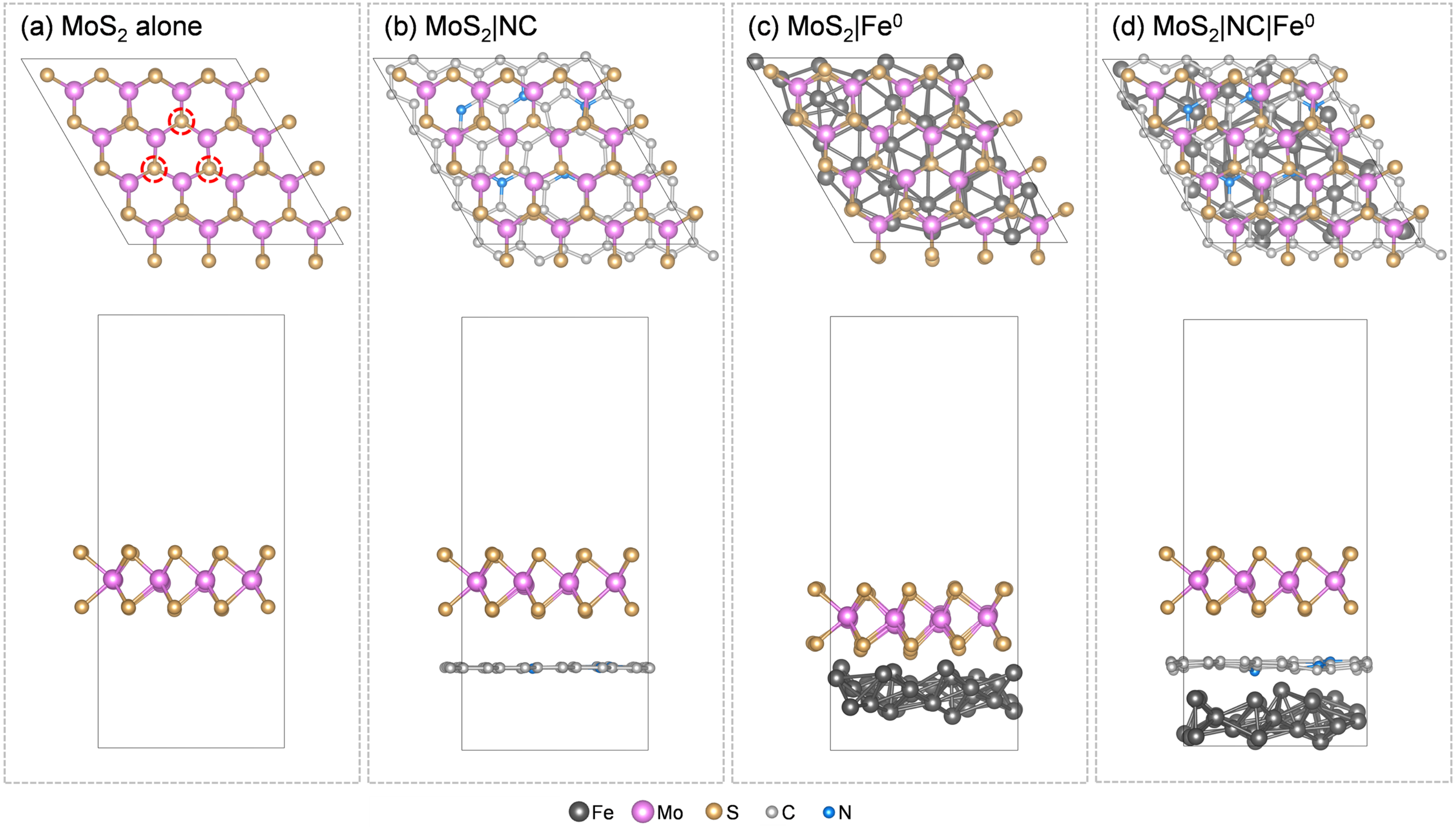

Supplementary Figure 1. Structural models of (a) MoS_2 alone, (b) MoS_2|NC, (c) $MoS_2|Fe^0$, and (d) $MoS_2|NC|Fe^0$. Each panel shows both the top view (upper) and side view (lower) of the optimized structures. Red dashed circles in (a) represent the positions of sulfur vacancies, which are identically introduced in all four models but are not denoted in panels (b-d) for clarity.

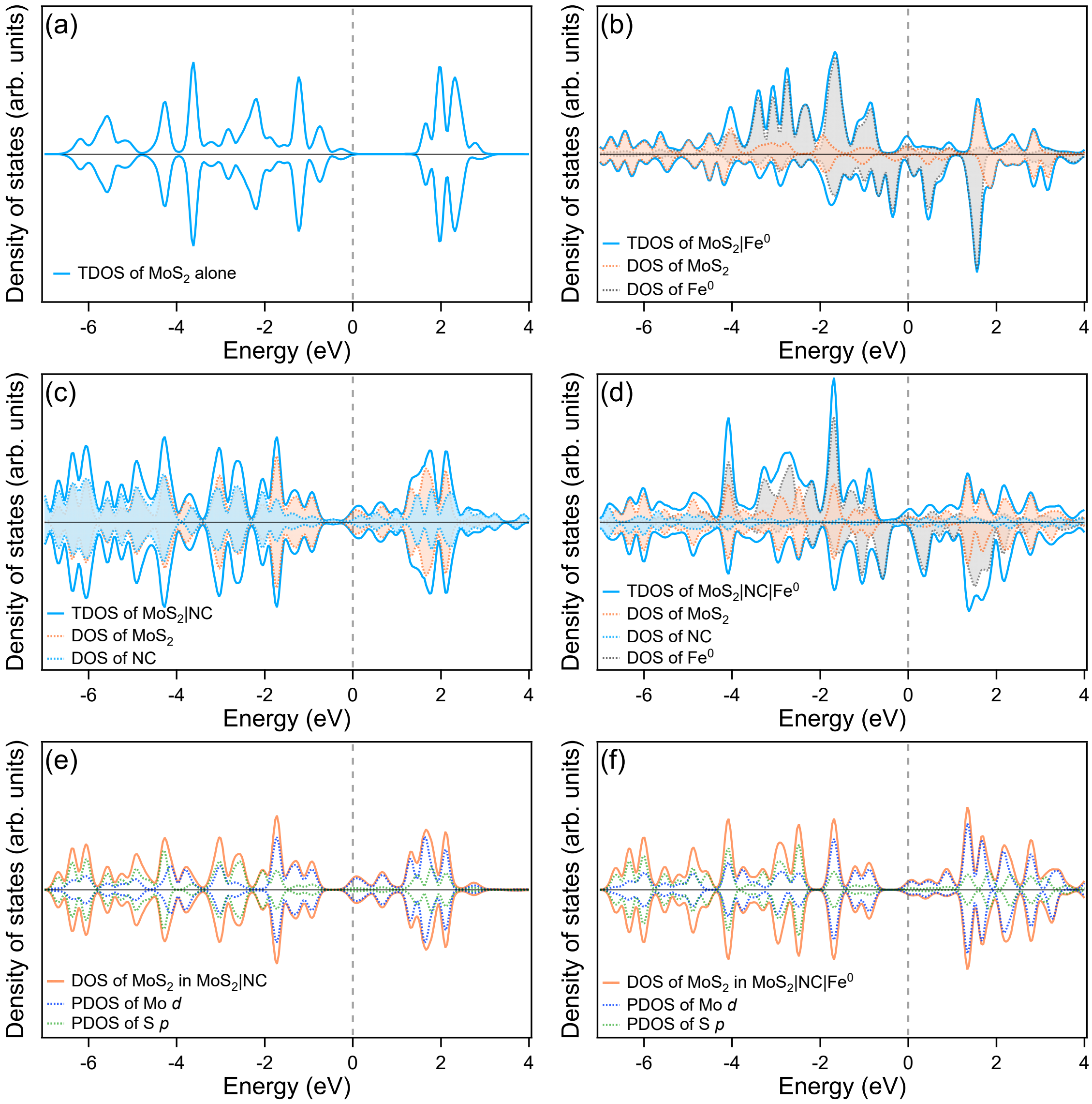

Supplementary Figure 2. Density of states (DOS) for (a) MoS_2 alone, (b) MoS_2|Fe^0, (c) MoS_2|NC, (d) MoS_2|NC|Fe^0, with the layer-projected DOS shown for the contributions from MoS_2, NC, Fe^0 where applicable. (e, f) Projected DOS (PDOS) of MoS_2 in (e) MoS_2|NC and (f) MoS_2|NC|Fe^0, in Mo *d* and S *p* orbits. Compared with MoS_2 alone, electronic states appear near the Fermi level (vertical dashed line) in MoS_2|NC, MoS_2|NC|Fe^0, which is not only contributed by NC, but also due to the MoS_2, indicating electron redistribution between the layers. For MoS_2|NC, MoS_2|NC|Fe^0, Mo 3d PDOS plots shows the near-Fermi states of MoS_2 are dominated by Mo sites. The DOS in panel b and c exhibit asymmetry between spin-up and spin-down channels, due to the spin-polarized electronic structure of metallic Fe^0.

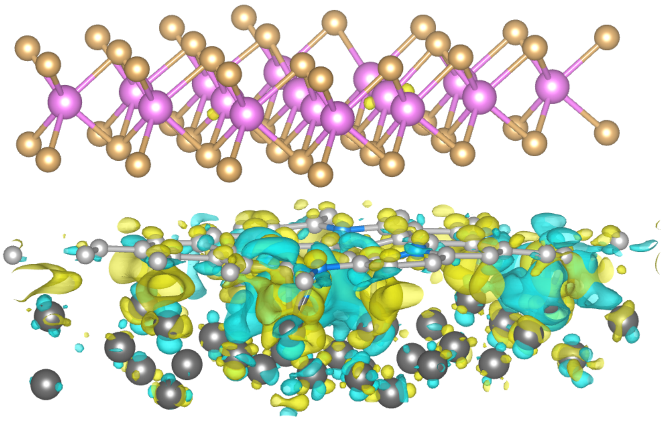

Supplementary Figure 3. Charge density difference (CDD) plot of MoS_2|NC|Fe^0 relative to NC.

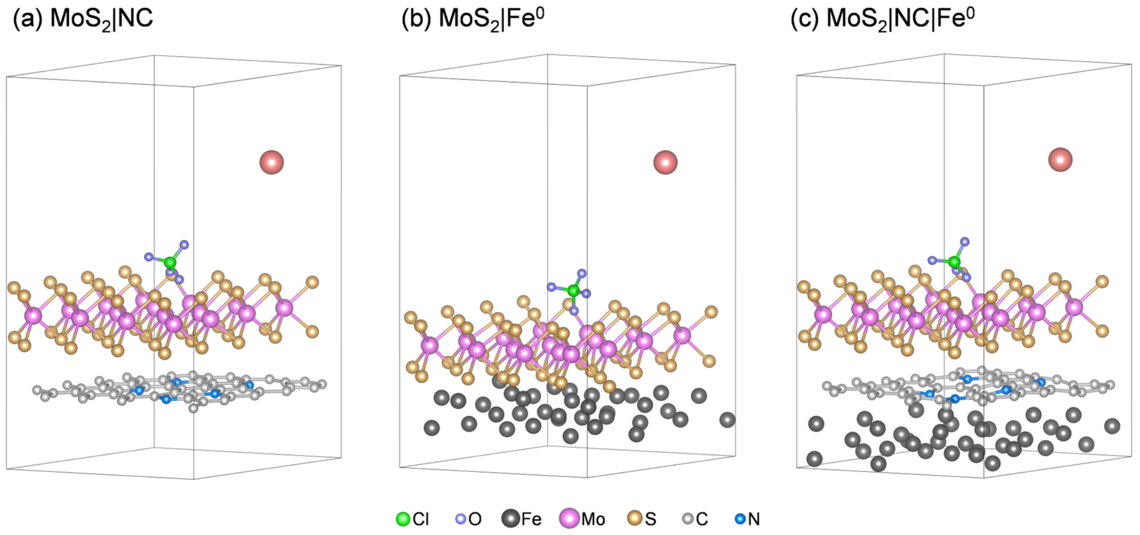

Supplementary Figure 4. Optimized adsorption configurations of ClO_4^- on (a) MoS_2|NC, (b) MoS_2|Fe^0, and (c) MoS_2|NC|Fe^0 models. A Na^+ counterion is included in each simulation cell to maintain overall charge neutrality for the anionic ClO_4^- adsorbate under periodic boundary conditions. The Na^+ ion resides in the solvent-accessible region away from the active site and does not form direct coordination with the adsorbed ClO_4^- or Mo sites after relaxation

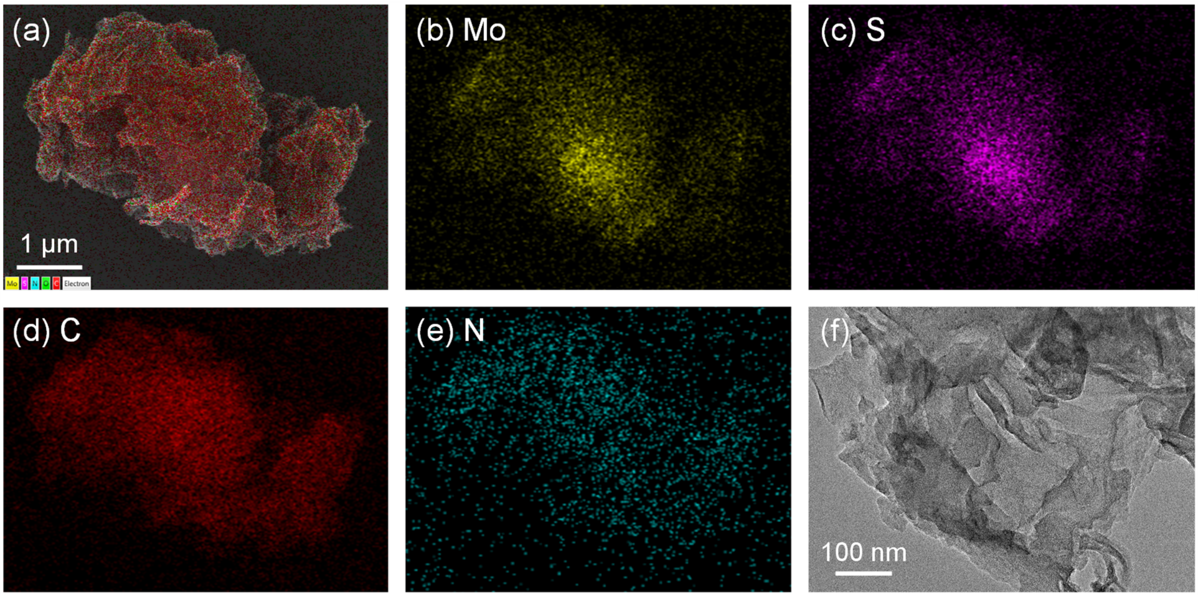

Supplementary Figure 5. (a) SEM image and (b-e) corresponding EDS element mapping of MoS_2−NC (4.2 wt.% Mo): Mo (yellow), S (purple), C (red), N (cyan). (f) TEM image of MoS_2−NC (4.2 wt.% Mo).

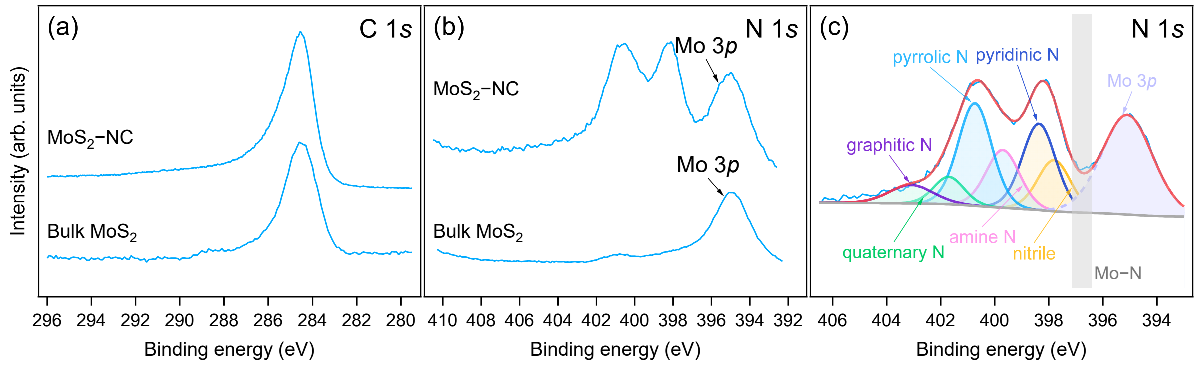

Supplementary Figure 6. XPS spectra of bulk MoS_2 and MoS_2−NC (4.2 wt.% Mo). (a) C 1*s* spectra of bulk MoS_2 and MoS_2−NC. (b) N 1*s* spectra of bulk MoS_2 and MoS_2−NC. (c) The fitting of the N 1*s* spectrum of MoS_2−NC. Panel c shows the several types of N species, including pyridinic N (398.4 eV), pyrrolic N (400.7), amine N (399.7 eV), nitrile (397.8 eV), quaternary N (401.7) and graphitic N (403.1 eV).[25-27] The peak in the right-hand side of panel c is assigned to Mo 3*p* based on panel b. The binding-energy range expected for Mo−N bonds (396.4-397.1) is shaded in panel c,[28,29] where introducing an additional peak could not achieve satisfactory fit.

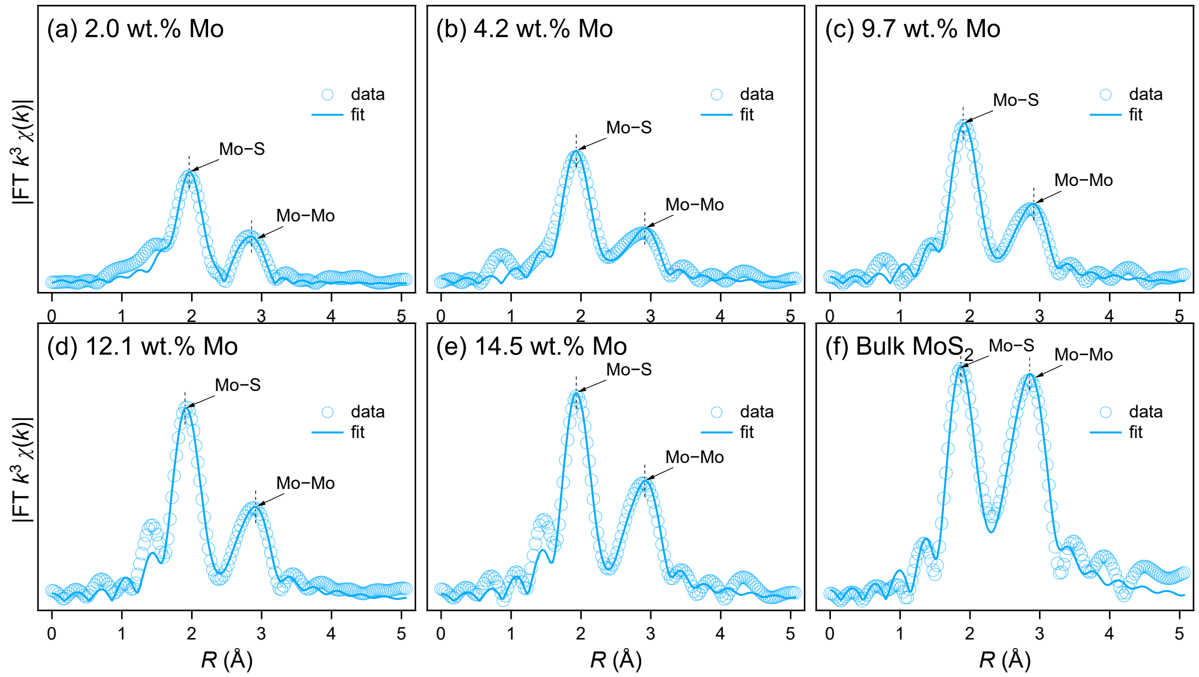

Supplementary Figure 7. Fourier-transformed (FT) k^3-weighted EXAFS spectra at the Mo *K*-edge of MoS_2−NC and bulk MoS_2 at R space.

186 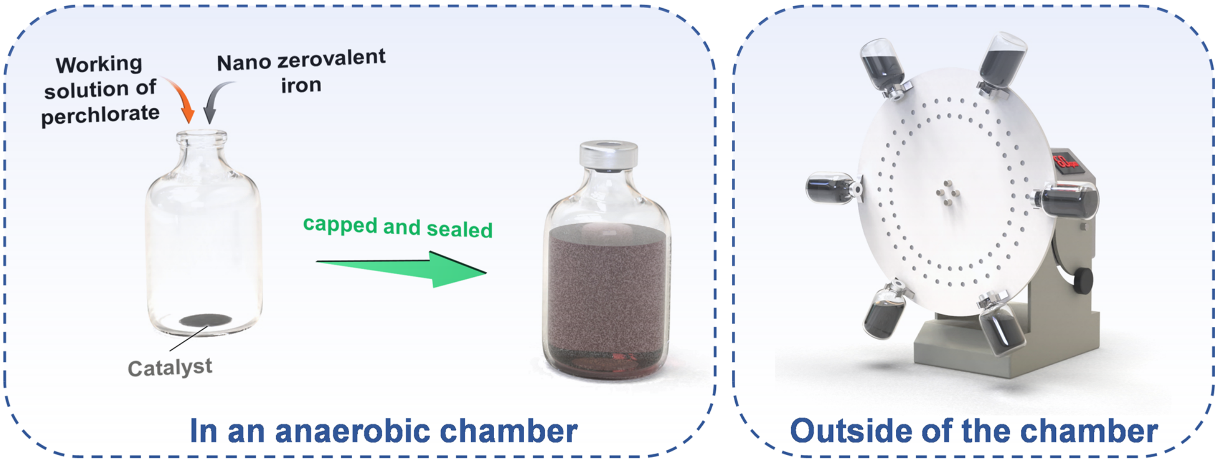

187 **Supplementary Figure 8**. Illustration of the ClO_4^- reduction assay.

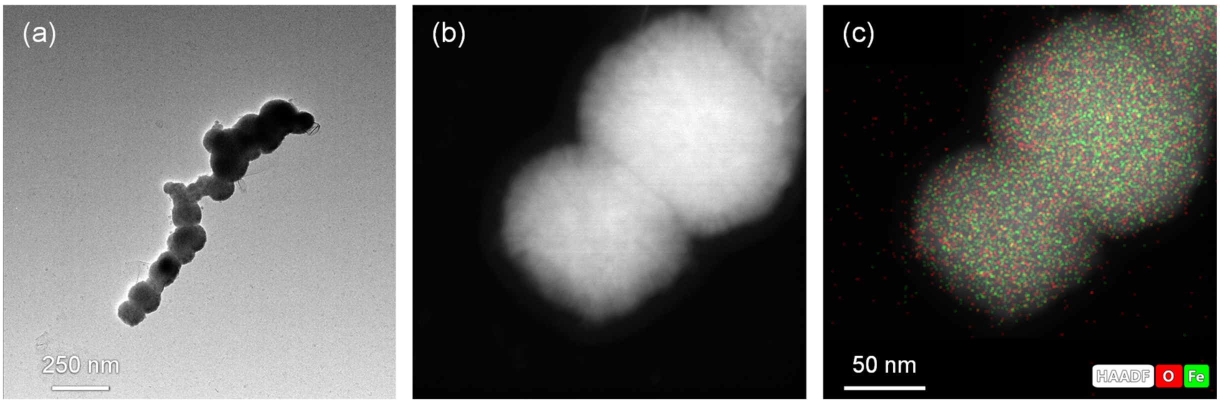

Supplementary Figure 9. (a) Transmission electron microscopy (TEM), (b) high-angle annular dark-field scanning TEM (HAADF-STEM) imaging and corresponding (c) EDS elemental mapping of Fe^0 particles.

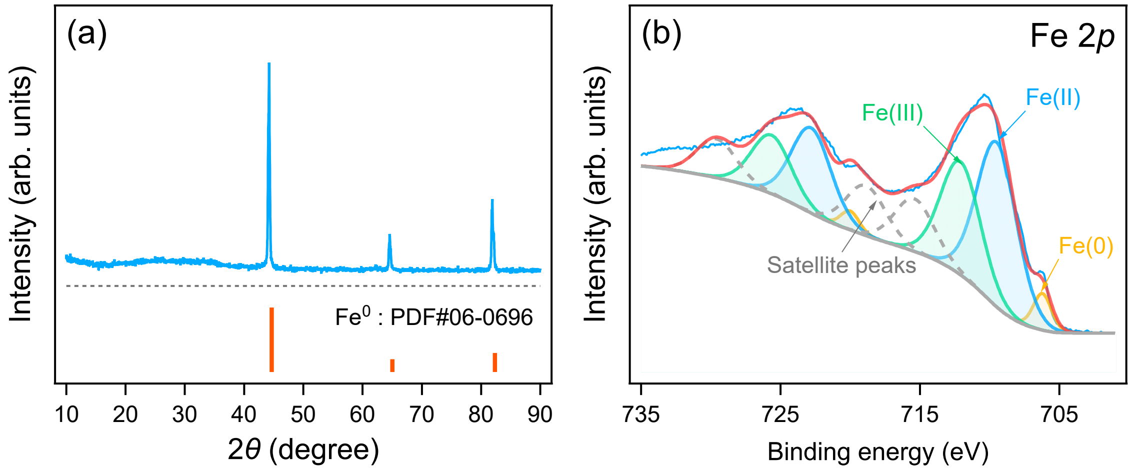

Supplementary Figure 10. (a) X-ray diffraction (XRD) pattern and (b) X-ray photoelectron spectroscopy (XPS) spectrum of Fe^0 particles used in this study.

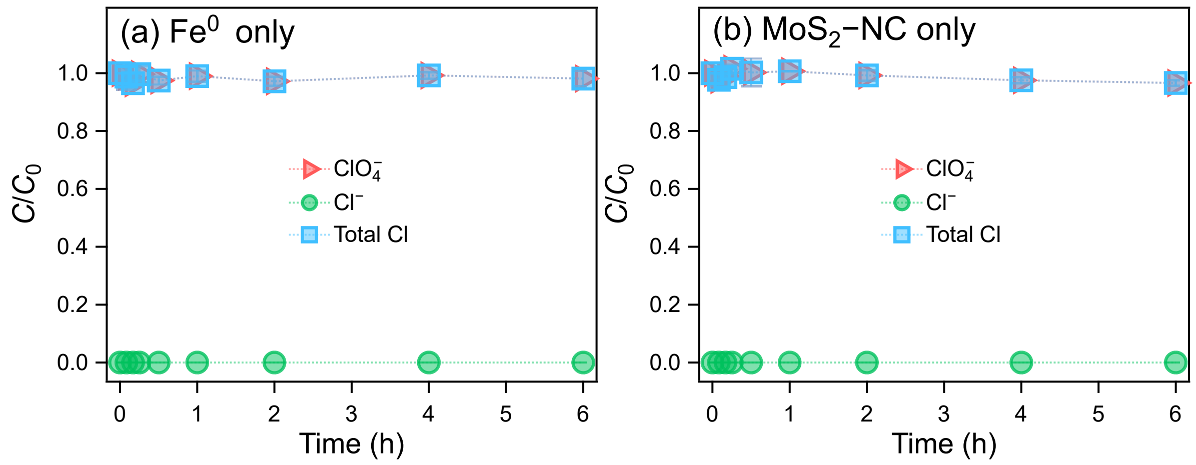

Supplementary Figure 11. ClO_4^- concentration in control experiments: (a) Fe^0 only; (b) MoS_2−NC only. Reaction conditions: $[ClO_4^-]_0$ = 1.0 mM, $[Fe^0]_0$ = 1.2 g/L for (a), [MoS_2−NC] = 4.0 g/L for (b), pH was maintained at 6.0 with 150 mM MES buffer, T = 25 °C. Error bars represent standard deviations from two independent experiments.

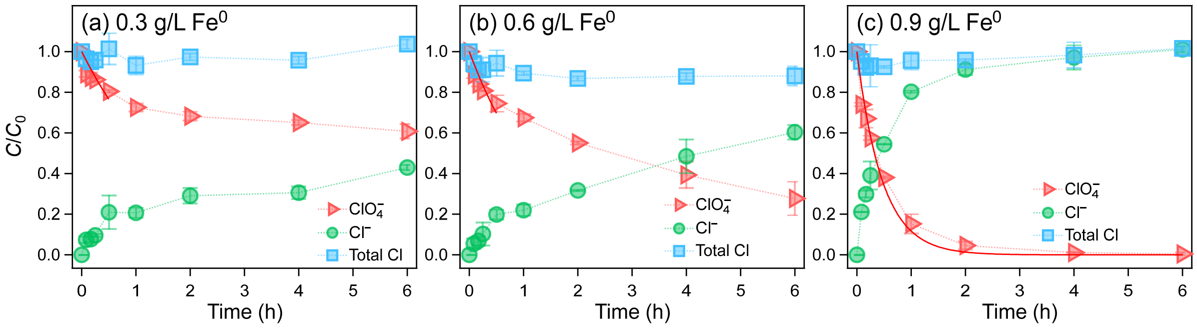

Supplementary Figure 12. Influence of initial concentration of Fe^0 ($[Fe^0]_0$) on (a-c) the ClO_4^- reduction in MoS_2−NC/Fe^0 systems. Data for 1.2 g/L Fe^0 are shown in Fig. 2a in the main text. Reaction conditions: $[ClO_4^-]_0$ = 1.0 mM, $[Fe^0]_0$ = 1.2 g/L, [MoS_2−NC] = 4.0 g/L, the Mo loading is 4.2 wt.%, pH was maintained at 6.0 with 150 mM MES buffer, T = 25 °C. Solid lines in panels a-c are the pseudo-first-order fits. Error bars represent standard deviations from two independent experiments.

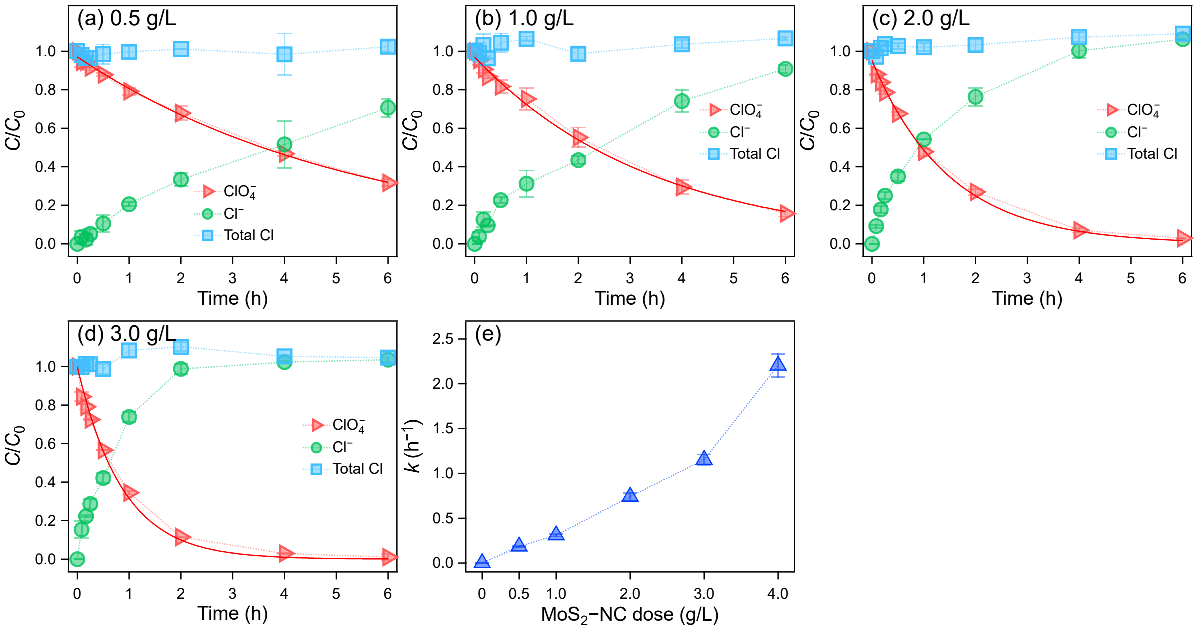

Supplementary Figure 13. Influence of MoS_2−NC dosage on (a-d) the ClO_4^- reduction in MoS_2−NC/Fe^0 systems and (e) the reduction rate constant of ClO_4^-. Data for 4.0 g/L MoS_2−NC are shown in Fig. 2a in the main text. Reaction conditions: $[ClO_4^-]_0$ = 1.0 mM, $[Fe^0]_0$ = 1.2 g/L, [MoS_2−NC] = 4.0 g/L, the Mo loading is 4.2 wt.%, pH was maintained at 6.0 with 150 mM MES buffer, T = 25 °C. Solid lines in panels a-d are the pseudo-first-order fits and the results are shown in panel e. Error bars represent standard deviations from two independent experiments.

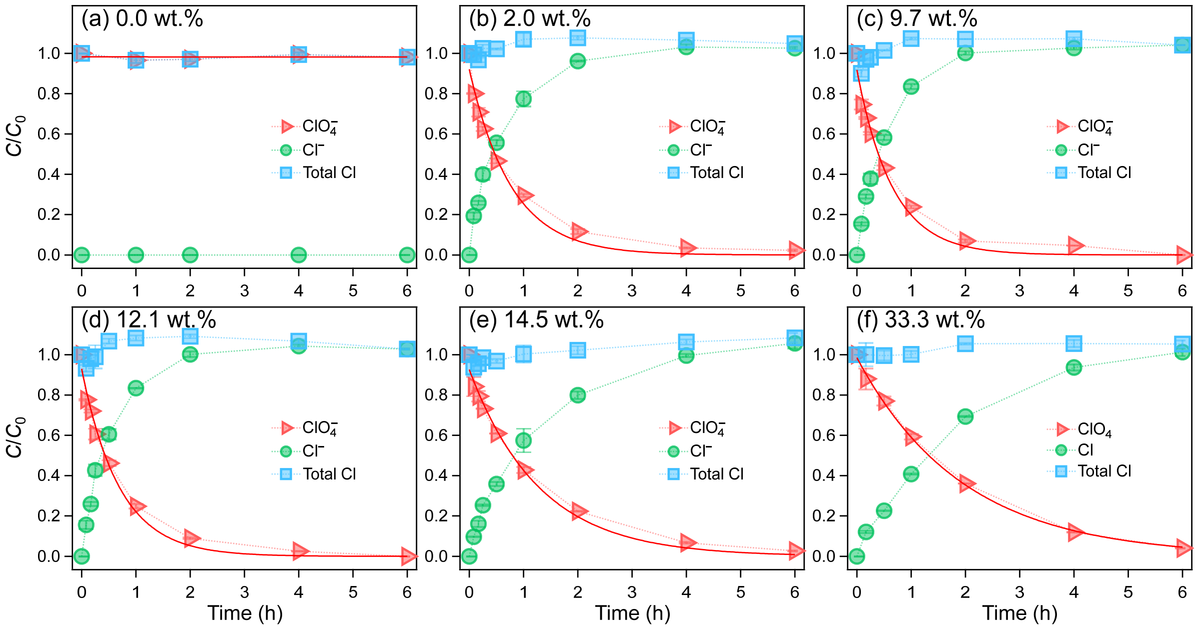

Supplementary Figure 14. Influence of Mo loading of MoS_2−NC on the ClO_4^- reduction in MoS_2−NC/Fe^0 systems. Data for 4.2 wt.% are shown in Fig. 2a in the main text. Reaction conditions: $[ClO_4^-]_0$ = 1.0 mM, $[Fe^0]_0$ = 1.2 g/L, [MoS_2−NC] = 4.0 g/L, pH was maintained at 6.0 with 150 mM MES buffer, T = 25 °C. Solid lines in panels a-e are the pseudo-first-order fits. Error bars represent standard deviations from two independent experiments.

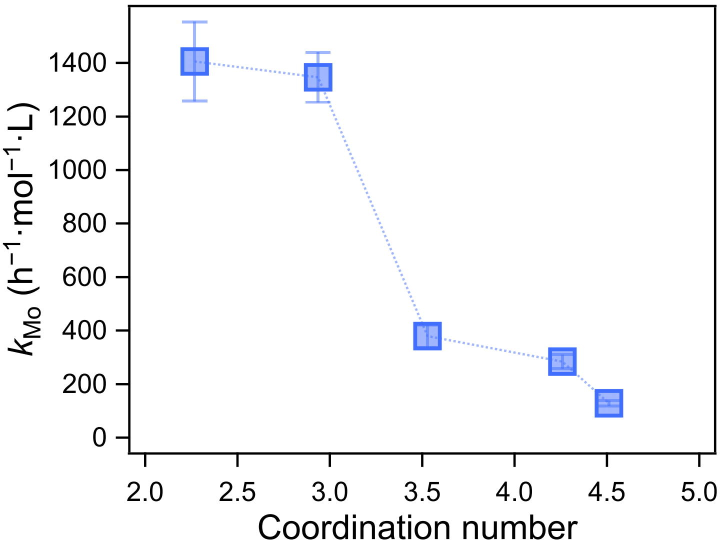

Supplementary Figure 15. The effect of Mo−S coordination number on the rate constants normalized by Mo content (k_{Mo}). Reaction conditions: $[ClO_4^-]_0$ = 1.0 mM, $[Fe^0]_0$ = 1.2 g/L, [MoS_2−NC] = 4.0 g/L, pH was maintained at 6.0 with 150 mM MES buffer, T = 25 °C. Solid lines in panels a-e are the pseudo-first-order fits. Error bars represent standard deviations from two independent experiments.

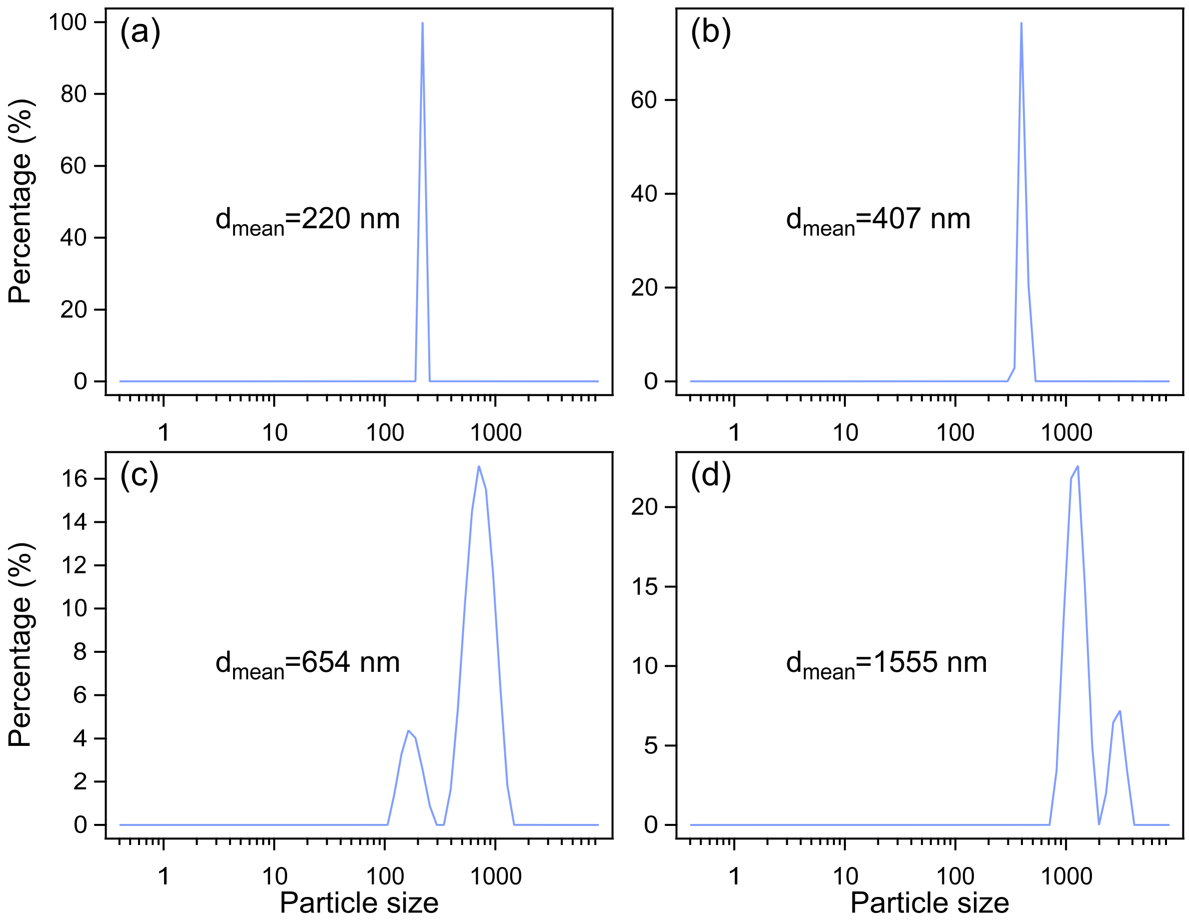

Supplementary Figure 16. Particle size distributions of four Fe^0 samples used to evaluate particle-size effects. In addition to the Fe^0 used in the main experiments (panel a, particle sizes of 220 nm), larger Fe^0 particles with mean sizes of 407 and 654 nm (panels b and c) were synthesized by increasing the concentration of ferrous sulfate to 89.3 mM and 178.6 mM, respectively. A commercial Fe^0 sample (Macklin) with a mean particle size of 1555 nm was also included for comparison (panel d).

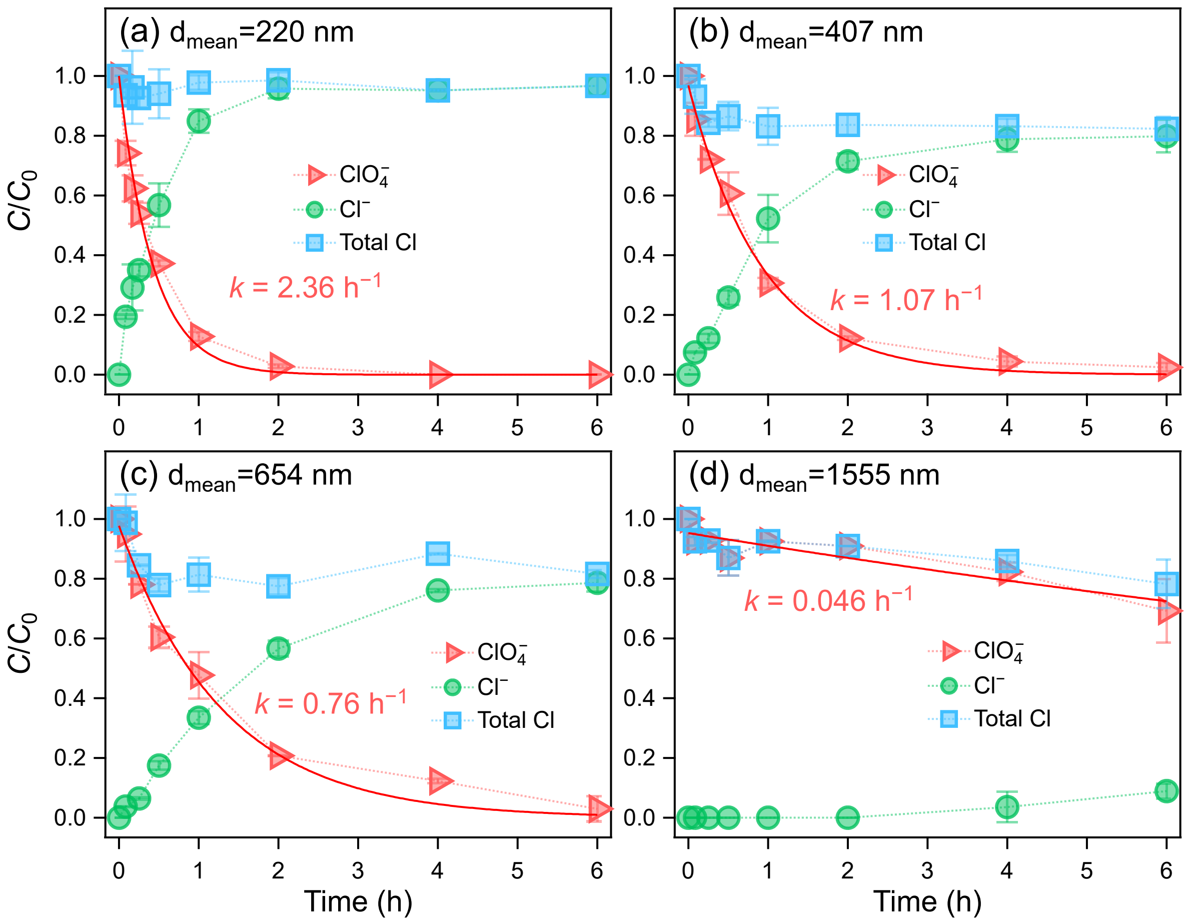

Supplementary Figure 17. Influence of particle sizes of Fe^0 on the ClO_4^- reduction in MoS_2−NC/Fe^0 systems. Data for 220 nm are those shown in Fig. 2a in the main text, and presented here for comparison. Reaction conditions: $[ClO_4^-]_0$ = 1.0 mM, $[Fe^0]_0$ = 1.2 g/L, [MoS_2−NC] = 4.0 g/L, the Mo loading is 4.2 wt.%, pH was maintained at 6.0 with 150 mM buffer, T = 25 °C. Solid lines in panels a-d are the pseudo-first-order fits. Error bars represent standard deviations from two independent experiments.

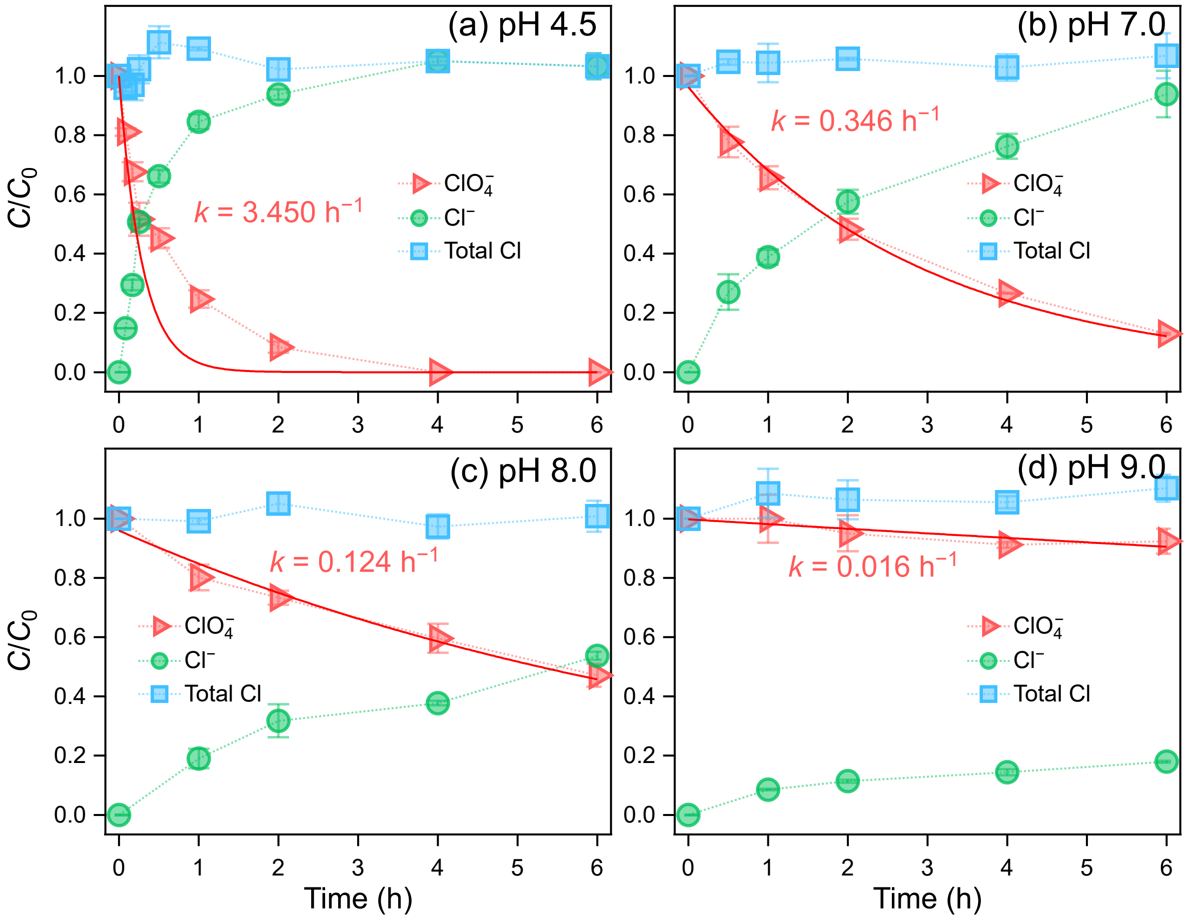

Supplementary Figure 18. Influence of pH on the ClO_4^- reduction in MoS_2−NC/Fe^0 systems. Data for pH 6.0 are shown in Fig. 2a in the main text. Reaction conditions: $[ClO_4^-]_0$ = 1.0 mM, $[Fe^0]_0$ = 1.2 g/L, [MoS_2−NC] = 4.0 g/L, the Mo loading is 4.2 wt.%, pH was maintained with 150 mM buffer, T = 25 °C. Solid lines in panels a-d are the pseudo-first-order fits. Error bars represent standard deviations from two independent experiments.

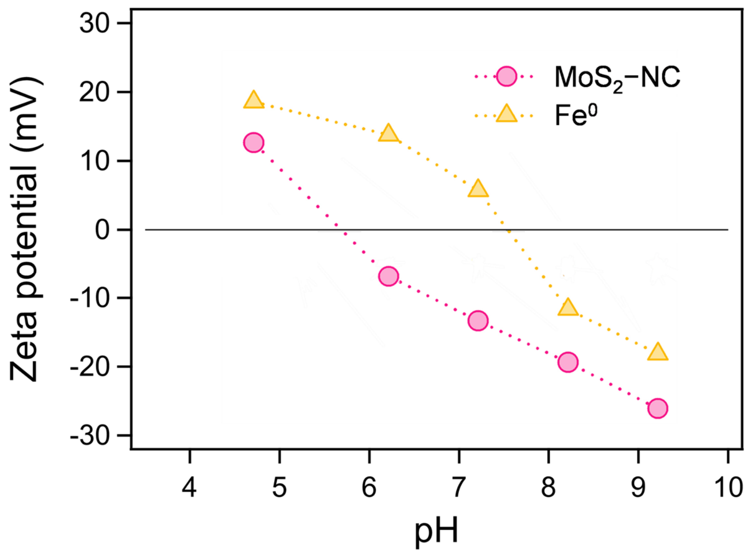

Supplementary Figure 19. Influence of pH on zeta potentials of MoS_2−NC and Fe^0.

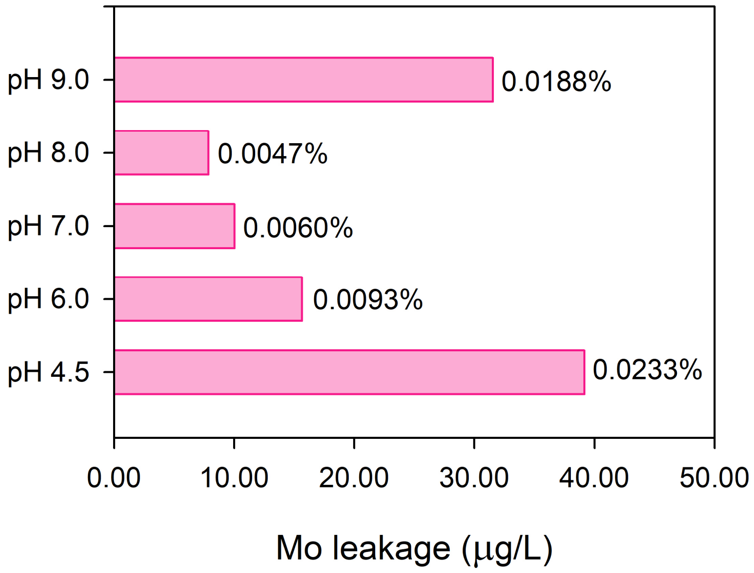

Supplementary Figure 20. Mo leaching after reaction. Reaction conditions: $[ClO_4^-]_0$ = 1.0 mM, $[Fe^0]_0$ = 1.2 g/L, $[MoS_2\text{−}NC]$ = 4.0 g/L, the Mo loading is 4.2 wt.%, pH was maintained with 150 mM buffer, T = 25 °C.

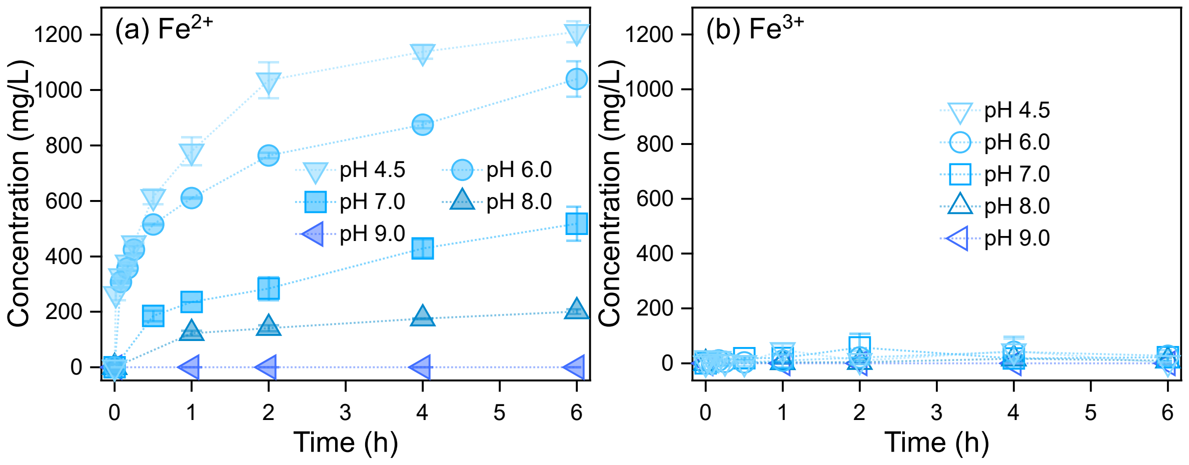

Supplementary Figure 21. Release of dissolved (a) Fe^{2+} and (b) Fe^{3+} during ClO_4^- reduction. Reaction conditions: $[ClO_4^-]_0$ = 1.0 mM, $[Fe^0]_0$ = 1.2 g/L, [MoS_2−NC] = 4.0 g/L, the Mo loading is 4.2 wt.%, pH was maintained with 150 mM buffer, T = 25 °C. Error bars represent standard deviations from two independent experiments.

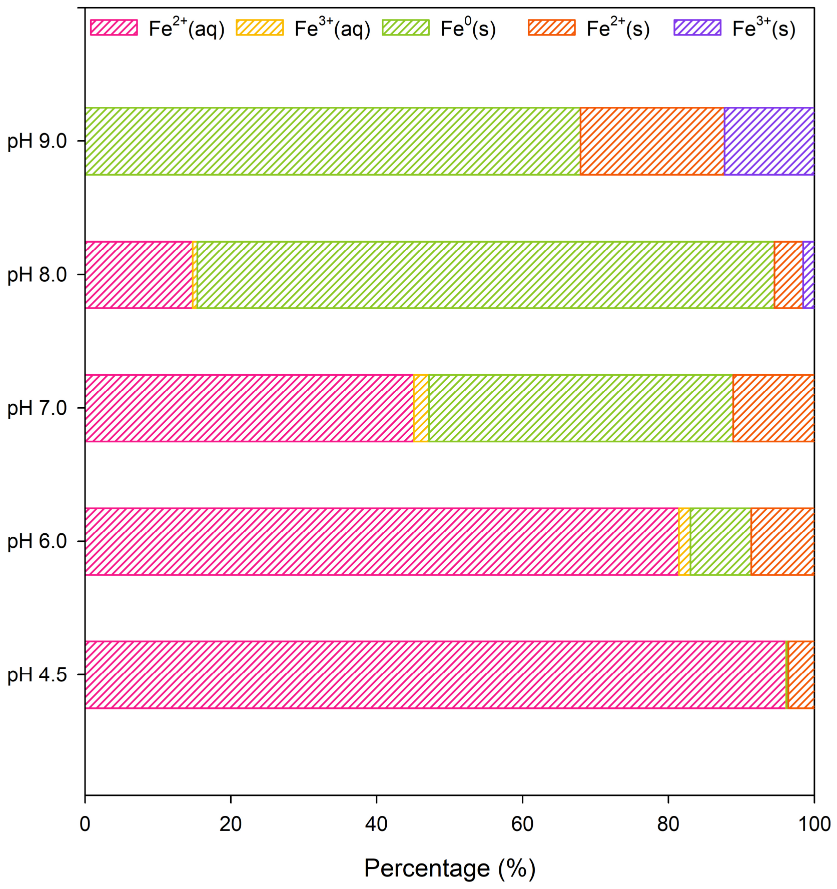

Supplementary Figure 22. The distribution of Fe species after reaction. Reaction conditions: $[ClO_4^-]_0$ = 1.0 mM, $[Fe^0]_0$ = 1.2 g/L, [MoS_2−NC] = 4.0 g/L, the Mo loading is 4.2 wt.%, pH was maintained with 150 mM buffer, T = 25 °C.

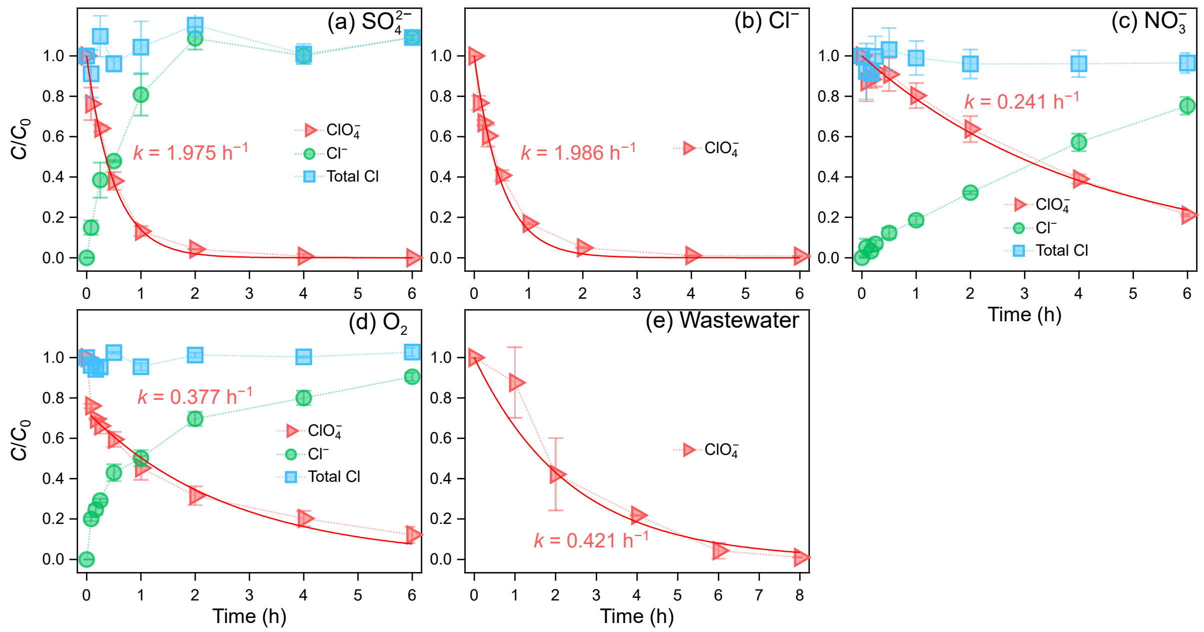

Supplementary Figure 23. Influence of water matrices on the ClO_4^- reduction in MoS_2−NC/Fe^0 systems: (a) 10 mM SO_4^{2-}, (b) 10 mM Cl^-, (c) 2 mM NO_3^-, (d) O_2 (under aerobic condition), (e) in real wastewater (under aerobic condition). Reaction conditions: $[ClO_4^-]_0$ = 1.0 mM for (a-d), $[ClO_4^-]_0$ = 2.1 mM for (e), $[Fe^0]_0$ = 1.2 g/L, [MoS_2−NC] = 4.0 g/L, the Mo loading is 4.2 wt. Solid lines are the pseudo-first-order fits. Error bars represent standard deviations from two independent experiments.

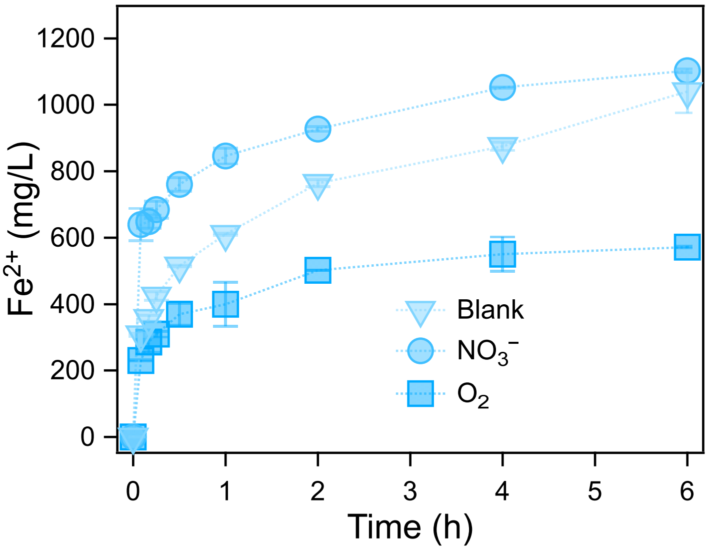

Supplementary Figure 24. Release of dissolved Fe^{2+} in the presence of NO_3^- and O_2. Reaction conditions: $[ClO_4^-]_0$ = 1.0 mM, $[Fe^0]_0$ = 1.2 g/L, [MoS_2−NC] = 4.0 g/L, the Mo loading is 4.2 wt.%, $[NO_3^-]$ = 2 mM, pH was maintained at 6.0 with 150 mM MES buffer, T = 25 °C. Error bars represent standard deviations from two independent experiments.

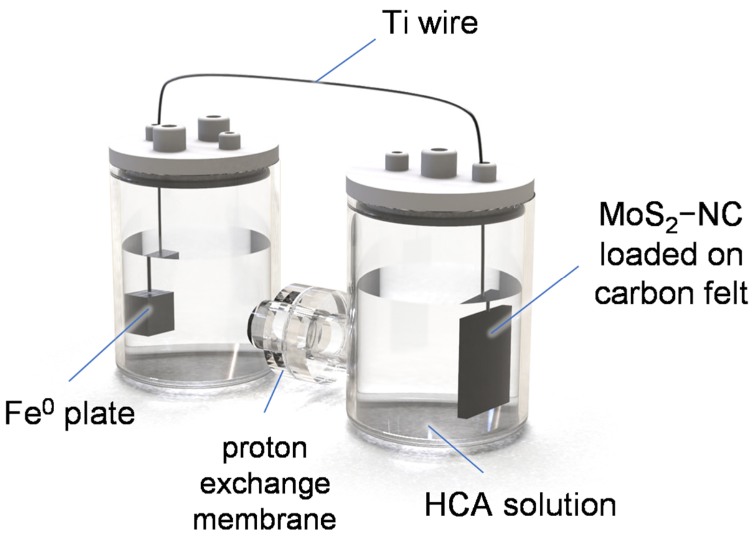

Supplementary Figure 25. Schematic diagram of the galvanic cell. Reaction conditions: $[ClO_4^-]_0$ = 1.0 mM, $[MoS_2{-}NC]$ = 4.0 g/L, the Mo loading is 4.2 wt.%, pH was maintained at 6.0 with 150 mM MES buffer, T = 25 °C.

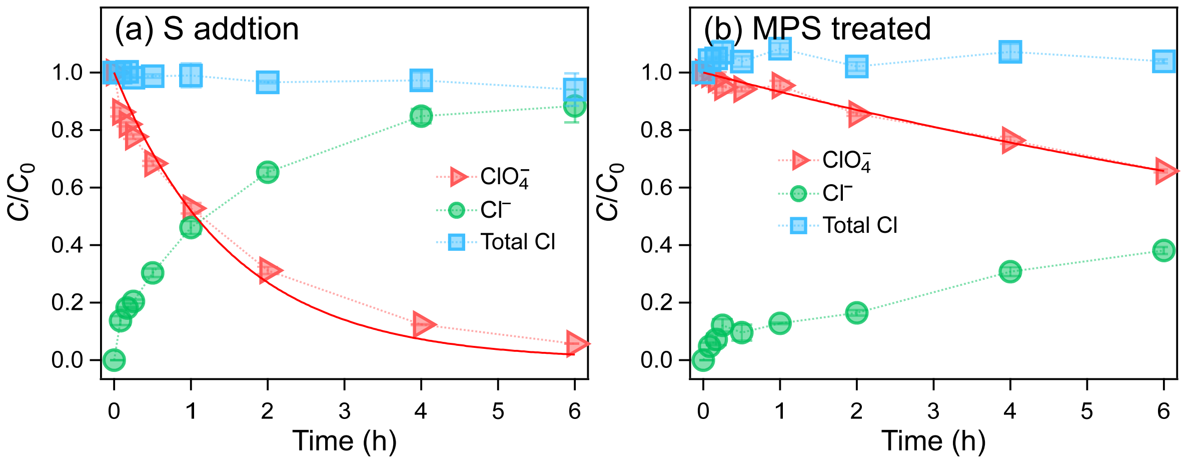

Supplementary Figure 26. Influence of S addition and MPS treatment for MoS_2−NC on the ClO_4^- reduction. Reaction conditions: $[ClO_4^-]_0$ = 1.0 mM, $[Fe^0]_0$ = 1.2 g/L, [MoS_2−NC] = 4.0 g/L, the Mo loading is 4.2 wt.%, pH was maintained at 6.0 with 150 mM MES buffer, T = 25 °C. Error bars represent standard deviations from two independent experiments.

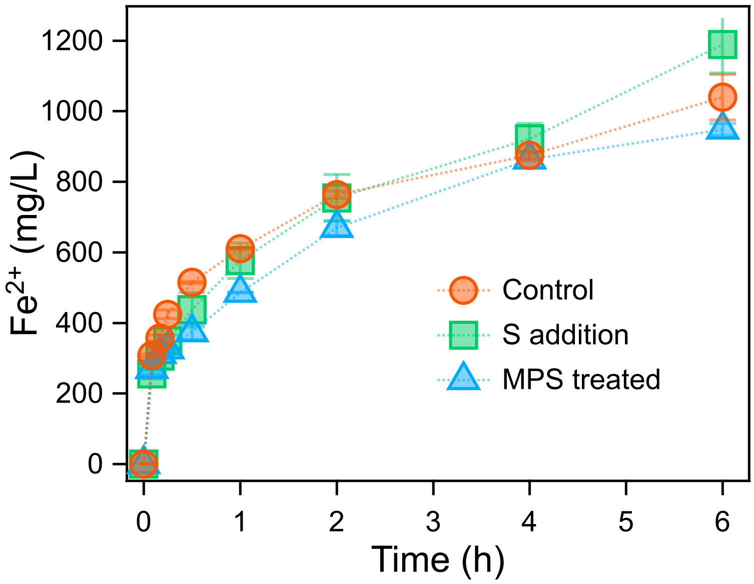

Supplementary Figure 27. Release of dissolved Fe^{2+} in the system of MoS_2−NC with S addition and MPS treatment. Reaction conditions: $[ClO_4^-]_0$ = 1.0 mM, $[Fe^0]_0$ = 1.2 g/L, $[MoS_2$−NC] = 4.0 g/L, the Mo loading is 4.2 wt.%, pH was maintained at 6.0 with 150 mM MES buffer, T = 25 °C. Error bars represent standard deviations from two independent experiments.

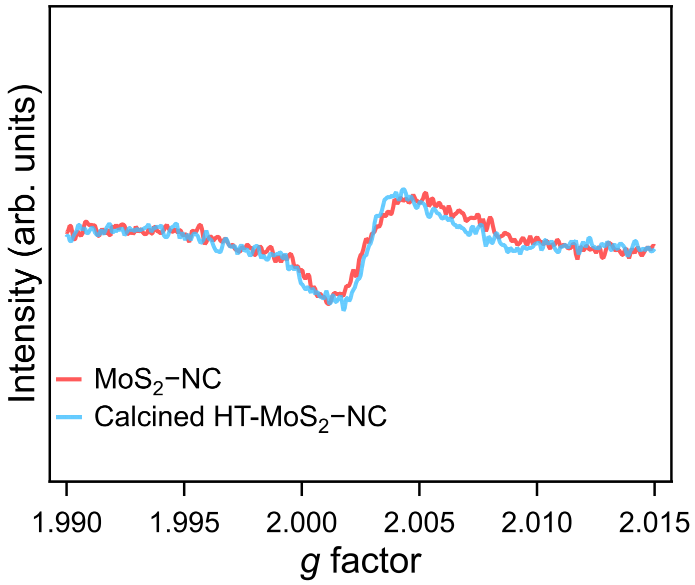

Supplementary Figure 28. The electron paramagnetic resonance (EPR) spectra for MoS_2−NC and calcined HT-MoS_2−NC.

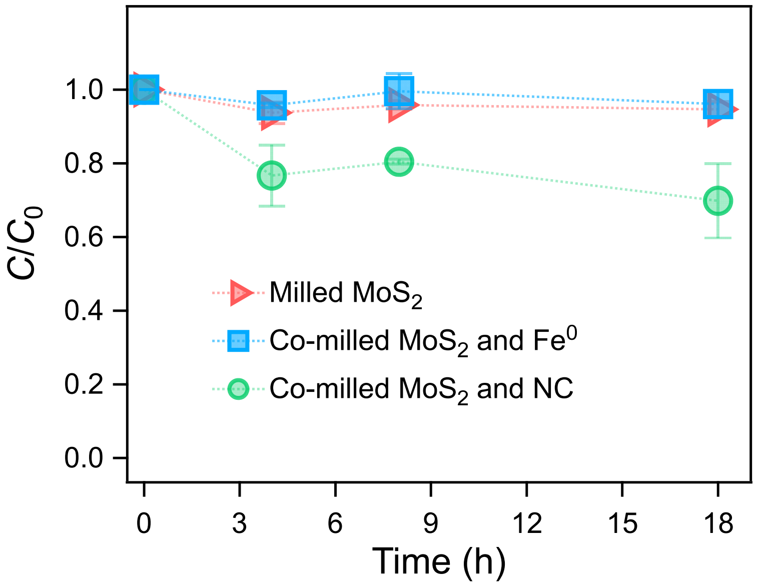

Supplementary Figure 29. ClO_4^- removal in three control systems, including ball-milled MoS_2 with Fe^0 added during reaction, co-milled MoS_2 and Fe^0 (no additional Fe^0 during reaction), and co-milled MoS_2 and NC, with Fe^0 added during reaction. Reaction conditions: $[ClO_4^-]_0$ = 1.0 mM, $[Fe^0]_0$ = 1.2 g/L, $[MoS_2]$ = 0.28 g/L, [NC] = 3.72 g/L, all the concentrations refer to the final solution, and are identical to those in Fig. 3a. pH is maintained at 6.0 with 150 mM buffer, T = 25 °C. Error bars represent standard deviations from two independent experiments.

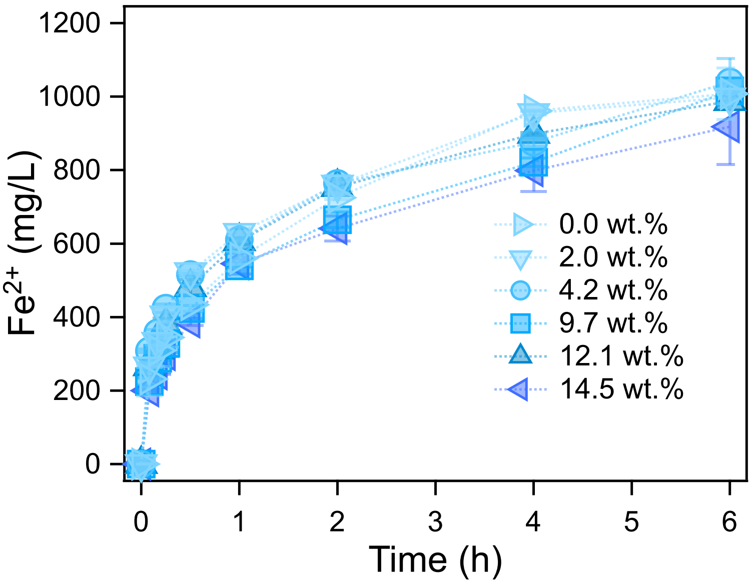

Supplementary Figure 30. Influence of Mo loading of MoS_2−NC on the Fe^{2+} release in MoS_2−NC/Fe^0 systems. Reaction conditions: $[ClO_4^-]_0$ = 1.0 mM, $[Fe^0]_0$ = 1.2 g/L, [MoS_2−NC] = 4.0 g/L, pH was maintained at 6.0 with 150 mM MES buffer, T = 25 °C. Error bars represent standard deviations from two independent experiments.

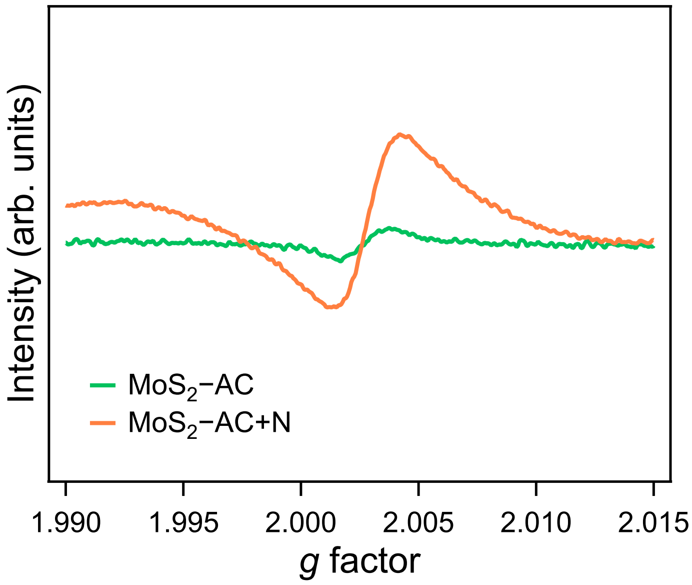

Supplementary Figure 31. The EPR spectra for MoS_2−AC and MoS_2−AC+N.

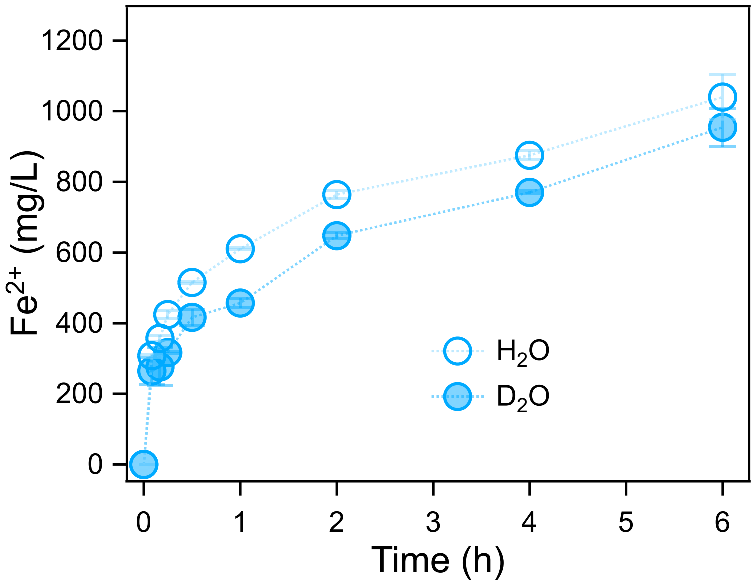

Supplementary Figure 32. Influence of D_2O on Fe^{2+} release in MoS_2−NC/Fe^0 systems. Reaction conditions: $[ClO_4^-]_0$ = 1.0 mM, $[Fe^0]_0$ = 1.2 g/L, [MoS_2−NC] = 4.0 g/L, pH and pD were maintained at 6.0 with 150 mM MES buffer, T = 25 °C. Error bars represent standard deviations from two independent experiments.

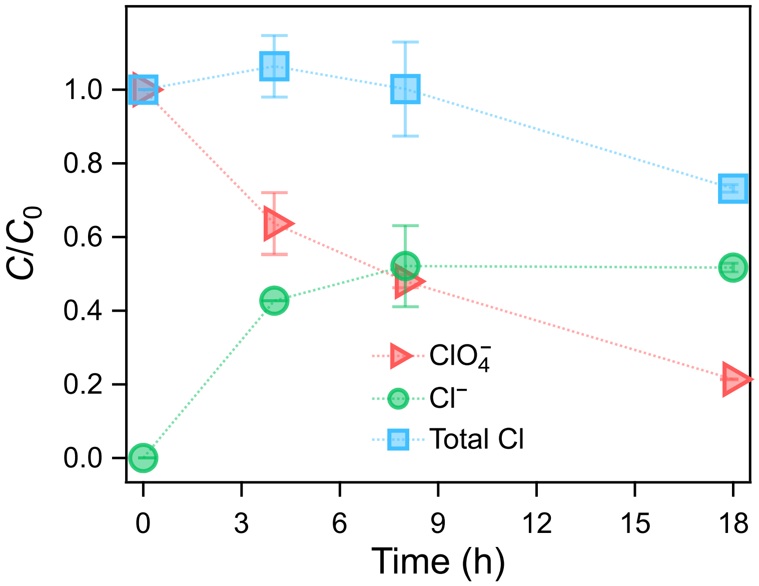

Supplementary Figure 33. ClO_4^- reduction kinetics using a coarser Fe^0 powder (20 mesh, purchased from Thermo Fisher Scientific). Reaction conditions: $[ClO_4^-]_0$ = 1.0 mM, $[Fe^0]_0$ = 12 g/L, [MoS_2−NC] = 4.0 g/L, the Mo loading is 4.2 wt.%, pH was maintained at 6.0 with 150 mM buffer, T = 25 °C. Error bars represent standard deviations from two independent experiments.

Supplementary Table 1. Fitting results of the Mo *K*-edge EXAFS data at *R* space.

Sample	Path	CN	*R* (Å)	σ^2 (Å)	ΔE_0 (eV)	R-factor
MoS_2–NC (2.0 wt.% Mo)	Mo–S	2.269	2.40	0.0030	0.673	0.0427
	Mo–Mo	1.473	3.16	0.0055		
MoS_2–NC (4.2 wt.% Mo)	Mo–S	2.937	2.40	0.0030	2.278	0.0368
	Mo–Mo	1.978	3.16	0.0047		
MoS_2–NC (9.7 wt.% Mo)	Mo–S	3.529	2.40	0.0030	1.365	0.0111
	Mo–Mo	2.500	3.16	0.0041		
MoS_2–NC (12.1 wt.% Mo)	Mo–S	4.259	2.40	0.0030	1.158	0.0250
	Mo–Mo	2.699	3.16	0.0036		
MoS_2–NC (14.5 wt.% Mo)	Mo–S	4.511	2.41	0.0030	3.740	0.0209
	Mo–Mo	3.287	3.17	0.0034		
Bulk MoS_2	Mo–S	6.187	2.40	0.0040	0.206	0.0112
	Mo–Mo	6.083	3.16	0.0028		
MoS_2–NC (4.2 wt.% Mo, during reaction with Fe^0)	Mo–S	2.918	2.39	0.0030	0.789	0.0284
	Mo–Mo	2.004	3.15	0.0040		
MoS_2–NC (4.2 wt.% Mo, during reaction with Fe^0 and ClO_4^-)	Mo–O	0.909	2.06	0.0030	0.791	0.0590
	Mo–S	2.991	2.41	0.0030		
	Mo–Mo	1.943	3.16	0.0047		
Mo foil	Mo–Mo	8.000[a]	2.74	0.0043	6.470	0.0166
	Mo–Mo	6.000[a]	3.17	0.0043		

[a]This value was fixed during fitting according to the known structure of Mo foil.

Note: The amplitude reduction factor was set to 0.998 based on the fitting result for Mo foil. CN is the coordination number; *R* is the distance between central atoms and surrounding coordination atoms; σ^2 is Debye-Waller factor to describe the variance due to thermal and static disorder; ΔE_0 is the inner potential correction.

Supplementary Table 2. Comparison of reduction rate constants in different representative catalytic systems.

Catalyst/reductant	pH	k_{obs} (h^{-1})	Removal	Ref.
MoS_2–NC; Fe^0	4.5	3.46	100% of 1 mM ClO_4^- (2 h)	This work
	6.0	2.35	100% of 1 mM ClO_4^- (4 h)	
	7.0	0.35	87% of 1 mM ClO_4^- (6 h)	
	8.0	0.12	52% of 1 mM ClO_4^- (6 h)	
$[(NH_2)_2bpy]MoO_x$–Pd/C; H_2	3	2.8	100% of 1 mM ClO_4^- (1h)	30,31
	4.7	0.21	83% of 1 mM ClO_4^- (1h)	
Re (hoz)$_2$–Pd/C; H_2	3	2.49	100% of 1 mM ClO_4^- (2h)	32
	5	0.06	-	
Re(O)(hoz)$_2$Cl–Pd/C; H_2	3.8	0.316	100% of 2 mM ClO_4^- (24 h)	33
	5.9	0.0117	~100% of 2 mM ClO_4^- (18 d)	
Pd/Pt–NACF; Electrocatalytic system	5	0.23	81% of 10 mg/L ClO_4^- (8h)	34
Pt–Ti; Electrocatalytic system	5.6	0.22	88% of 1 mg/L ClO_4^- (9h)	35
Rh_xCu/Ti; Electrocatalytic system	3	0.900	99% of 50 mg/L ClO_4^- (6h)	36
	5	0.157		
	7.5	0.06		
Re/Ti_4O_7; Electrocatalytic system	3	-	67.3 % of 5 mM ClO_4^- (in permeate low)	37
	7	-	53.6% of 5 mM ClO_4^- (in permeate low)	
Ti(III) + β-alanine	2.3 (T=50 °C)	-	~93% (1.5 h) of 1 mM ClO_4^- (1.5 h)	38
Ti(II)	$[H^+]_0$=420 mM	0.18	-	39
Fe^0 (CMC-nZVI)	pH_0=6.5 (T=110 °C)	0.984	100% of 10 mg/L ClO_4^- (2 h)	40

Supplementary Note 1: Technoeconomic Analysis

Cost Assumptions for Catalysts and Reagents

Based on the experimental results using coarser Fe^0 powder, we conducted TEA based on realistic commercial prices and industrial prices for both MoS_2–NC/Fe^0 and Pd/H_2-based system.

A preliminary consumables-based cost comparison was performed on a 1 L treatment basis with an initial ClO_4^- concentration of 1 mM. For the MoS_2–NC/Fe^0 system, 4 g/L of MoS_2–NC (4.2 wt.% Mo) and 12 g/L of coarser Fe^0 were applied. Under these conditions, 12 g/L Fe^0 removed 80% of 1 mM ClO_4^- in 18 h; thus, the Fe^0 cost was normalized by 1/0.8 to enable comparison on an equivalent (per 1 mM removed) basis. Using industrial bulk prices for coarser Fe^0 of \$4.4-\$10.22 per kg (from iron-filing.com, chemicalstore.com, and ScienceKitStore.com), the normalized Fe^0 cost was \$0.066-\$0.153 per L. The Mo precursor for MoS_2–NC synthesis, ammonium tetrathiomolybdate, was priced at \$18-\$33 per kg (from scrapmonster.com), contributing \$0.0082–\$0.0150 per L based on the Mo loading (4.2 wt.% Mo). The NC fraction in MoS_2–NC was estimated as ~93 wt.% (3.72 g/L). The base carbon price was taken as \$1.77-\$2.54 per kg using industrial activated carbon and carbon black references (from businessanalytiq.com, indexbox.io, businessanalytiq.com, and imarcgroup.com), and the NC price was estimated by applying a 1.5-6.5× multiplier to account for nitrogen doping and the associated yield penalty, processing energy, etc., following published cost-estimation approaches for functionalized carbons,[41,42] giving an NC price of \$2.66-\$16.51 per kg. This corresponds to an NC cost contribution of \$0.0099-\$0.061 per L. Therefore, the total cost of the MoS_2–NC/Fe^0 system is \$0.084-\$0.229 per L, i.e., 21-87× lower than the Pd/H_2 comparator under the same removal basis.

For the Pd/H_2-based system, we estimated the cost of the $[(NH_2)_2\textit{bpy}]MoO_x$–Pd/C system, where 0.2 g/L catalyst (5 wt.% Pd, 5 wt.% Mo) achieved complete removal of 1 mM ClO_4^-. Using bulk-pack supplier catalog prices for 5 wt.% Pd/C, the catalyst cost was estimated as \$11,450-\$17,840 per kg (from strem.com, fuelcellearth.com, srlchem.com), corresponding to \$2.30-\$3.57 per L at 0.2 g/L. The Mo precursor $Na_2MoO_4 \cdot 2H_2O$ was priced at \$13-\$15 per kg (from made-in-china.com and contributing negligibly (\$0.00033-\$0.00038 per L). The ligand $(NH_2)_2\textit{bpy}$ (assumed 1:1 molar with Mo) was priced at \$125.8-\$189.4 per g (from chemicalbook.com, biosynth.com, and tcichemicals.com), contributing \$2.44-\$3.68 per L. Therefore, the consumables cost for the Pd/H_2 system was \$4.74-\$7.25 per L without operational costs. In addition, Pd/H_2-

based operation typically requires acidification to pH 3 and subsequent neutralization (estimated ~\$0.1 per L depending on alkalinity) and a continuous H_2 supply (~\$0.003 per L), resulting in a total estimated cost of \$4.84-\$7.35 per L.

All the prices used to estimate the cost were obtained from publicly available online listings for bulk-pack reagents from major suppliers and commercial distributors. Therefore, these values should be interpreted as order-of-magnitude estimates rather than absolute costs. In addition, the calculations above represent a first-run estimate, i.e., the full material inputs were charged to a single treatment run to enable a consistent comparison of the dominant cost contributors between the two systems. In continuous operation, catalyst reuse could reduce the effective cost per treated volume. We further calculated the operating expenditure below.

Operating expenditure (OPEX) comparison

For the MoS_2–NC/Fe^0 system, the OPEX includes (i) Fe^0 consumption (supplemental Fe^0 dosing), (ii) solid/sludge handling and disposal, (iii) MoS_2–NC supplementation, and (iv) utilities/maintenance. For 1 mM ClO_4^- removed per m^3 (1 mol/m^3), the stoichiometric Fe^0 demand is 0.223 kg/m^3. With an electron efficiency of 20%-40%, the net Fe^0 demand is 0.56-1.12 kg/m^3, corresponding to \$2.5-\$11.5/m^3 using the industrial Fe^0 price mentioned above. The consumed Fe is converted to $Fe(OH)_2$/$Fe(OH)_3$ solids, which requires separation, dewatering, and disposal. Using reported dewatered sludge disposal cost benchmarks (e.g., 300-800/dry ton),[43,44] and kg-scale dry solids generation per m^3 (~1-2 kg dry solids per m^3, depending on $Fe(OH)_x$ form), the sludge handling/disposal cost is estimated as \$0.3-\$1.9/m^3. For MoS_2–NC supplementation, the initial catalyst input was estimated to \$18-\$76/m^3 as discussed above. The deactivation fraction per run was calculated from the half-life and run time. From the Fig. 3h, the half-life was estimated to be 3.3 days, with a run time of 18 h (Supplementary Fig. 32) and 1% catalyst lost for each run. Because each run removes only 80% of ClO_4^-, the catalyst cost per m^3 treated was normalized to report cost per 1 mM removed. The cost of the catalyst supplementation would be \$3.50-\$14.80/m^3. Finally, for utilities and maintenance, wastewater treatment energy use commonly is 0.30-1.89 kWh/m^3, while routine maintenance is site-specific but ignorable relative to material supplementation and solids handling.[45,46] Given the U.S. industrial electricity price benchmark (~\$0.081/kWh), the electricity cost is \$0.02-\$0.15/m^3. Summing these terms, the estimated OPEX

for MoS_2–NC/Fe^0 is ~\$6.27-\$28.25 per m^3 (for 1 mM ClO_4^- removed), dominated by Fe^0 consumption and sludge management.

For the Pd/H_2-based [$(NH_2)_2$*bpy*]MoO_x–Pd/C system, the OPEX should include (i) catalyst supplementation, (ii) H_2 supply, (iii) acidification to pH 3 and subsequent neutralization, and (iv) utilities/maintenance. On the same removal basis used above (1 mM ClO_4^- removed per m^3), the conversion of ClO_4^- to Cl^- is an 8-electron reduction, implying a stoichiometric H_2 demand of 4 mol H_2 per mol ClO_4^- (~0.008 kg H_2/m^3). Assuming 1-2× excess to cover inefficiencies, this corresponds to 0.008-0.016 kg H_2/m^3 and account for \$0.05-\$0.29/m^3 (\$6.2-18/kg H_2 from NREL Annual Technology Baseline (ATB) 2024). For pH adjustment, the chemical cost is estimated as \$0.03-\$0.11/m^3.[47] Electricity is the same as above, which is \$0.02-\$0.15/m^3. In addition, catalyst supplementation dominates the OPEX of Pd/H_2 system. The first-run estimate of the catalyst is \$4.74-\$7.25 per L if treated as single-pass dosing (i.e., no reuse). The half-life was calculated to be 4.8 days according to the reported rate decay. Assuming 1 h hydraulic retention time (HRT, estimated with batch cycle time) and 1% of the catalyst lost for each run, the cost of catalyst supplementation would be \$75.9-\$116.2/m^3. The total cost would be estimated as \$76.0-\$116.8/m^3.

Capital expenditure (CAPEX) comparison

For the MoS_2–NC/Fe^0 system, capital costs are primarily driven by (i) reactor volume and (ii) solid separation and dewatering systems. Under the long HRT (18 h) used for the reduction process, the reactor requires large tankage, with mixers, pumps, and piping designed for slurry. Because Fe^0 is sacrificial and converts to $Fe(OH)_2$/$Fe(OH)_3$ solids during reaction, the system typically requires solid-liquid separation followed by mechanical dewatering (e.g., clarifier or thickener coupled with belt press, centrifuge, or filter press), along with sludge handling and loadout. Sludge management (including dewatering, handling, and transport interfaces) is widely identified as a major design and costing element in municipal and industrial wastewater facilities,[48-50] and thus is expected to be a dominant CAPEX contributor for Fe^0-based treatment. Overall, this system has moderate mechanical complexity (no combustible gas system), but high installed cost sensitivity to reactor volume and sludge management.

For the [$(NH_2)_2$*bpy*]MoO_x–Pd/C system, A gas-liquid reactor is generally required, which can be packed bed, trickle bed, or stirred reactor with gas sparging. Because H_2 delivery to the liquid phase can be rate-limiting, it requires facilities to enhance mass transfer, like fine-bubble

gas diffusion and mild pressurization, which increases mechanical and controls complexity relative to an all-liquid system. The core CAPEX item is the hydrogen supply and safety infrastructure,[51] including H_2 storage (gas container or onsite supply), regulation and piping, and safety systems (hydrogen detection, alarms/interlocks, and ventilation) to prevent accumulation of flammable mixtures. Meanwhile, the acidification and subsequent neutralization generally requires chemical storage tanks, metering pumps, mixers. Overall CAPEX implication: Lower tank volume (shorter HRT) can reduce reactor civil costs, but the hydrogen supply system with safety system and pH adjustment tends to push CAPEX to moderate-high, and it is more permitting-/hazard-driven than the Fe^0 system. Consequently, although shorter HRT can reduce reactor costs, the hydrogen supply and safety systems and pH-adjustment infrastructure lead to overall moderate-high CAPEX.

Overall, the Fe^0-based system tends to have CAPEX dominated by reactor volume and solids management, whereas the Pd/H_2 is dominated by hydrogen supply and safety systems and pH adjust infrastructure. Practically, this implies that Fe^0 systems often have lower hazard-driven CAPEX but higher solids-management CAPEX, while Pd/H_2 systems are the opposite due to hydrogen handling and safety compliance.

CAPEX contributor table for the two systems

Category	MoS_2–NC/Fe^0 system	Pd/H_2 system	Notes
Reactor	Large volume (long HRT)	Smaller volume (short HRT)	
Gas supply system	None	H_2 storage, regulation and piping	H_2 infrastructure is a distinct capital item (gas supply, controls, venting).
Hydrogen safety system	None	Detectors, alarms/interlocks, ventilation	H_2 facilities typically require detector-triggered shutdown/alarms and ventilation facilities to keep H_2 below flammability thresholds.
Solid separation	Required	Required for catalyst recycle	Fe^0 generates solids continuously, so solid-liquid separation is necessary. Pd/C may need filtration if fines need to be retained for recycling.

Dewatering equipment	Required	None	Mechanical dewatering is often a major cost contributor.
Sludge handling facilities	Required	None	$Fe(OH)_x$ solids require handling space and equipment.
Acid/base storage and dosing system	Optional	Required for acidification and neutralization	Pd/H_2 at pH 3 needs acid and base feed systems (tanks, mixers, metering pumps)
Materials of construction	Corrosion considerations	Acid and H_2 compatibility	Fe slurries require wear-resistant design; Pd/H_2 requires acid-resistant wetted parts and H_2-safe components.
Instrumentation and controls (I&C)	Standard water controls	Enhanced safety for I&C	Pd/H_2 typically requires additional safety devices (detectors, control system, hardware/software for H_2 ventilation status)

Supplementary References

(1) Tsai, C.; Li, H.; Park, S.; Park, J.; Han, H. S.; Nørskov, J. K.; Zheng, X.; Abild-Pedersen, F. Electrochemical generation of sulfur vacancies in the basal plane of MoS_2 for hydrogen evolution. *Nat. Commun.* **2017,** *8* (1), 15113.

(2) Li, H.; Tsai, C.; Koh, A. L.; Cai, L.; Contryman, A. W.; Fragapane, A. H.; Zhao, J.; Han, H. S.; Manoharan, H. C.; Abild-Pedersen, F.; Nørskov, J. K.; Zheng, X. Activating and optimizing MoS_2 basal planes for hydrogen evolution through the formation of strained sulphur vacancies. *Nat. Mater.* **2016,** *15* (1), 48-53.

(3) Błoński, P.; Tuček, J.; Sofer, Z.; Mazánek, V.; Petr, M.; Pumera, M.; Otyepka, M.; Zbořil, R. Doping with graphitic nitrogen triggers ferromagnetism in graphene. *J. Am. Chem. Soc.* **2017,** *139* (8), 3171-3180.

(4) Xu, L.; Kirvassilis, D.; Bai, Y.; Mavrikakis, M. Atomic and molecular adsorption on Fe(110). *Surf. Sci.* **2018,** *667*, 54-65.

(5) Cao, Q.; Dai, J.; Hao, Z.; Paulus, B.; Eigler, S.; Chen, X. Controllable graphene/MoS_2 heterointerfaces by perpendicular surface functionalization. *Angew. Chem. Int. Ed.* **2024,** *63* (51), e202415922.

(6) Xu, Y.; Wu, D.; Zhang, Q.; Rao, P.; Deng, P.; Tang, M.; Li, J.; Hua, Y.; Wang, C.; Zhong, S.; Jia, C.; Liu, Z.; Shen, Y.; Gu, L.; Tian, X.; Liu, Q. Regulating Au coverage for the direct oxidation of methane to methanol. *Nat. Commun.* **2024,** *15* (1), 564.

(7) Guo, Z.; Li, F.; Xiao, Y. C.; Hung, S.-F.; Lu, Y.-R.; Foroozan, A.; Liu, J.; Sun, S. S.; Liu, S.; Che, Y.; Wang, Q.; Liu, M.; Wang, C.; Li, Y.; Peng, K.-S.; Liu, Y.-C.; Fan, M.; Azimi Dijvejin, Z.; Papangelakis, P.; Wang, Y.; Shayesteh Zeraati, A.; Han, K.; Corbett, P.; Higgins, D.; Miao, R. K.; Sinton, D. Efficient amino-acid-based reactive capture of CO_2 via nickel molecular catalyst. *Nat. Commun.* **2025,** *16* (1), 10373.

(8) Mathew, K.; Kolluru, V. S. C.; Mula, S.; Steinmann, S. N.; Hennig, R. G. Implicit self-consistent electrolyte model in plane-wave density-functional theory. *J. Chem. Phys.* **2019,** *151* (23), 234101.

(9) Kresse, G.; Furthmüller, J. Efficiency of ab-initio total energy calculations for metals and semiconductors using a plane-wave basis set. *Computational Materials Science* **1996,** *6* (1), 15-50.

(10) Perdew, J. P.; Burke, K.; Ernzerhof, M. Generalized gradient approximation made simple. *Phys. Rev. Lett.* **1996,** *77* (18), 3865-3868.

(11) Grimme, S.; Antony, J.; Ehrlich, S.; Krieg, H. A consistent and accurate *ab initio* parametrization of density functional dispersion correction (DFT-D) for the 94 elements H-Pu. *J. Chem. Phys.* **2010,** *132* (15), 154104.

(12) Grimme, S.; Ehrlich, S.; Goerigk, L. Effect of the damping function in dispersion corrected density functional theory. *J. Comput. Chem.* **2011,** *32* (7), 1456-65.

(13) Park, J. S.; Lim, H.-K.; Hu, C.; Lee, E.; Jeon, H. S.; Ryu, J.; Oh, S.; Lee, T. K.; Park, S.; Cho, H. K.; Yu, S.-H.; Ahn, D.; Lee, Y. M.; Kim, M.-G.; Yoo, S. J. Oxide-hybridized carbon as a catalyst support for efficient anion exchange membrane water electrolysis. *Nat. Commun.* **2025,** *16* (1), 11090.

(14) Yu, M.; Trinkle, D. R. Accurate and efficient algorithm for Bader charge integration. *J. Chem. Phys.* **2011,** *134* (6), 064111.

(15) Wang, V.; Xu, N.; Liu, J.-C.; Tang, G.; Geng, W.-T. VASPKIT: A user-friendly interface facilitating high-throughput computing and analysis using VASP code. *Comput. Phys. Commun.* **2021,** *267*, 108033.

(16) Ringe, S.; Hörmann, N. G.; Oberhofer, H.; Reuter, K. Implicit solvation methods for catalysis at electrified interfaces. *Chem. Rev.* **2022,** *122* (12), 10777-10820.

(17) Karimadom, B. R.; Meyerstein, D.; Kornweitz, H. Calculating the adsorption energy of a charged adsorbent in a periodic metallic system – the case of BH_4^- hydrolysis on the Ag(111) surface. *Phys. Chem. Chem. Phys.* **2021,** *23* (45), 25667-25678.

(18) Raju Karimadom, B.; Varshney, S.; Zidki, T.; Meyerstein, D.; Kornweitz, H. DFT study of the BH_4^- hydrolysis on Au(111) surface. *Chem. Phys. Chem.* **2022,** *23* (13), e202200069.

(19) Liu, H.; Huang, J.; Ma, S.; Xiong, R.; Zhao, J.; Fu, Q.; Wei, H.; Liu, Z.; Wang, X.; Yao, T.; Song, B. Valence-engineering of CeO_2 redox modulator boosts the oxygen electrocatalysis performance in Fe/Co dual-atom catalyst. *Adv. Sci.* **2025,** *n/a* (n/a), e16405.

(20) Henkelman, G.; Uberuaga, B. P.; Jónsson, H. A climbing image nudged elastic band method for finding saddle points and minimum energy paths. *Comput. Mater. Sci.* **2000,** *113* (22), 9901-9904.

(21) Pokharel, J.; Cresce, A.; Pant, B.; Yang, M. Y.; Gurung, A.; He, W.; Baniya, A.; Lamsal, B. S.; Yang, Z.; Gent, S.; Xian, X.; Cao, Y.; Goddard, W. A.; Xu, K.; Zhou, Y. Manipulating the diffusion energy barrier at the lithium metal electrolyte interface for dendrite-free long-life batteries. *Nat. Commun.* **2024,** *15* (1), 3085.

(22) Li, L.; Hu, Z.; Kang, Y.; Cao, S.; Xu, L.; Yu, L.; Zhang, L.; Yu, J. C. Electrochemical generation of hydrogen peroxide from a zinc gallium oxide anode with dual active sites. *Nat. Commun.* **2023,** *14* (1), 1890.

(23) Cheng, T.; Xiao, H.; Goddard, W. A. Full atomistic reaction mechanism with kinetics for CO reduction on Cu(100) from ab initio molecular dynamics free-energy calculations at 298 K. *Proc. Nati. Acad. Sci.* **2017,** *114* (8), 1795-1800.

(24) Min, Y.; Zhou, X.; Chen, J.-J.; Chen, W.; Zhou, F.; Wang, Z.; Yang, J.; Xiong, C.; Wang, Y.; Li, F.; Yu, H.-Q.; Wu, Y. Integrating single-cobalt-site and electric field of boron nitride in dechlorination electrocatalysts by bioinspired design. *Nat. Commun.* **2021,** *12* (1), 303.

(25) Kabir, S.; Artyushkova, K.; Serov, A.; Kiefer, B.; Atanassov, P. Binding energy shifts for nitrogen-containing graphene-based electrocatalysts–experiments and DFT calculations. *Surf. Interface Anal.* **2016,** *48* (5), 293-300.

(26) Serov, A.; Artyushkova, K.; Atanassov, P. Fe−N−C oxygen reduction fuel cell catalyst derived from carbendazim: Synthesis, structure, and reactivity. *Adv. Energy Mater.* **2014,** *4* (10), 1301735.

(27) Qin, H.; Zha, T.; Qian, K.; Sun, Y.; Guan, X.; Chen, C. Efficient full dechlorination of chlorinated ethenes on single enzyme-like Co−N_4 sites in nitrogen-doped carbons. *Appl. Catal. B Environ.* **2023,** *328*, 122459.

(28) Pan, X.; Song, X.; Lin, S.; Bi, K.; Hao, Y.; Du, Y.; Liu, J.; Fan, D.; Wang, Y.; Lei, M. A facile route to graphite-tungsten nitride and graphite-molybdenum nitride nanocomposites and their ORR performances. *Ceram. Int.* **2016,** *42* (14), 16017-16022.

(29) Huang, Y.; Ge, J.; Hu, J.; Zhang, J.; Hao, J.; Wei, Y. Nitrogen-doped porous molybdenum carbide and phosphide hybrids on a carbon matrix as highly effective electrocatalysts for the hydrogen evolution reaction. *Adv. Energy Mater.* **2018,** *8* (6), 1701601.

(30) Ren, C.; Yang, P.; Sun, J.; Bi, E. Y.; Gao, J.; Palmer, J.; Zhu, M.; Wu, Y.; Liu, J. A bioinspired molybdenum catalyst for aqueous perchlorate reduction. *J. Am. Chem. Soc.* **2021,** *143* (21), 7891-7896.

(31) Ren, C.; Bi, E. Y.; Gao, J.; Liu, J. Molybdenum-catalyzed perchlorate reduction: Robustness, challenges, and solutions. *ACS ES&T Eng.* **2022,** *2* (2), 181-188.

(32) Liu, J.; Choe, J. K.; Wang, Y.; Shapley, J. R.; Werth, C. J.; Strathmann, T. J. Bioinspired complex-nanoparticle hybrid catalyst system for aqueous perchlorate reduction: Rhenium speciation and its influence on catalyst activity. *ACS Catal.* **2015,** *5* (2), 511-522.

(33) Zhang, Y.; Hurley, K. D.; Shapley, J. R. Heterogeneous catalytic reduction of perchlorate in water with Re−Pd/C catalysts derived from an Oxorhenium(V) molecular precursor. *Inorg. Chem.* **2011,** *50* (4), 1534-1543.

(34) Yao, F.; Zhong, Y.; Yang, Q.; Wang, D.; Chen, F.; Zhao, J.; Xie, T.; Jiang, C.; An, H.; Zeng, G.; Li, X. Effective adsorption/electrocatalytic degradation of perchlorate using Pd/Pt supported on N-doped activated carbon fiber cathode. *J. Hazard. Mater.* **2017,** *323*, 602-610.

(35) Wang, D. M.; Huang, C. P. Electrodialytically assisted catalytic reduction (EDACR) of perchlorate in dilute aqueous solutions. *Sep. Purif. Technol.* **2008,** *59* (3), 333-341.

(36) Shih, Y.-J.; Wu, Z.-L.; Hsu, C.-H. Perchlorate decomposition on rhodium nanoclusters supported on copper crystallite (RhxCu) electrode in an electrodialysis-assisted bipolar electro-reduction system (ED-ER). *Chem. Eng. J.* **2024,** *481*, 148477.

(37) Almassi, S.; Ren, C.; Liu, J.; Chaplin, B. P. Electrocatalytic perchlorate reduction using an oxorhenium complex supported on a Ti_4O_7 reactive electrochemical membrane. *Environ. Sci. Technol.* **2022,** *56* (5), 3267-3276.

(38) Wang, C.; Huang, Z.; Lippincott, L.; Meng, X. Rapid Ti(III) reduction of perchlorate in the presence of β-alanine: Kinetics, pH effect, complex formation, and β-alanine effect. *J. Hazard. Mater.* **2010,** *175* (1), 159-164.

(39) Park, S. H.; Batchelor, B.; Lee, C.; Han, D. S.; Abdel-Wahab, A. Perchlorate degradation using aqueous titanium ions produced by oxidative dissolution of zero-valent titanium. *Chem. Eng. J.* **2012,** *192*, 301-307.

(40) Lehman, S. G.; Badruzzaman, M.; Adham, S.; Roberts, D. J.; Clifford, D. A. Perchlorate and nitrate treatment by ion exchange integrated with biological brine treatment. *Water Res.* **2008,** *42* (4), 969-976.

(41) Maneewong, Y.; Chaemchuen, S.; Verpoort, F.; Klomkliang, N. Paracetamol removal from water using N-doped activated carbon derived from coconut shell: Kinetics, equilibrium, cost analysis, heat contributions, and molecular-level insight. *Chem. Eng. Res. Des.* **2022,** *185*, 163-175.

(42) Lai, J. Y.; Ngu, L. H. The production cost analysis of oil palm waste activated carbon: a pilot-scale evaluation. *Greenhouse Gases: Science and Technology* **2020,** *10* (5), 999-1026.

(43) Peccia, J.; Westerhoff, P. We should expect more out of our sewage sludge. *Environ. Sci. Technol.* **2015,** *49* (14), 8271-8276.

(44) Mulchandani, A.; Westerhoff, P. Recovery opportunities for metals and energy from sewage sludges. *Bioresour. Technol.* **2016,** *215*, 215-226.

(45) Walker, N. L.; Williams, A. P.; Styles, D. Pitfalls in international benchmarking of energy intensity across wastewater treatment utilities. *J. Environ. Manage.* **2021,** *300*, 113613.

(46) Gandiglio, M.; Lanzini, A.; Soto, A.; Leone, P.; Santarelli, M. Enhancing the energy efficiency of wastewater treatment plants through co-digestion and fuel cell systems. *Fron. Environ. Sci.* **2017,** *5*, 70.

(47) Alabduljalil, S.; Alotaibi, S.; Abdulrahim, H. Techno-economic evaluation of different seawater reverse osmosis configurations for efficient boron removal. *Desalin. Water Treat.* **2019,** *168*, 65-76.

(48) Wang, S.; Sadhukhan, J.; Xuan, J.; Yu, Y.; Mao, X.; Wang, M.; Chen, X.; Zhou, X.; Xing, L.; Wang, X. Life cycle assessment and life cycle cost of sludge dewatering, conditioned with

Fe^{2+}/H_2O_2, $Fe^{2+}/Ca(ClO)_2$, $Fe^{2+}/Na_2S_2O_8$, and Fe^{3+}/CaO based on pilot-scale study data. *ACS Sustainable Chem. Eng.* **2023,** *11* (20), 7798-7808.

(49) Stickland, A. D.; Skinner, S. J.; Cavalida, R. G.; Scales, P. J. Optimisation of filter design and operation for wastewater treatment sludge. *Sep. Purif. Technol.* **2018,** *198*, 31-37.

(50) Murray, A.; Horvath, A.; Nelson, K. L. Hybrid life-cycle environmental and cost inventory of sewage sludge treatment and end-use scenarios: A case study from China. *Environ. Sci. Technol.* **2008,** *42* (9), 3163-3169.

(51) Morrissey, K. G.; English, L.; Thoma, G.; Popp, J. Prospective life cycle assessment and cost analysis of novel electrochemical struvite recovery in a U.S. wastewater treatment plant. *Sustainability* **2022,** *14* (20), 13657.
