# Supplementary material for: Donor−acceptor catalysis with defective MoS2 and Fe0 breaks barriers to perchlorate reduction under mild conditions
Source: Nat Commun. 2026 May 20;17:6721. doi: 10.1038/s41467-026-73219-5 (PMC13385889; doi:10.1038/s41467-026-73219-5)
Supplement: Supplementary file 2 — Transparent Peer Review file [file 41467_2026_73219_MOESM2_ESM.pdf]

nature portfolio

Peer Review File

Donor–acceptor catalysis with defective MoS_2 and Fe^0 breaks barriers to perchlorate reduction under mild conditions

Corresponding Author: Professor Xiaohong Guan

This file contains all reviewer reports in order by version, followed by all author rebuttals in order by version.

Version 0:

Reviewer comments:

Reviewer #1

(Remarks to the Author)
This study reports the development of a catalyst based on defective MoS2 supported by N-doped carbon (MoS2-NC), which, when combined with nanoscale zero-valent iron (nZVI), effectively reduces perchlorate to chloride at mild reaction conditions. The work is technically sound, provides a comprehensive dataset combining experimental and computational results, and addresses an environmentally important challenge. However, the conceptual advance in the catalyst design relative to the authors' previous MoS_2/ZVI study focused on the removal of chlorinated hydrocarbons (Chen et al., 2025, DOI: 10.1021/acs.est.5c00564) appears limited. Furthermore, the technoeconomic analysis relies on unrealistic assumptions regarding nZVI costs, undermining one of the paper's main claims about practical competitiveness. Given these issues, I do not believe the manuscript reaches the level of novelty and impact expected for publication in Nature Communications. However, it could make a valuable contribution to a more specialized journal in environmental catalysis or water treatment. My specific recommendations on how to improve the quality of the manuscript are provided below.
1) The claim that DFT calculations guided the catalyst design is not convincing. DFT calculations use simplified models that do not capture the whole complexity of Fe-based heterogenous systems, such as explicit solvation (here no solvation was used) and surface corrosion phenomena. Consequently, DFT in this context should primarily be used to rationalize, rather than direct, experimental findings. Moreover, the authors already developed a closely related catalyst in the past (see DOI: 10.1021/acs.est.5c00564). In particular, the performed DFT calculations do not support the chosen catalyst design in two aspects: (i) The purported enhancement of electronic conductivity by N-doped carbon cannot be substantiated from the calculations shown in Figure 1a. The two slabs behave largely as isolated systems, with only weak interactions between MoS_2 and CN in the composite slab model. In contrast, the effect of reducing the MoS2 band gap can be clearly seen when Fe is introduced to MoS2 (Figure S2b), raising the question whether the carbon component is necessary at all. (ii) The reported Gibbs free-energy changes for the hydrogenation of adsorbed O as well as for the overall OH- release appear too large (0.8 and 1 eV, Figure 1f) for the reaction to proceed at a reasonable time scale at mild temperature. Did the authors attempt to calculate the corresponding reaction barriers for these steps? It is also inconsistent that elsewhere (line 123) the manuscript describes a barrier of 0.66 eV as "significantly high".
2) The role of N-doped carbon requires further justification. The authors state that the N-doped carbon is necessary for MoS2 to exhibit catalytic activity (lines 336-339). However, in their previous study, they showed that ball milling of MoS2 can also provide defective sites, leading to efficient degradation of hexachloroethane in a galvanic system. It would be therefore informative to include experiments testing ball-milled MoS2 without the NC support for perchlorate reduction.
3) The characteristics of Fe(0), such as particle size, morphology, specific surface area, and surface properties are not documented in the manuscript except for the zeta potential measurements (Figure S12). These should be studied in more detail and their effects on the perchlorate removal performance should be evaluated.
4) The technoeconomic analysis is flawed and misleading. It is based on unrealistic Fe(0) price (0.8 USD/kg, line 384) corresponding to bulk iron and not nZVI, which typically costs above 100 USD/kg. This would make the more conventional Pd/H2 process more economical. It is also noteworthy that the manuscript consistently avoids using the term "nano-ZVI," even though the borohydride-reduction route used is a well-established method for preparing nanoscale Fe(0). This omission gives the impression that the cost assumptions were chosen to make the proposed process appear more favorable than it realistically is.
5) I would suggest moving the nZVI synthesis description into the material synthesis part in the methodology section and acknowledging the fact that it is nanoscale Fe(0).

6) To improve the cost efficiency of the described process, I would also suggest the authors to conduct experiments with coarser (and hence cheaper) Fe(0) materials.
7) More information should be provided in the methodology section: how were the transition states and Gibbs free energies calculated, what were the operating voltages of electron microscopes, and description of zeta potential, XRD, and Raman analyses.

Reviewer #2

(Remarks to the Author)
The manuscript reports a MoS_2–NC/Fe^0 donor–acceptor system capable of rapidly reducing ClO_4^- under mild conditions. The concept is compelling, and the experimental performance together with the comprehensive characterization provides a strong foundation for the study. The mechanistic interpretation, particularly the electronic role of the NC support and the DFT analysis, would benefit from further clarification and more quantitative support. Certain aspects of the computational modeling and technoeconomic comparison appear simplified relative to the complexity of the proposed mechanism. Addressing the points below would substantially strengthen the mechanistic rigor and overall clarity of the work. I recommend major revision, and several main concerns are discussed below.

1. The DFT model used appears inconsistent with the proposed mechanism, failing to provide any evidence to support the core argument that NC is the key electronic mediator. Figure 1B shows almost no electron transfer between NC and MoS2, indicating a weak physical interaction between them. The authors should address this inconsistency, for example, by discussing the limitations of the model or providing computational results that more directly reflect the proposed mechanism.

2. The claim that NC "introduces additional electronic states" to enhance conductivity lacks a proper comparative basis. The intrinsic conductivity of Fe is far superior to that of NC, and the DOS of bulk Fe is much higher at the Fermi level. To be substantiated, this would require a comparison of the electronic structures of an isolated defective MoS2 system against the composite MoS2-NC system, which is currently absent.

3. The interpretation of the calculated electronic structure warrants revision for accuracy. First, the characterization of MoS2 as a "semiconductor" is not rigorously supported by the DOS in Fig. 1a, which shows a significant DOS at the Fermi level. Second, the analysis of the Mo PDOS (Supp. Fig. 2) appears inconsistent with the data presented. The text claims the d-band shifts "away" from the Fermi level, whereas the plots indicate a slight shift toward the Fermi level. Therefore, the conclusion that this shift weakens the Mo-O interaction lacks basis and may contradict established theories (such as the d-band center theory). A more precise analysis is needed to support the manuscript's mechanistic claims.

4. The authors provide a comprehensive characterization of the defective nature of the MoS2 phase, which is convincingly linked to its catalytic activity. However, the nature of the interaction at the interface between the MoS2 crystallites and the NC support remains ambiguous (e.g., strong physisorption via van der Waals forces versus covalent bonding). Elucidating the properties of this interface is crucial for understanding the role of NC in the system.

5. Experimental results indicate that exposed Mo atoms with S vacancies are the catalytic sites. It is suggested that the authors provide comparative calculations (e.g., ClO_4^- adsorption energies and activation barriers) on the pristine MoS_2 surface compared to surfaces with varying degrees/types of S-vacancies to validate this key claim theoretically.

6. The computational methods section lacks sufficient detail for reproducibility. Please provide more specifics on the computational setup—e.g., details on the slab model construction, the method used for transition state searching, and the specifics of the free energy calculations and any solvent models applied.

7. The evidence for the second role of the NC support (lines 336-357) is not clear. A contradiction exists between the experimental XPS results (showing no Mo-N bonds) and the proposed mechanism from cited literature (Refs 52, 53), which relies on Mo-N bond formation to create S-vacancies. It is suggested that the authors provide more direct evidence, such as comparative EPR data for the MoS_2-AC and MoS_2-AC+N control materials, to substantiate the claim that N-doping increases S-vacancy concentration.

8. The technoeconomic analysis is useful but appears oversimplified. Considering only the metal cost does not fully capture the OPEX and CAPEX differences between Fe^0-based and Pd/H_2 systems. Fe^0 corrosion, iron sludge generation, solid–liquid separation, catalyst replacement frequency, and carbon-support cost can significantly influence the overall economics and should be discussed for a more realistic comparison. A performance-normalized cost comparison (e.g., equal treatment capacity or equivalent benefit-cost ratio) would further enhance the robustness of the analysis. Clarifying the assumed catalyst lifetimes and including basic sensitivity analyses would also help substantiate the claim of cost competitiveness.

Reviewer #3

(Remarks to the Author)
The manuscript "Donor–acceptor catalysis with defective MoS2 and Fe0 breaks barriers to perchlorate reduction under mild conditions" is a joint computational-experimental study, and I was asked for consideration of the computational part.

My problem with the computational part of the manuscript is that, in its current form, it is irreproducible, the computational

model is not well described, the methods are questionable, and the conclusions are not sufficiently supported by the obtained data.

In more detail:
1. Computational model: In "Computational methodology" section (page 20), it is just stated "The computational models were constructed based on characterization results, with sulfur vacancies introduced into the MoS2 crystal structure to simulate defect sites" and link to Supplementary Figure 1, however, there is only top and side structural model and it is not clear, a) what is the unit cell, b) how MoS2/CN interface was constructed (incommensurate supercell?), c) why just six atoms is included in Fe cluster, etc.
2. In addition, it is not clear if there is a right ground state of the system. On the one hand, it is stated (page 20) that "Spin-polarized density functional theory" was used; on the other hand, no information on the resulting spin distribution (spin density) is provided. The most complicated part of the model, the iron cluster, is not an easy task, and, typically, a high-spin solution is preferred. In Phys. Chem. Chem. Phys., 2020, 22, 23258, e.g., Fe4 cluster used for molecular adsorption embodied 13-tet configuration (i.e., multiplicity 13, magnetic moment 12 muB, ESI there) or Fe7 cluster is of multiplicity 23.
3. Moreover, it is stated that "ClO4− preferentially adsorbs at undercoordinated Mo sites over S sites" (page 5). But negatively charged ions in periodic boundary conditions are a very challenging task (lattice sums over charged cells diverge), and there is no mention of how exactly this was modelled. By restricting the number of electrons in the input file? By some other counterion? Via some special corrections?
4. Method: "generalized gradient approximation (GGA) with the Perdew-Burke-Ernzerhof (PBE) functional" was used (page 20), but the interface depicted in Supplementary Figure 1 contains non-covalent interactions (surely between MoS2 and CN), which are not included in pure GGA density functionals. Geometry optimizations (finding local minima) should be done using dispersion-corrected DFT (either by semiempirical correction like D3 or using density functionals from the family of nonlocal van der Waals density functionals as optB86b-vdW), see e.g. J. Am. Chem. Soc. 135(16), 6372-6377, 2013. What is the nature of the interaction between MoS2 and CN, or between MoS2 and Fe6?
5. In addition, it is stated "Density of states (DOS) analysis shows that MoS2 retains a semiconducting feature, while the NC support introduces additional electronic states near the Fermi level, narrowing the band gap." (Page 5) But the Fermi level (0 eV) crosses both MoS2 and CN states; therefore, it does not make sense.
6. "Gibbs free energies for all the states were calculated based on the zero-point energy-corrected DFT total energy, which was set as the enthalpy at 0 K" (page 20). No details are provided; does it mean that phonon calculations were performed? If this is enthalpy at 0 K, which pressure was considered?

Text also contains a lot of typos, such as "Brillion zone" instead of "Brillouin zone", etc.

In this form, I cannot recommend publication, at least of the computational part.

Version 1:

Reviewer comments:

Reviewer #2

(Remarks to the Author)
Comments to the Authors
I co-reviewed this manuscript with one of the reviewers who provided the listed reports. This is part of the Nature Communications initiative to facilitate training in peer review and to provide appropriate recognition for Early Career Researchers who co-review manuscripts.

The authors have carefully revised the manuscript and addressed the main concerns raised in the first round of review. The use of periodic DFT slab models, the clarification of the technoeconomic analysis, and the expanded mechanistic discussion have substantially improved the manuscript. In my view, the main issues raised previously have been satisfactorily resolved, and the conclusions are now adequately supported by the experimental and computational results. Overall, the manuscript is scientifically sound and close to being suitable for publication. I recommend acceptance after minor revision to address the following points.
1. The disappearance of ClO_4^- should be described as pseudo-first-order kinetics, not first-order kinetics. Please revise this terminology consistently in the main text and Supplementary Information.
2. The HRTEM discussion should be slightly refined. Distorted or discontinuous lattice fringes support structural disorder or defective domains, but standard HRTEM does not directly identify individual sulfur vacancies. Please link the vacancy assignment more explicitly to the EPR results and related evidence.
3. The description of the MoS_2–NC interface could be slightly moderated. The absence of detectable Mo–C or Mo–N bonds supports a predominantly noncovalent interface, but the DFT results also indicate interfacial charge redistribution. The wording could therefore be slightly moderated to clarify that the interface is predominantly noncovalent while still involving electronic coupling between the two components.
4. The revised manuscript is somewhat dense in places. A light editing pass to reduce minor redundancies and improve readability may help clarify the main storyline, although this is not essential for acceptance.

Reviewer #3

(Remarks to the Author)
The manuscript "Donor–acceptor catalysis with defective MoS2 and Fe0 breaks barriers to perchlorate reduction under mild conditions" is a joint computational-experimental study, and I was asked to consider the computational part.

I appreciate the authors' efforts in the computational part of the manuscript. It is surprisingly completely recalculated; however, it seems it was necessary. The computational model is now well described, the methods are clear and transparent, and the statements based on calculations are sufficiently supported by the obtained data.

I have only minor comments:
1. ClO4− configuration on MoS2 changes from the last version. Could the authors confirm they tried more adsorption sites and ClO4− orientations?
2. The authors used the PBE-D3 method, which is the Perdew-Burke-Ernzerhof functional with Grimme's D3 dispersion correction, for optimisations compared to PBE (Perdew-Burke-Ernzerhof functional without dispersion correction) in the original versions. What is the nature of the interaction between ClO4− (perchlorate anion) and MoS2 (molybdenum disulfide)? Following the interaction energies, it should be a non-covalent interaction. But if this is dominating, how is it possible that the interaction energies drastically decreased from the last version (PBE) to the new version (PBE-D3)? (about one half: -0.70 electronvolts (eV) (old) to -0.37 eV (new), MoS2-NC case, Figure 1) . It seems contra-intuitive.
3. The authors stated, "For anionic adsorbates (e.g., ClO4−), a Na+ counterion was introduced in the cell to maintain charge neutrality." This seems fine. But can the authors prove that the actual quantum-mechanical solution of the electronic Schrodinger equation provides the right ions ClO4− and Na+? Another solution, ClO4 and Na (both radicals), is also possible and analysis (e.g., by Bader analysis) is missing.
4. The model seems fine now, e.g., in Supplementary Figure 1. If bonds in MoS2 and CN are depicted by tubes, could the author use the same for iron? At this moment, the iron layer seems rather as amorphous, but it is declared as a (110) slab.

Overall, the improvements made to the computational section have substantially raised the quality of the manuscript. Upon addressing the above minor points, I believe the study will make a valuable contribution to the field.

Reviewer #4

(Remarks to the Author)
MS#: NCOMMS-25-81561
Title: Donor–acceptor catalysis with defective MoS2 and Fe0 breaks barriers to perchlorate reduction under mild conditions
Authors: Prof. Guan et al.

Comments on Reviewer 1's comments and authors' responses

1. Reviewer 1: In the general comments, Reviewer 1 commented that "this work is technically sound, provides a comprehensive dataset combining experimental and computational results, and addresses an environmentally important challenge." However, the reviewer challenged that "the conceptual advance in the catalyst design relative to the authors' previous MoS_2/ZVI study focused on the removal of chlorinated hydrocarbons (Chen et al., 2025, DOI: 10.1021/acs.est.5c00564) appears limited. Furthermore, the technoeconomic analysis relies on unrealistic assumptions regarding nZVI costs, undermining one of the paper's main claims about practical competitiveness." Consequently, Reviewer 1 claimed that "I do not believe the manuscript reaches the level of novelty and impact expected for publication in Nature Communications. However, it could make a valuable contribution to a more specialized journal in environmental catalysis or water treatment.

Authors' responses: About the novelty level and the prior work with MoS2/ZVI (DOI: 10.1021/acs.est.5c00564), the authors provided a thorough justification: 1) the new material has N-doped carbon (NC) added and addressed the unique roles of NC with additional experimental data (especially comparison of the new material with the prior material was quite convincing of the novelty) and computational evidence. 2) The target contaminant perchlorate was indeed different from chlorinate hydrocarbons – the prior material was not working to perchlorate.

For the other major point about the technoeconomic analysis, the authors completely revised the section to provide an order-of-magnitude preliminary estimated and benchmarked with the Pd/H2 system to showcase the advantage.

My comments: The authors provided solid evidence addressing both of the major concerns of Reviewer 1. In addition to the large amount of additional experimental data, the authors completely revised the computational models and added additional characterization data to reveal deeper insights of the material synergy and underlying mechanisms governing the donor-acceptor process.

Overall, the amount of data, the computational evidence, and the depth of the analysis make this paper a strong case and well worthy of publishing in this journal.

The authors spent a great deal of effort in the preliminary cost analysis, as indicated by the other reviewers as well, while such information is important for practical application, such estimates at this level carry lots of uncertainties. As such, benchmarking again some other known technologies should be enough to make a case.
In the subsequent specific comments, Reviewer 1 criticized the claim that "DFT calculations guided the catalyst design is not convincing.", which I agreed, and "The purported enhancement of electronic conductivity by N-doped carbon cannot be

substantiated from the calculations." Again, the authors did a fine job in addressing these concerns by updating their models used.

In addition, I would like to suggest the authors also consider the effect of N-C on the surface hydrophobicity – especially considering perchlorate has a much lower hydration energy compared to common inorganic anions such as sulfate and chloride.

Overall, the authors addressed the comments very well. This work contains tremendous amounts of experimental data and insightful analysis, and is worth publishing.

Open Access This Peer Review File is licensed under a Creative Commons Attribution 4.0 International License, which permits use, sharing, adaptation, distribution and reproduction in any medium or format, as long as you give appropriate credit to the original author(s) and the source, provide a link to the Creative Commons license, and indicate if changes were made.
In cases where reviewers are anonymous, credit should be given to 'Anonymous Referee' and the source.
The images or other third party material in this Peer Review File are included in the article's Creative Commons license, unless indicated otherwise in a credit line to the material. If material is not included in the article's Creative Commons license and your intended use is not permitted by statutory regulation or exceeds the permitted use, you will need to obtain permission directly from the copyright holder.
To view a copy of this license, visit https://creativecommons.org/licenses/by/4.0/

Response to Reviews of NCOMMS-25-81561

Donor−acceptor catalysis with defective MoS_2 and Fe^0 breaks barriers to perchlorate reduction under mild conditions

Hejie Qin, Han Yang, Zhenmin Zhang, Yiran Feng, Yuankui Sun, Peng Fan, Zhimin Ao, T. David Waite, and Xiaohong Guan

We appreciate the detailed and constructive comments from the reviewers, which have helped us significantly in improving the quality of our work. Provided below is the detailed point-to-point response to each comment. The revisions have been highlighted in the manuscript and are also shown below (text in green).

Reviewer #1

General Comments:

This study reports the development of a catalyst based on defective MoS2 supported by N-doped carbon (MoS2-NC), which, when combined with nanoscale zero-valent iron (nZVI), effectively reduces perchlorate to chloride at mild reaction conditions. The work is technically sound, provides a comprehensive dataset combining experimental and computational results, and addresses an environmentally important challenge. However, the conceptual advance in the catalyst design relative to the authors' previous MoS_2*/ZVI study focused on the removal of chlorinated hydrocarbons (Chen et al., 2025, DOI: 10.1021/acs.est.5c00564) appears limited. Furthermore, the technoeconomic analysis relies on unrealistic assumptions regarding nZVI costs, undermining one of the paper's main claims about practical competitiveness. Given these issues, I do not believe the manuscript reaches the level of novelty and impact expected for publication in Nature Communications. However, it could make a valuable contribution to a more specialized journal in environmental catalysis or water treatment. My specific recommendations on how to improve the quality of the manuscript are provided below.*

Thanks for your comments. We carefully considered your concerns and found your recommendations valuable to improve the quality of this study. You have raised two central concerns: (i) the conceptual advance relative to our prior MoS_2/ZVI work (DOI: 10.1021/acs.est.5c00564) and (ii) the realism of the technoeconomic analysis (TEA), particularly with respect to nZVI (Fe^0) cost assumptions.

First, regarding novelty relative to our prior study, we would like to clarify the distinction in target contaminant, material design, and mechanism. The earlier work focused on Fe^0-driven reduction of chlorinated hydrocarbons, whereas MoS_2 acted as a modifier to enhance the reactivity of Fe^0. In contrast, the present manuscript targets perchlorate reduction under mild conditions, which is substantially more challenging and proceeds through oxygen-atom-transfer pathway. The present

study employs a co-mixed donor−acceptor configuration (Fe^0 and pre-fabricated MoS_2−NC), rather than pre-integrating them as a composite in the prior work. To make this distinction evidence-based, we added new control experiments showing that the MoS_2/Fe^0 composite following the prior protocol exhibits negligible perchlorate removal, whereas the NC-enabled MoS_2 catalytic domain is a necessary element to achieve measurable activity. These results directly demonstrate that the prior MoS_2-modified Fe^0 composite does not provide a conceptual or performance basis for perchlorate reduction here.

Second, we agree that the original TEA section could be misleading due to the use of a bulk metal price baseline. We therefore revised the TEA to use commercially relevant Fe^0 prices and updated the analysis on a consistent functional basis. In addition, to address the cost concern of nZVI, we conducted new experiment using a coarser Fe^0 powder (20 mesh) as a low-cost, commercially accessible electron donor. Although a higher Fe^0 dose was required, the system still achieved effective perchlorate reduction, and the revised TEA shows the process can remain economically competitive due to the much lower unit cost of coarser Fe^0.

In addition to these two major revisions, we have revised the manuscript by (i) updating the DFT models and settings and revising the interpretation to support the design rationale; (ii) adding Fe^0 characterization (particle size, BET surface area, microscopy, XRD, and XPS) and quantifying particle-size effects on kinetics; and (iii) updating the Methods sections to include characterization details and the synthesis description of Fe^0, and identify it as nZVI where appropriate, as you recommended. Details are provided below.

Specific Comments:

1) The claim that DFT calculations guided the catalyst design is not convincing. DFT calculations use simplified models that do not capture the whole complexity of Fe-based heterogenous systems, such as explicit solvation (here no solvation was used) and surface corrosion phenomena. Consequently, DFT in this context should primarily be used to rationalize, rather than direct, experimental findings. Moreover, the authors already developed a closely related catalyst in the past (see DOI: 10.1021/acs.est.5c00564). In particular, the performed DFT calculations do not support the chosen catalyst design in two aspects: (i) The purported enhancement of electronic conductivity by N-doped carbon cannot be substantiated from the calculations shown in Figure 1a. The two slabs behave largely as isolated systems, with only weak interactions between MoS_2 and CN in the composite slab model. In contrast, the effect of reducing the MoS2 band gap can be clearly seen when Fe is introduced to MoS2 (Figure S2b), raising the question whether the carbon component is necessary at all. (ii) The reported Gibbs free-energy changes for the hydrogenation of adsorbed O as well as for the overall OH- release appear too large (0.8 and 1 eV, Figure 1f) for the reaction to proceed at a reasonable time scale at mild temperature. Did the authors attempt to calculate the corresponding reaction barriers for these steps? It is also inconsistent that elsewhere (line 123) the manuscript describes a barrier of 0.66 eV as "significantly high".

We agree that the original DFT section requires revision. In response, we have reperformed the DFT calculations with updated models and computational settings

to improve the reliability and interpretability of the results, in order to support the rationale of catalyst design.

First, we improved the models and computational settings. To better simulate the aqueous reaction environment, we used the VASPsol implicit solvation model in all DFT calculations (the methods were elaborated in the SI). While corrosion processes of Fe^0 remain beyond the scope of DFT, the updated solvation-corrected values provide more realistic thermodynamic insights into the OAT and subsequent steps. The updated adsorption energies and free energy diagrams are presented in the revised Fig. 1. We constructed and compared three new models to distinguish the role of each component.

To justify the necessity of the NC layer, we constructed a new model of Fe^0 in contact with NC (MoS_2|NC|Fe^0), and compare it with the MoS_2|Fe^0 and MoS_2|NC models. The adsorption of ClO_4^- is more favorable on MoS_2|NC|Fe^0 (E_{ads} = −0.461 eV), than on MoS_2|Fe^0 (E_{ads} = −0.362 eV), and MoS_2|NC (E_{ads} = −0.28 eV). Meanwhile, Bader charge analysis shows that direct contact of Fe^0 and MoS_2 in the MoS_2|Fe^0 model yields a larger net electron transfer to MoS_2 (4.045 e^-, 3.034 e^-/nm^2). In comparison, Fe^0 transferred 3.270 e^- to NC (2.453 e^-/nm^2), of which 0.511 e^- are transferred to MoS_2 (0.383 e^-/nm^2). However, this did not result in stronger adsorption or improved activation, which may be attributed to the increased electrostatic repulsion toward the anionic oxyanion (ClO_4^-).[1-3] In contrast, the NC layer redistributes the donated electrons from Fe^0 to balance chemical bonding and electrostatic repulsion. This electronic localization adjustment by NC achieves a favorable electronic environment to adsorb oxyanion. These results support the role of NC as an electronic mediator that optimizes (rather than maximizes) electron transfer to the catalytic domain.

Regarding the concern about electronic conductivity, the updated data on MoS_2 slab and MoS_2−NC shows that the band gap is reduced by NC (Supplementary Fig. 2). The introduction of Fe^0 into the model could reduce the band gap of MoS_2, which is due to the metallic electronic states of Fe^0. However, as shown above, the role of NC in our system is not solely a conductivity enhancer. We originally mentioned the conductivity enhancing role to clarify how electrons from Fe^0 can be effectively transferred to the catalytic domain. Now we have revised the manuscript to clarify both of the two roles (efficiently mediating while optimizing the electron transfer from Fe^0 to MoS_2).

We have re-calculated the free energies for the hydrogenation steps under solvation correction. For MoS_2|NC|Fe^0, the updated value for *O → *+OH is +0.203 eV. Although these unit reaction steps remain uphill, it is lower than those in other models (MoS_2|Fe^0, and MoS_2|NC). In addition, the overall reaction free energy accounting for both Cl−O bond cleavage and its O atom dissociation is negative. These results show that although this step is not barrierless, it is consistent with experimentally observed reactivity trend of MoS_2−NC/Fe^0.

Overall, the updated DFT results now support the catalyst design for including both the NC and Fe^0 components: NC mediates electron delivery from Fe^0 to the Mo catalytic domain and provide a more favorable electronic environment, which

enhances ClO_4^- adsorption and supports OAT reactivity, rather than acting solely as a conductivity additive. We have revised the manuscript to clarify its role and to keep the scope of DFT appropriately mentioned. These are the corresponding updated text and figures in our revised manuscript:

Density of states (DOS) analysis for the MoS_2 slab alone confirms its semiconducting feature, with the Fermi level located within the band gap (Supplementary Fig. 2). When combining with NC, electronic states appear near the Fermi level in the total DOS. Layer-projected DOS indicates that these near-Fermi states are not solely contributed by NC, but also by MoS_2, which is attributed to the interfacial charge redistribution. Similar trends are also observed for $MoS_2|Fe^0$ and $MoS_2|NC|Fe^0$ models. To further investigate the electron migration behavior, we conducted charge density difference analysis, and quantified net electron transfer by Bader analysis. The MoS_2 layer receives 0.343 e^- (0.257 e^-/nm^2) in $MoS_2|NC$ and 0.511 e^- (0.383 e^-/nm^2) in $MoS_2|NC|Fe^0$, consistent with charge density difference plots (Figs. 1a and 1b) that show electron accumulation on MoS_2 (yellow) and depletion in the NC layer (cyan). The charge density difference plot of $MoS_2|NC|Fe^0$ relative to NC (Supplementary Fig. 3) further shows that electrons donated from Fe^0 accumulate primarily within the NC interlayer. Quantitatively, Bader analysis indicates that Fe^0 donates 3.270 e^- to NC (2.453 e^-/nm^2), of which 0.511 e^- are transferred to NC (0.383 e^-/nm^2) in $MoS_2|NC|Fe^0$. These results confirm the electron transfer from Fe to the catalytic MoS_2 domain is effectively mediated by the interlayer NC, consistent with the D−A design. In contrast, direct contact in $MoS_2|Fe^0$ yields a larger electron transfer of 4.045 e^- (3.034 e^-/nm^2) to MoS_2 (Fig. 1c), but this does not result in improved ClO_4^- activation (as discussed below), suggesting that catalytic promotion requires optimized electronic redistribution rather than simply increasing electron density of MoS_2.

We then compared the adsorption energy of ClO_4^- at undercoordinated Mo sites for the three models (Supplementary Fig. 4). Introducing Fe^0 to $MoS_2|NC$ enhances ClO_4^- adsorption, with adsorption energy decreasing from −0.368 eV to −0.461 eV (Figs. 1d and 1e). In comparison, despite the stronger electron transfer in $MoS_2|Fe^0$, ClO_4^- adsorption is not facilitated (Fig. 1f). This can be attributed to the overly electron-rich environment in $MoS_2|Fe^0$ that results in electrostatic repulsion toward the anionic oxyanion (ClO_4^-). In comparison, the NC interlayer mediates electron transfer but avoids unfavorable electrostatic effects toward an anionic substrate, providing a more suitable electronic environment for ClO_4^- binding. In addition, in all the models, ClO_4^- preferentially adsorbs at undercoordinated Mo sites over S sites. For example, the ClO_4^- adsorption energy on the S sites of $MoS_2|NC|Fe^0$ model is unfavorable (+1.917 eV).

To probe OAT reactivity, we calculated the energy barrier for the cleavage of the first Cl−O bond of adsorbed ClO_4^- for the three models (Fig. 1g). In the absence of Fe^0, $MoS_2|NC$ exhibits an energy barrier of 0.603 eV for O atom abstraction from ClO_4^-, whereas the introducing Fe^0 lowers this barrier to 0.479 eV for $MoS_2|NC|Fe^0$. By contrast, $MoS_2|Fe^0$ model exhibits the highest barrier among the three models. These results demonstrate the Mo−O bond formation coupled with Cl−O cleavage for initiating OAT was most favorable in the $MoS_2|NC|Fe^0$ configuration. We further evaluated the subsequent dissociation of the Mo-bound O atom from Mo sites, which involves the hydrogenation of O atom (*O → *OH) and the release of OH^- (*OH → * + OH^-) (Fig. 1h). In these steps, the $MoS_2|NC|Fe^0$ model shows the most favorable energetics, with the lowest ΔG for the hydrogenation step (−0.271 eV) and the total energy for the two step (0.203 eV). It should also be noted that although the dissociation energy of the O atom from Mo sites remains thermodynamically uphill for both the three systems, the overall reaction free energy accounting for both Cl−O bond cleavage and its O atom dissociation is negative, particularly in $MoS_2|NC|Fe^0$, which indicates the ClO_4^- reduction is thermodynamically favorable in the $MoS_2|NC|Fe^0$ model.

In addition, during structural optimization, in a test model constructed with less vacancies (two S removed), adsorption at the more highly coordinated Mo sites led to spontaneous cleavage of the Cl−O bond, indicating an unrealistically barrierless pathway, whereas this behavior was not observed for our selected vacancy model (three S removed). This suggests that an appropriate coordination environment is required to enable efficient ClO_4^- reduction. Taken together, these DFT

results demonstrate the functional role of the D−A configuration, where Fe^0 enables sustained electron transfer and MoS_2 provides favorable adsorption and reactive Mo sites, while NC acts as an electronic mediator that optimizes (rather than maximizes) electron transfer to the catalytic domain, enhancing ClO_4^- adsorption and promoting OAT energetics. These results provide theoretical support for the design rationale of MoS_2−NC/Fe^0 as a D−A system for efficient ClO_4^- reduction. (Lines 104-162 in the revised main text)

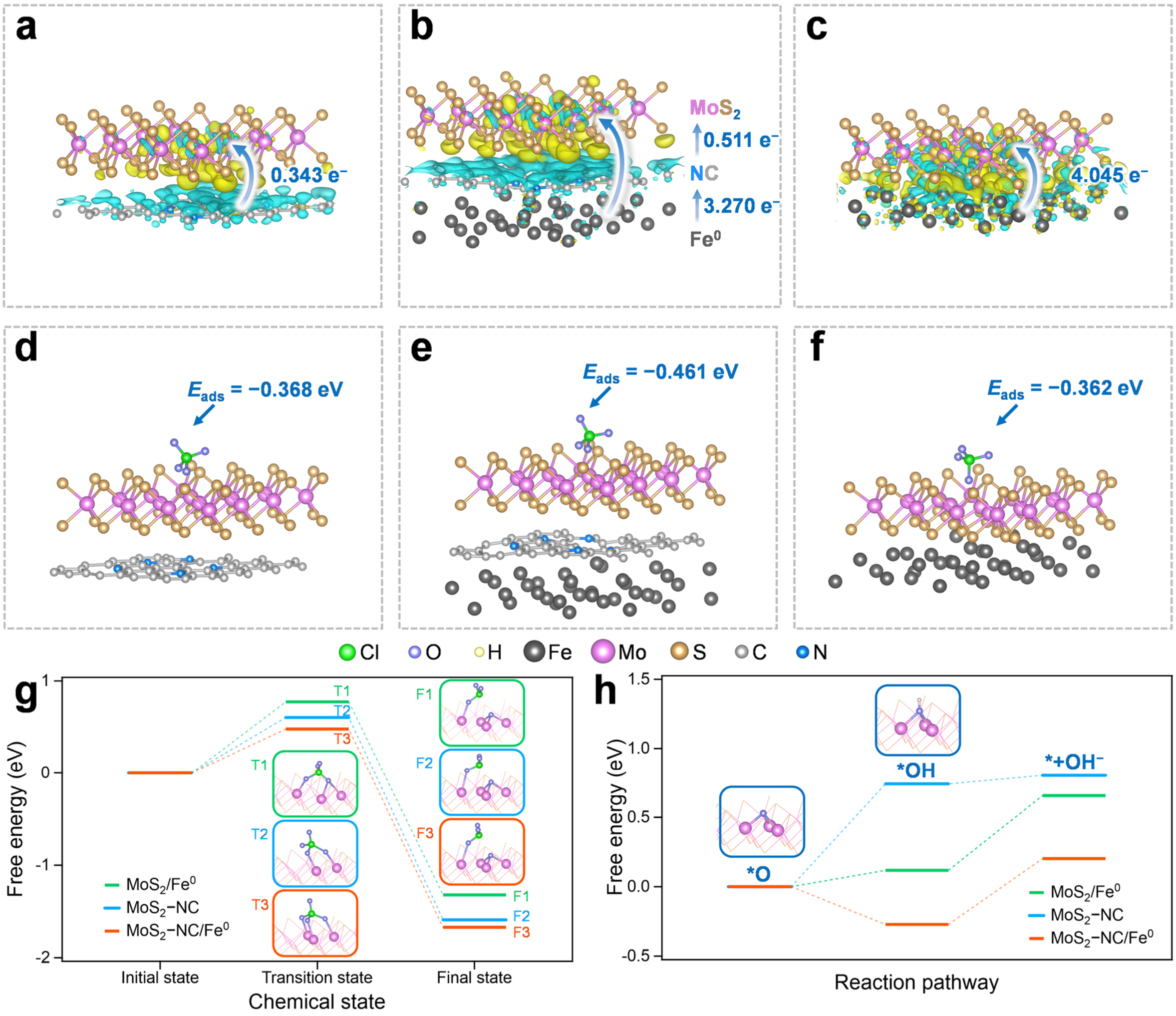

Fig. 1 | DFT calculations of MoS_2−NC/Fe^0. a-c, Charge density difference plot for (a) MoS_2|NC, (b) MoS_2|Fe^0, and (c) MoS_2|NC|Fe^0 models. d-f, Adsorption energies and configurations of ClO_4^- on (d) MoS_2|NC, (e) MoS_2|Fe^0, and (f) MoS_2|NC|Fe^0. g, Free energy barriers for first oxygen atom cleavage from adsorbed ClO_4^- on different surfaces. h, Energy diagrams for the dissociation of O atom from the Mo sites at pH 6.0. (In the revised main text)

Finally, regarding our prior work (DOI: 10.1021/acs.est.5c00564), we would like to clarify that the materials design, target contaminant, and mechanism in the present manuscript are distinct. The earlier study focused on MoS_2-modified ZVI composite for reductive dechlorination of chlorinated hydrocarbons, whereas the present work focused on ClO_4^- reduction via oxygen atom transfer pathway under mild conditions using MoS_2−NC catalyst with co-mixed Fe^0). Accordingly, the catalyst architecture

(co-mixed donor−acceptor vs MoS_2-modified ZVI). We address this distinction in more detail in our response to the next comment.

References:

(1) Liu, X.; Shi, C.; Zhang, G.; Gao, R.; Pan, L.; Zhang, X.; Huang, Z.-F.; Gong, J.; Zou, J.-J. Oxyanions stabilized low-coordinated nickel species for efficient and durable nucleophilic electrooxidation. Nat. Commun. 2025, 16 (1), 11224..

(2) Nayak, S.; Lovering, K.; Bu, W.; Uysal, A. Anions enhance rare earth adsorption at negatively charged surfaces. The Journal of Physical Chemistry Letters 2020, 11 (11), 4436-4442.

(3) Stiehler, C.; Calaza, F.; Schneider, W.-D.; Nilius, N.; Freund, H.-J. Molecular adsorption changes the quantum structure of oxide-supported gold nanoparticles: chemisorption versus physisorption. Phys. Rev. Lett. 2015, 115 (3), 036804.

2) The role of N-doped carbon requires further justification. The authors state that the N-doped carbon is necessary for MoS2 to exhibit catalytic activity (lines 336-339). However, in their previous study, they showed that ball milling of MoS2 can also provide defective sites, leading to efficient degradation of hexachloroethane in a galvanic system. It would be therefore informative to include experiments testing ball-milled MoS2 without the NC support for perchlorate reduction.

We appreciate your suggestion to further justify the role of N-doped carbon (NC).

First, while it is true that ball milling of MoS_2 can provide defective sites that enabled effective reduction of hexachloroethane in our previous study (Chen et al., 2025, DOI:10.1021/acs.est.5c00564), the material design and the reaction pathways in that work fundamentally differ from the present study. In the previous work, ZVI itself served as the primary reductant for hexachloroethane, and MoS_2 was introduced as a surface modifier to enhance the reactivity of ZVI. MoS_2 was directly combined with ZVI via ball milling to form a MoS_2-modified ZVI composite. ClO_4^- reduction under mild conditions is much more challenging and ZVI cannot achieve that. In the present system, the MoS_2−NC (the pre-fabricated catalyst) was not used to modify Fe^0, but to provide the catalytic domain. Fe^0 and MoS_2−NC are co-mixed in the solution and do not generate a single composite.

To directly evaluate the reactivity of the MoS_2-modifed ZVI (Fe^0) for ClO_4^- reduction and the role of NC, we conducted new control experiments using (i) ball-milled MoS_2 (without NC), (ii) co-milled MoS_2 and Fe^0 (MoS_2-modified ZVI as in our previous study[1]), and (iii) co-milled MoS_2 and with NC. Fe^0 was added to the reactor during reaction for (i) and (iii), but no additional Fe^0 was added for (ii) because Fe^0 is already integrated in the composite. The total Fe^0 dose in all the tests was identical to the present study (1.2 g/L). The results (Fig. R1) show that ball-milled MoS_2 and co-milled MoS_2 and Fe^0 composite (MoS_2-modified ZVI) exhibit negligible ClO_4^- removal, whereas incorporating NC (co-milled MoS_2 and NC, then mixed with Fe^0 during reaction) leads to measurable activity (~30% ClO_4^- removal over 18 h). These results confirm that while defect-rich MoS_2 can be generated via ball milling, it alone is insufficient for ClO_4^- reduction. Importantly, the prior MoS_2-modified ZVI composite does not show reactivity toward ClO_4^- and therefore does not provide the conceptual basis for the present system. Instead, the combination of NC and defect-rich MoS_2 is necessary to enable reactivity in MoS_2−NC/Fe^0, indicating a mechanism and system configuration that are distinct from our previous systems.

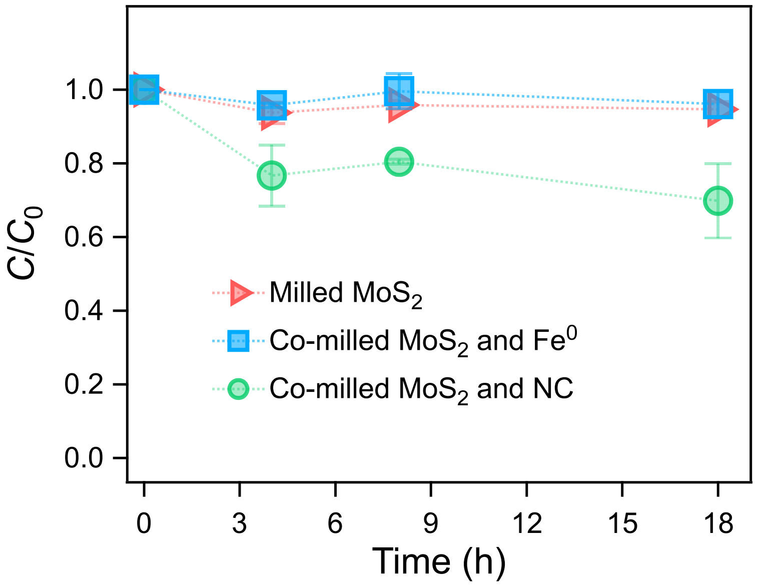

Fig. R1. ClO_4^- removal in three control systems, including (i) ball-milled MoS_2 with Fe^0 added during reaction, (ii) co-milled MoS_2 and Fe^0 (no additional Fe^0 during reaction), the similar material in our previous study,[1] and co-milled MoS_2 and NC, with Fe^0 added during reaction. Reaction conditions: $[ClO_4^-]_0$ = 1.0 mM, $[Fe^0]_0$ = 1.2 g/L, $[MoS_2]$ = 0.28 g/L, [NC] = 3.72 g/L, all the concentrations refer to the final solution, and are identical to those in Fig. 3a. pH is maintained at 6.0 with 150 mM buffer, T = 25 °C.

We have added these experimental results and the updated discussion to the revised manuscript (Supplementary Fig. 29).

> These roles are further supported by control experiments (Supplementary Fig. 29). Although ball milling can introduce defects into MoS_2, ball-milled MoS_2 (without NC) alone showed negligible ClO_4^- removal upon Fe^0 addition. In contrast, co-milling MoS_2 with NC enabled measurable ClO_4^- reduction in the presence of Fe^0, indicating that NC is required for the catalytic reactivity of defect-rich MoS_2 in this system. (Lines 373-378 in the revised main text)

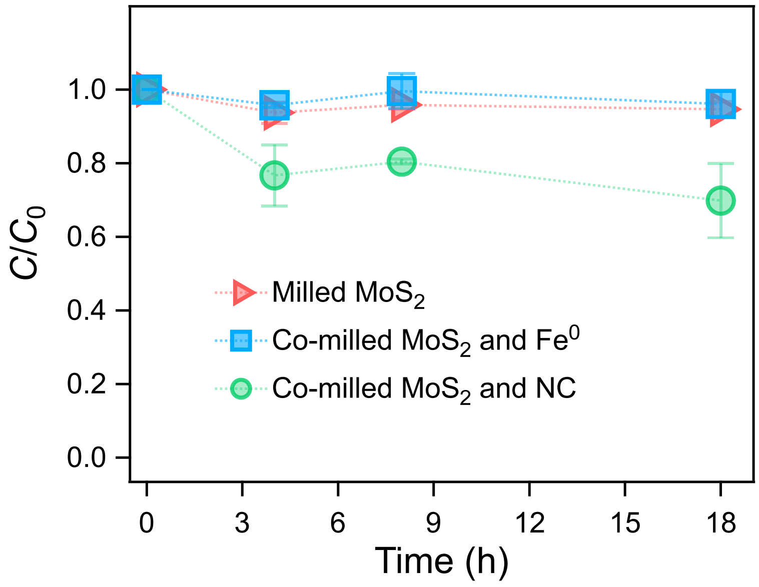

Supplementary Fig. 1. ClO_4^- removal in three control systems, including ball-milled MoS_2 with Fe^0 added during reaction, co-milled MoS_2 and Fe^0 (no additional Fe^0 during reaction), and co-milled MoS_2 and NC, with Fe^0 added during reaction. Reaction conditions: $[ClO_4^-]_0$ = 1.0 mM, $[Fe^0]_0$ = 1.2 g/L, $[MoS_2]$ = 0.28 g/L, [NC] = 3.72 g/L, all the concentrations refer to the final solution, and are identical to those in Fig. 3a. pH is maintained at 6.0 with 150 mM buffer, T = 25 °C. (In the revised Supplementary Information)

References:

(1) Chen, J.; Feng, Y.; Liu, Y.; Fan, P.; Ao, Z.; Qin, H.; Guan, X. MoS_2 modification to enhance the removal of various chlorinated hydrocarbons by zerovalent iron. Environ. Sci. Technol. 2025, 59 (22), 11330-11340.

3) The characteristics of Fe(0), such as particle size, morphology, specific surface area, and surface properties are not documented in the manuscript except for the zeta potential measurements (Figure S12). These should be studied in more detail and their effects on the perchlorate removal performance should be evaluated.

Thank you for pointing out the need for more detailed characterization of the Fe^0. In the revised manuscript, we have added detailed characterization of Fe^0 and included the results.

We have measured the particle size and Brunauer−Emmett−Teller (BET) specific surface area of Fe^0, and the result is now provided in the Method section.

The synthesized Fe^0 particles exhibited a mean particle size of 220 nm and a specific surface area of 3.74 m^2/g. (Lines 492-494 in the revised main text)

We conducted transmission electron microscopy (TEM) and high-angle annular dark-field scanning TEM (HAADF-STEM) imaging, along with elemental mapping (EDS), to examine the morphology and elemental distribution of Fe^0 (Fig. R2). These results are now presented in Supplementary Fig. 9, confirming the nanoscale morphology.

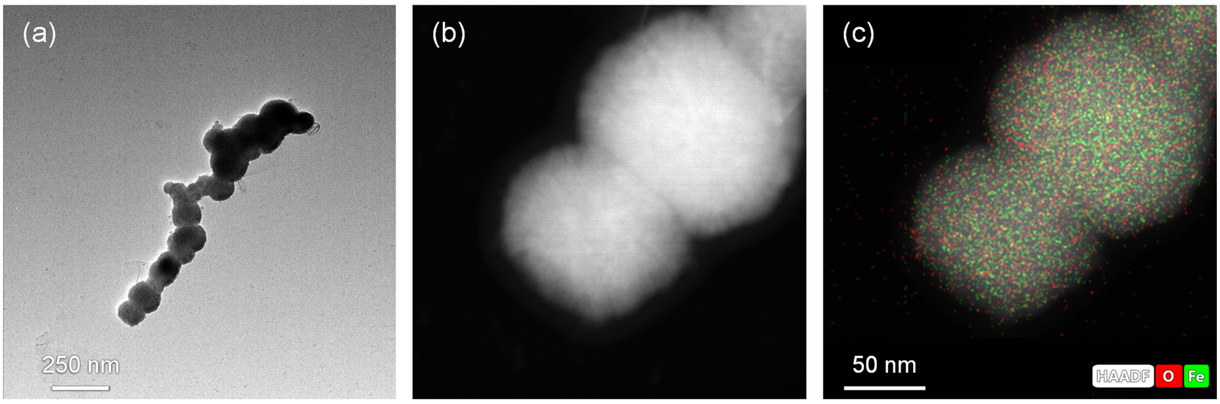

Fig. R2. (a) Transmission electron microscopy (TEM), (b) high-angle annular dark-field scanning TEM (HAADF-STEM) imaging and corresponding (c) EDS elemental mapping of Fe^0 particles.

X-ray diffraction (XRD) pattern and X-ray photoelectron spectroscopy (XPS) were performed to analyze the crystallinity and surface chemical states of Fe^0 (Fig. R3). The XRD pattern shows characteristic peaks of Fe^0, confirming successful synthesis. XPS analysis reveals the Fe(0), Fe(II), and Fe(III) species on the particle surface, indicating partial surface oxidation, which is commonly observed for nanoscale Fe^0 and is consistent with literature reports.[1-3]

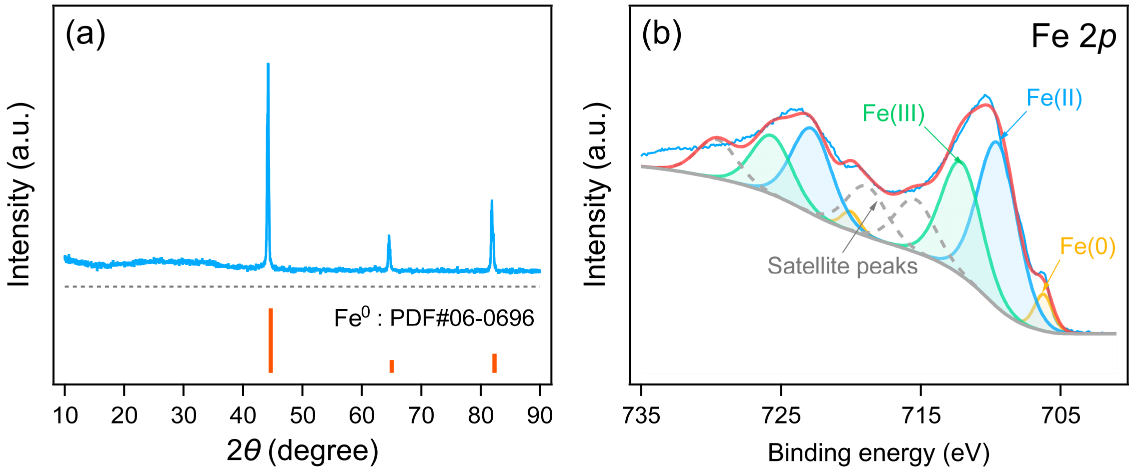

Fig. R3. (a) X-ray diffraction (XRD) pattern and (b) X-ray photoelectron spectroscopy (XPS) spectrum of Fe^0 particles used in this study.

These results are provided in Supplementary Fig. 10 (Fig. R2) and discussed in the revised main text.

> These particles were characterized by TEM, XRD, and XPS, and the corresponding results provided in the Supplementary Figs. 9 and 10, which is consistent with commonly reported nZVI properties. (Lines 235-240 in the revised main text)

In addition, the effect of surface availability of Fe^0 on ClO_4^- reduction, we synthesized Fe^0 particles with different sizes. Except for the Fe^0 used in the main

experiments (particle sizes of 220 nm), we synthesized larger Fe^0 particles using higher concentrations of ferrous sulfate (89.3 mM and 178.6 nm), obtaining mean particle sizes of 407 and 654 nm, respectively. We also tested a commercial Fe^0 (Macklin) with a particle size of 1555 nm. Their size distributions are presented in Fig. R4. Their ClO_4^- reduction kinetics in the Fe^0/MoS_2-NC system is compared (Fig. R5). The observed first-order rate constants decrease with increasing particle size, indicating that greater available surface area of Fe^0 provide faster electron supply. These results also show the reactivity remains with larger Fe^0 particles (especially with higher dose, as described later).

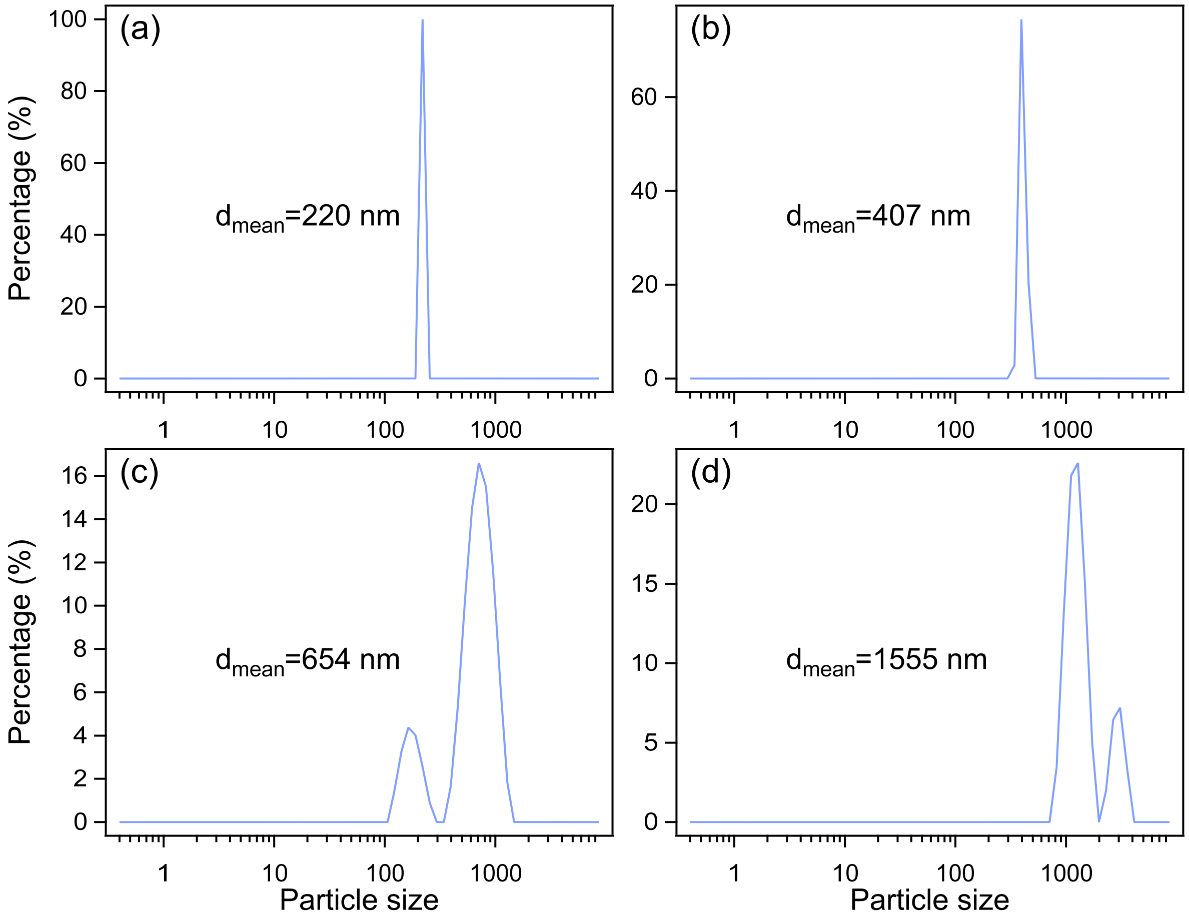

Fig. R4. Particle size distributions of four Fe^0 samples used to evaluate particle-size effects. In addition to the Fe^0 used in the main experiments (panel a, particle sizes of 220 nm), larger Fe^0 particles with mean sizes of 407 and 654 nm (panels b and c) were synthesized by increasing the concentration of ferrous sulfate to 89.3 mM and 178.6 mM, respectively. A commercial Fe^0 sample (Macklin) with a mean particle size of 1555 nm was also included for comparison (panel d).

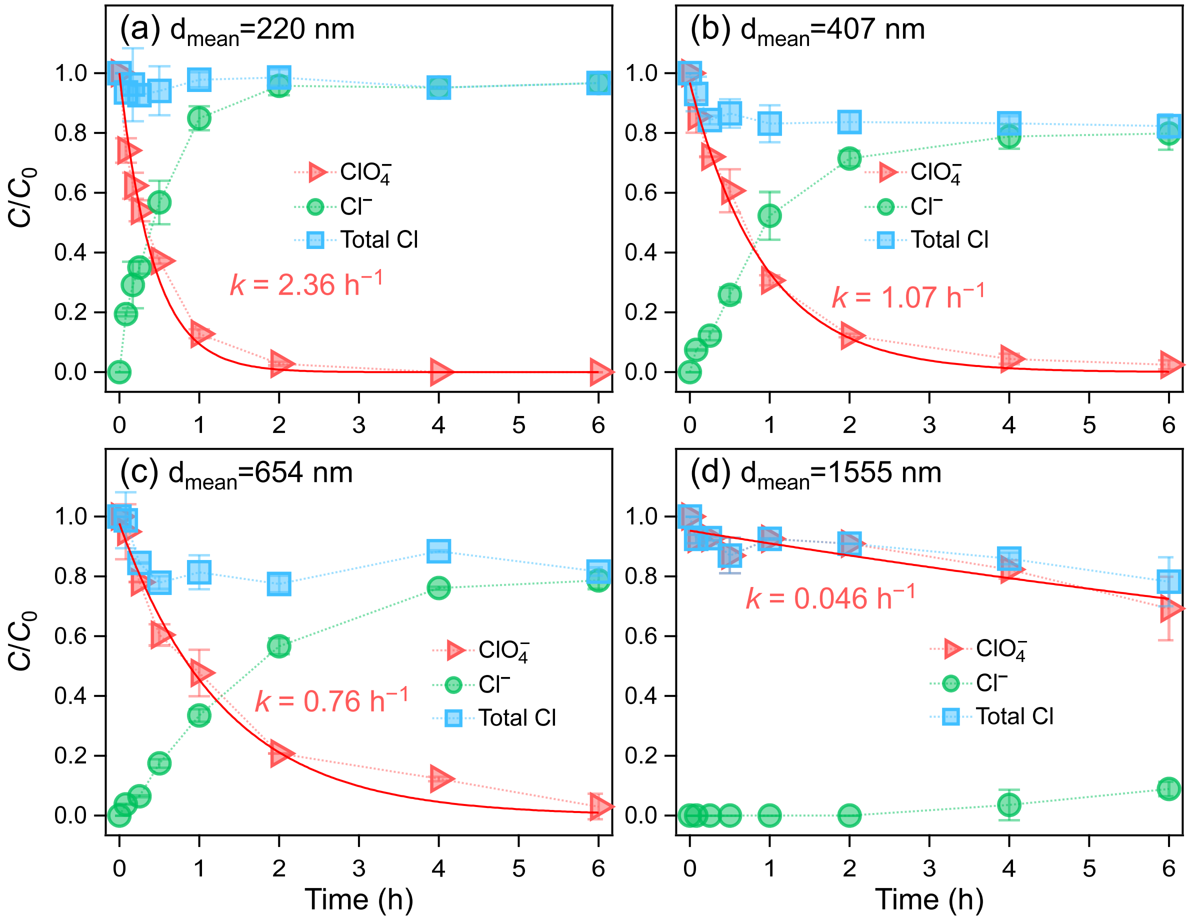

Fig. R5. Influence of particle sizes of Fe^0 on the ClO_4^- reduction in MoS_2−NC/Fe^0 systems. Data for 220 nm are those shown in Fig. 2a in the main text, and presented here for comparison. Reaction conditions: $[ClO_4^-]_0$ = 1.0 mM, $[Fe^0]_0$ = 1.2 g/L, [MoS_2−NC] = 4.0 g/L, the Mo loading is 4.2 wt.%, pH was maintained at 6.0 with 150 mM buffer, T = 25 °C. Solid lines in panels a-d are the first-order fits.

Accordingly, we added the new results (Figs. R4 and R5, presented as Supplementary Figs. 16 and 17) and corresponding discussion into the revised main text:

> In addition, varying Fe^0 particle size (220-1555 nm) showed that the ClO_4^- reduction rate decreases with increasing Fe^0 particle size (Supplementary Figs. 16 and 17). This trend suggests that Fe^0 surface availability is a critical factor for the reactivity, further supporting the role of Fe^0 as the direct electron donor in this system. (Lines 258-261 in the revised main text)

References:

(1) Liu, H.; Li, Z.; Sun, Y.; Bao, Z.; Zhou, M.; Li, J.; Guan, X. Cointercalation of zero-valent iron for improving spatiotemporal selectivity toward heavy metals in wastewater. Adv. Funct. Mater. 2026, e28568.

(2) Li, S.; Wang, W.; Liang, F.; Zhang, W.-x. Heavy metal removal using nanoscale zero-valent iron (nZVI): Theory and application. J. Hazard. Mater. 2017, 322, 163-171.

(3) Xue, W.; Shi, X.; Guo, J.; Wen, S.; Lin, W.; He, Q.; Gao, Y.; Wang, R.; Xu, Y. Affecting factors and mechanism of removing antibiotics and antibiotic resistance genes by nano zero-valent iron (nZVI) and modified nZVI: A critical review. Water Res. 2024, 253, 121309.

4) The technoeconomic analysis is flawed and misleading. It is based on unrealistic Fe(0) price (0.8 USD/kg, line 384) corresponding to bulk iron and not nZVI, which typically costs above 100 USD/kg. This would make the more conventional Pd/H2 process more economical. It is also noteworthy that the manuscript consistently avoids using the term "nano-ZVI," even though the borohydride-reduction route used is a well-established method for preparing nanoscale Fe(0). This omission gives the impression that the cost assumptions were chosen to make the proposed process appear more favorable than it realistically is.

We appreciate your attention to the TEA section. We agree that using a bulk iron price could be misleading. In the revised manuscript, we updated the TEA to use commercially relevant Fe^0 prices.

To better reflect the economic feasibility of the proposed system, we re-evaluated the performance using a commercially available coarser Fe^0 powder (20 mesh, from Thermo Fisher Scientific (Alfa Aesar product)[1,2]). Although a higher Fe^0 dose (12 g/L, 10× the nano Fe^0 dose) was required, the system still showed effective ClO_4^- reduction performance (80% ClO_4^- reduction in 18 h, Fig. R6), and could remain economically competitive due to the lower unit cost of coarser Fe^0.

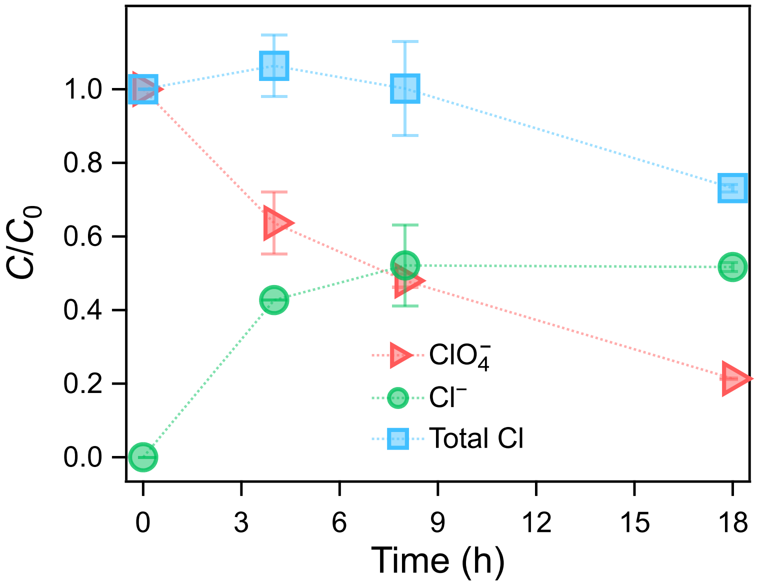

Fig. R6. ClO_4^- reduction kinetics using a coarser Fe^0 powder (20 mesh, purchased from Thermo Fisher Scientific). Reaction conditions: $[ClO_4^-]_0$ = 1.0 mM, $[Fe^0]_0$ = 12 g/L, [MoS_2−NC] = 4.0 g/L, the Mo loading is 4.2 wt.%, pH was maintained at 6.0 with 150 mM buffer, T = 25 °C.

Based on the experimental results using coarser Fe^0 powder, we recalculated the TEA based on realistic commercial prices for all the consumables on a consistent basis (the same amount of ClO_4^- removed) under continuous operation. The updated TEA supports the cost advantage for MoS_2−NC/Fe^0 over the Pd/H2-based system. The results show the cost of Pd/H_2 is dominated by catalyst-related costs, whereas the Fe-based process is dominated by Fe^0 consumption and sludge management.

The updated TEA, with the practical price and conditions of continuous operation, is provided in the Supplementary Note 1.

Cost Assumptions for Catalysts and Reagents

Based on the experimental results using coarser Fe^0 powder, we conducted TEA based on realistic commercial prices and industrial prices for both MoS_2−NC/Fe^0 and Pd/H_2-based system.

A preliminary consumables-based cost comparison was performed on a 1 L treatment basis with an initial ClO_4^- concentration of 1 mM. For the MoS_2−NC/Fe^0 system, 4 g/L of MoS_2−NC (4.2 wt.% Mo) and 12 g/L of coarser Fe^0 were applied. Under these conditions, 12 g/L Fe^0 removed 80% of 1 mM ClO_4^- in 18 h; thus, the Fe^0 cost was normalized by 1/0.8 to enable comparison on an equivalent (per 1 mM removed) basis. Using industrial bulk prices for coarser Fe^0 of \$4.4-\$10.22 per kg (from iron-filing.com, chemicalstore.com, and ScienceKitStore.com), the normalized Fe^0 cost was \$0.066-\$0.153 per L. The Mo precursor for MoS_2−NC synthesis, ammonium tetrathiomolybdate, was priced at \$18-\$33 per kg (from scrapmonster.com), contributing \$0.0082−\$0.0150 per L based on the Mo loading (4.2 wt.% Mo). The NC fraction in MoS_2−NC was estimated as ~93 wt.% (3.72 g/L). The base carbon price was taken as \$1.77-\$2.54 per kg using industrial activated carbon and carbon black references (from businessanalytiq.com, indexbox.io, businessanalytiq.com, and imarcgroup.com), and the NC price was estimated by applying a 1.5-6.5× multiplier to account for nitrogen doping and the associated yield penalty, processing energy, etc., following published cost-estimation approaches for functionalized carbons, giving an NC price of \$2.66-\$16.51 per kg. This corresponds to an NC cost contribution of \$0.0099-\$0.061 per L. Therefore, the total cost of the MoS_2−NC/Fe^0 system is \$0.084-\$0.229 per L, i.e., 21-87× lower than the Pd/H_2 comparator under the same removal basis.

For the Pd/H_2-based system, we estimated the cost of the $[(NH_2)_2$*bpy*$]MoO_x$−Pd/C system, where 0.2 g/L catalyst (5 wt.% Pd, 5 wt.% Mo) achieved complete removal of 1 mM ClO_4^-. Using bulk-pack supplier catalog prices for 5 wt.% Pd/C, the catalyst cost was estimated as \$11,450-\$17,840 per kg (from strem.com, fuelcellearth.com, srlchem.com), corresponding to \$2.30-\$3.57 per L at 0.2 g/L. The Mo precursor $Na_2MoO_4{\cdot}2H_2O$ was priced at \$13-\$15 per kg (from made-in-china.com and contributing negligibly (\$0.00033-\$0.00038 per L). The ligand $(NH_2)_2$*bpy* (assumed 1:1 molar with Mo) was priced at \$125.8-\$189.4 per g (from chemicalbook.com, biosynth.com, and tcichemicals.com), contributing \$2.44-\$3.68 per L. Therefore, the consumables cost for the Pd/H_2 system was \$4.74-\$7.25 per L without operational costs. In addition, Pd/H_2-based operation typically requires acidification to pH 3 and subsequent neutralization (estimated ~\$0.1 per L depending on alkalinity) and a continuous H_2 supply (~\$0.003 per L), resulting in a total estimated cost of \$4.84-\$7.35 per L.

All the prices used to estimate the cost were obtained from publicly available online listings for bulk-pack reagents from major suppliers and commercial distributors. Therefore, these values should be interpreted as order-of-magnitude estimates rather than absolute costs. In addition, the calculations above represent a first-run estimate, i.e., the full material inputs were charged to a single treatment run to enable a consistent comparison of the dominant cost contributors between the two systems. In continuous operation, catalyst reuse could reduce the effective cost per treated volume. We further calculated the operating expenditure below.

Operating expenditure (OPEX) comparison

For the MoS_2−NC/Fe^0 system, the OPEX includes (i) Fe^0 consumption (supplemental Fe^0 dosing), (ii) solid/sludge handling and disposal, (iii) MoS_2−NC supplementation, and (iv) utilities/maintenance. For 1 mM ClO_4^- removed per m^3 (1 mol/m^3), the stoichiometric Fe^0 demand is 0.223 kg/m^3. With an electron efficiency of 20%-40%, the net Fe^0 demand is 0.56-1.12 kg/m^3, corresponding to \$2.5-\$11.5/m^3 using the industrial Fe^0 price mentioned above. The consumed Fe is converted to $Fe(OH)_2$/$Fe(OH)_3$ solids, which requires separation, dewatering, and disposal. Using reported dewatered sludge disposal cost benchmarks (e.g., 300-800/dry ton), and kg-scale dry solids generation per m^3 (~1-2 kg dry solids per m^3, depending on $Fe(OH)_x$ form), the sludge handling/disposal cost is estimated as \$0.3-\$1.9/m^3. For MoS_2−NC supplementation, the initial catalyst input was estimated to \$18-\$76/m^3 as discussed above. The deactivation fraction per run was calculated from the half-life and run time. From the Fig. 3h, the half-life was estimated to be 3.3 days, with a run time of 18 h (Supplementary Fig. 32) and 1% catalyst lost for each run. Because each run removes only 80% of ClO_4^-, the catalyst cost per m^3 treated was normalized to report cost per 1 mM removed. The cost of the catalyst supplementation would be \$3.50-\$14.80/m^3. Finally, for utilities and maintenance, wastewater treatment energy use commonly is 0.30-1.89 kWh/m^3, while routine maintenance is site-specific but ignorable relative to material supplementation and solids handling. Given the U.S. industrial electricity price benchmark (~\$0.081/kWh), the electricity cost is \$0.02-\$0.15/m^3. Summing these terms, the estimated OPEX for MoS_2−NC/Fe^0 is ~\$6.27-\$28.25 per m^3 (for 1 mM ClO_4^- removed), dominated by Fe^0 consumption and sludge management.

For the Pd/H_2-based [$(NH_2)_2$*bpy*]MoO_x−Pd/C system, the OPEX should include (i) catalyst supplementation, (ii) H_2 supply, (iii) acidification to pH 3 and subsequent neutralization, and (iv) utilities/maintenance. On the same removal basis used above (1 mM ClO_4^- removed per m^3), the conversion of ClO_4^- to Cl^- is an 8-electron reduction, implying a stoichiometric H_2 demand of 4 mol H_2 per mol ClO_4^- (~0.008 kg H_2/m^3). Assuming 1-2× excess to cover inefficiencies, this corresponds to 0.008-0.016 kg H_2/m^3 and account for \$0.05-\$0.29/m^3 (\$6.2-18/kg H_2 from NREL Annual Technology Baseline (ATB) 2024). For pH adjustment, the chemical cost is estimated as \$0.03-\$0.11/m^3. Electricity is the same as above, which is \$0.02-\$0.15/m^3. In addition, catalyst supplementation dominates the OPEX of Pd/H_2 system. The first-run estimate of the catalyst is \$4.74-\$7.25 per L if treated as single-pass dosing (i.e., no reuse). The half-life was calculated to be 4.8 days according to the reported rate decay. Assuming 1 h hydraulic retention time (HRT, estimated with batch cycle time) and 1% of the catalyst lost for each run, the cost of catalyst supplementation would be \$75.9-\$116.2/m^3. The total cost would be estimated as \$76.0-\$116.8/m^3.

Capital expenditure (CAPEX) comparison

For the MoS_2−NC/Fe^0 system, capital costs are primarily driven by (i) reactor volume and (ii) solid separation and dewatering systems. Under the long HRT (18 h) used for the reduction process, the reactor requires large tankage, with mixers, pumps, and piping designed for slurry. Because Fe^0 is sacrificial and converts to $Fe(OH)_2$/$Fe(OH)_3$ solids during reaction, the system typically requires solid-liquid separation followed by mechanical dewatering (e.g., clarifier or thickener coupled with belt press, centrifuge, or filter press), along with sludge handling and loadout. Sludge management (including dewatering, handling, and transport interfaces) is widely identified as a major design and costing element in municipal and industrial wastewater facilities, and thus is expected to be a dominant CAPEX contributor for Fe^0-based treatment. Overall, this system has moderate mechanical complexity (no combustible gas system), but high installed cost sensitivity to reactor volume and sludge management.

For the [$(NH_2)_2$*bpy*]MoO_x−Pd/C system, A gas-liquid reactor is generally required, which can be packed bed, trickle bed, or stirred reactor with gas sparging. Because H_2 delivery to the liquid phase can be rate-limiting, it requires facilities to enhance mass transfer, like fine-bubble gas diffusion and mild pressurization, which increases mechanical and controls complexity relative to an all-liquid system. The core CAPEX item is the hydrogen supply and safety infrastructure, including H_2 storage (gas container or onsite supply), regulation and piping, and safety systems (hydrogen detection, alarms/interlocks, and ventilation) to prevent accumulation of flammable mixtures.

Meanwhile, the acidification and subsequent neutralization generally requires chemical storage tanks, metering pumps, mixers. Overall CAPEX implication: Lower tank volume (shorter HRT) can reduce reactor civil costs, but the hydrogen supply system with safety system and pH adjustment tends to push CAPEX to moderate-high, and it is more permitting-/hazard-driven than the Fe^0 system. Consequently, although shorter HRT can reduce reactor costs, the hydrogen supply and safety systems and pH-adjustment infrastructure lead to overall moderate-high CAPEX.

Overall, the Fe^0-based system tends to have CAPEX dominated by reactor volume and solids management, whereas the Pd/H_2 is dominated by hydrogen supply and safety systems and pH adjust infrastructure. Practically, this implies that Fe^0 systems often have lower hazard-driven CAPEX but higher solids-management CAPEX, while Pd/H_2 systems are the opposite due to hydrogen handling and safety compliance.

CAPEX contributor table for the two systems

Category	MoS_2–NC/Fe^0 system	Pd/H_2-based system	Notes
Reactor	Large volume (long HRT)	Smaller volume (short HRT)	
Gas supply system	None	H2 storage, regulation and piping	H_2 infrastructure is a distinct capital item (gas supply, controls, venting).
Hydrogen safety system	None	Detectors, alarms/interlocks, ventilation	H_2 facilities typically require detector-triggered shutdown/alarms and ventilation facilities to keep H_2 below flammability thresholds.
Solid separation	Required	Required for catalyst recycle	Fe^0 generates solids continuously, so solid-liquid separation is necessary. Pd/C may need filtration if fines need to be retained for recycling.
Dewatering equipment	Required	None	Mechanical dewatering is often a major cost contributor.
Sludge handling facilities	Required	None	$Fe(OH)_x$ solids require handling space and equipment.
Acid/base storage and dosing system	Optional	Required for acidification and neutralization	Pd/H_2 at pH 3 needs acid and base feed systems (tanks, mixers, metering pumps)
Materials of construction	Corrosion considerations	Acid and H_2 compatibility	Fe slurries require wear-resistant design; Pd/H_2 requires acid-resistant wetted parts and H_2-safe components.
Instrumentation and controls (I&C)	Standard water controls	Enhanced safety for I&C	Pd/H_2 typically requires additional safety devices (detectors, control system, hardware/software for H_2 ventilation status)

(Lines 309-414 in the revised Supplementary Information)

We also updated the TEA section in the revised main text accordingly:

Technoeconomic analysis for the MoS_2−NC/Fe^0 system

A preliminary technoeconomic analysis (TEA) was conducted to compare the MoS_2−NC/Fe^0 system with representative Pd/H_2-based catalytic systems for ClO_4^- reduction. They share a similar conceptual process design (fixed-bed or slurry contactor followed by solids separation), making the Pd/H_2-based systems the most representative comparator among abiotic ClO_4^- reduction approaches. In Pd/H_2-based systems, the typical catalyst loading is 0.2 g/L containing 5 wt.% Pd and 5 wt.% Re or Mo, operating under 1 atm H_2 at pH 3. For the MoS_2−NC/Fe^0 system, we evaluated a coarser Fe^0 powder (20 mesh) to reflect practical cost considerations. At 4.0 g/L MoS_2−NC (4.2 wt.% Mo) with 12 g/L Fe^0, the system achieved ~80% ClO_4^- removal in 18 h (Supplementary Fig. 33), and all costs were normalized to an equivalent basis (per 1 mM removed), and the detailed TEA is provided in the Supplementary Note 1.

On a 1 L treatment basis (1 mM initial ClO_4^-), a consumables-based evaluation using bulk commercial pricing yields a total materials cost of \$0.084-\$0.229 per L for MoS_2−NC/Fe^0, versus \$4.84-\$7.35 per L for the typical Pd/H_2-based comparator when including typical acidification/neutralization and H_2 supply. The cost of MoS_2−NC/Fe^0 is dominated by Fe^0 and the N-doped carbon fraction, while the Pd/H_2-based system cost is dominated by noble-metal catalyst and required ligand. Since these values represent a first-run estimate (i.e., full material inputs charged to a single treatment run), we further extended the analysis to operating expenditure (OPEX) for continuous operation on a 1 mM removed per m^3 basis. Under the assumptions described in the Supplementary Information, MoS_2−NC/Fe^0 is estimated at \$6.27-\$28.25 per m^3, dominated by net Fe^0 consumption (Fe^0 supplementation) and slurry management, whereas Pd/H_2 is estimated at \$76.0-\$116.8 per m^3, dominated by catalyst supplementation, with H_2 and pH-control costs. It should be noted that these estimates should be interpreted as order-of-magnitude benchmarks, instead of absolute values, because pricing and operating conditions are site-specific and the calculations adopt conservative assumptions on losses and deactivation, but the results consistently indicate a cost advantage for MoS_2−NC/Fe^0 under practical pricing.

From a capital expenditure (CAPEX) perspective, the MoS_2−NC/Fe^0 system is expected to be driven mainly by reactor volume (long hydraulic retention time (HRT)) and solids separation/dewatering infrastructure. In comparison, the Pd/H_2-based system has the advantage of shorter HRT but requires H_2 supply and safety systems to prevent the accumulation of flammable H_2 mixtures, which increase mechanical and permitting complexity. In addition, the acid/base storage and dosing infrastructure contributes substantially to the CAPEX. Overall, the MoS_2−NC/Fe^0 system offers advantages in safety and operational simplicity (near-neutral pH, no combustible gas), supporting its potential for cost-effective and scalable ClO_4^- treatment. In future studies, the Fe^0 could be replaced by an electrochemical cathode as the electron donor, which could further increase the cost-effectiveness, by reducing Fe^0 consumption, solid generation, and the associated slurry management. (Lines 421-457 in the revised main text)

Regarding the use of the term “nano-ZVI,” we would like to clarify that we did not intentionally avoid the term for cost reasons. Rather, we used “Fe^0” as a chemical notation consistent with the MoS_2−NC/Fe^0 formulation and maintain clarity for a multidisciplinary readership, who may be less familiar with environmental engineering terms like “nZVI” or “ZVI”. Nevertheless, to prevent misunderstanding, we have revised the manuscript to clarify the Fe^0 used in the main study was synthesized via borohydride reduction and falls within the nanoscale size range, and the term “nZVI” is now used in appropriate contexts.

The Fe^0 used here was synthesized via borohydride reduction, resulting in nanoscale zerovalent iron (known as nZVI) particles. These particles were characterized by TEM, XRD, and XPS, and the corresponding results provided in the Supplementary Figs. 9 and 10, which is consistent with commonly reported nZVI properties. (Lines 234-238 in the revised main text)

References:

(1) Perini, J. A. d. L.; Silva, B. F.; Nogueira, R. F. P. Zero-valent iron mediated degradation of ciprofloxacin – Assessment of adsorption, operational parameters and degradation products. Chemosphere. 2014, 117, 345-352.

(2) de Lima Perini, J. A.; Nogueira, R. F. P. Effect of particle size, iron ligands and anions on ciprofloxacin degradation in zero-valent iron process: application to sewage treatment plant effluent. J. Chem. Technol. Biotechnol. 2017, 92 (9), 2300-2308.

5) I would suggest moving the nZVI synthesis description into the material synthesis part in the methodology section and acknowledging the fact that it is nanoscale Fe(0).

Thank you for this suggestion. In the revised manuscript, we have moved the description of nZVI synthesis into the material synthesis section. We now clearly acknowledge that the Fe^0 was synthesized via borohydride reduction method, which yields nanoscale Fe^0 particles. The revised text:

> Fe^0 was prepared via the conventional borohydride-reduction method, which yields nanoscale Fe^0 particles. Briefly, a sodium borohydride solution was added dropwise to 100 mL of 17.86 mM ferrous sulfate solution under vigorous stirring. The amount of borohydride was twice the stoichiometric requirement for the complete reduction of ferrous ion to Fe^0. Then the Fe^0 particles were separated by vacuum filtration and washed thoroughly with DO/DI water to remove impurities in the solution. Finally, the particles were re-dispersed in DO/DI water to prepare Fe^0 suspensions for further use. All these procedures were conducted in the anaerobic chamber. The synthesized Fe^0 particles exhibited a mean particle size of 220 nm and a specific surface area of 3.74 m^2/g. These particles were further characterized by TEM, XRD, and XPS, with the corresponding results provided in the Supplementary Information. (Lines 486-494 in the revised main text)

6) To improve the cost efficiency of the described process, I would also suggest the authors to conduct experiments with coarser (and hence cheaper) Fe(0) materials.

Thanks for this comment. As suggested, we conducted additional experiments using a coarser Fe^0 powder (20 mesh, purchased from Thermo Fisher Scientific), as discussed above. Due to the lower specific surface area of the coarser Fe^0 powder, we increased the Fe^0 dose to 12 g/L (10× the nanoscale Fe^0 dose used in our main study). Despite the lower reactivity when using coarser Fe^0, the MoS_2−NC/Fe^0 system still exhibited effective ClO_4^- reduction performance (Fig. R6 above, and Supplementary Fig. 33), demonstrating the feasibility of using less expensive Fe^0 sources.

We have added the new results in the Supplementary Information (Supplementary Fig. 33) and discussed their implications in the revised technoeconomic analysis, which shows that the process can remain economically competitive even with increased Fe^0 dosing, due to the much lower unit cost of coarser Fe^0.

> For the MoS_2−NC/Fe^0 system, we evaluated a coarser Fe^0 powder (20 mesh) to reflect practical cost considerations. At 4.0 g/L MoS_2−NC (4.2 wt.% Mo) with 12 g/L Fe^0, the system achieved ~80% ClO_4^- removal in 18 h (Supplementary Fig. 33). (Lines 427-430 in the revised main text)

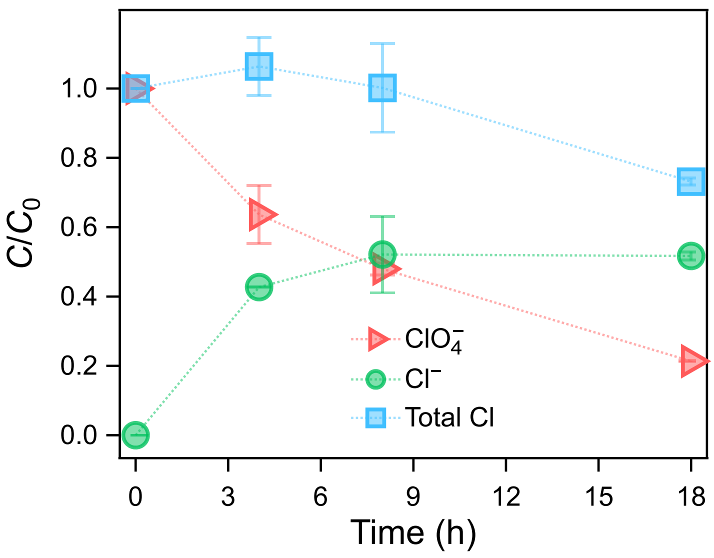

Supplementary Fig. 33. ClO_4^- reduction kinetics using a coarser Fe^0 powder (20 mesh). Reaction conditions: $[ClO_4^-]_0$ = 1.0 mM, $[Fe^0]_0$ = 12 g/L, [MoS_2−NC] = 4.0 g/L, the Mo loading is 4.2 wt.%, pH was maintained at 6.0 with 150 mM buffer, T = 25 °C. (In the revised Supplementary Information)

7) More information should be provided in the methodology section: how were the transition states and Gibbs free energies calculated, what were the operating voltages of electron microscopes, and description of zeta potential, XRD, and Raman analyses.

Thanks for your advice. In the revised manuscript, we have added the following clarifications in the Method section:

Characterization. SEM images were acquired using a Zeiss SIGMA HD electron microscope operated at an accelerating voltage of 5.0 kV with an InLens detector and a working distance of 7.4 mm. Energy-dispersive X-ray spectroscopy (EDS) was performed using an Oxford Instruments X-Max detector under an accelerating voltage of 15.0 kV. For SEM/EDS analyses, the catalyst samples were ultrasonically dispersed in ethanol, drop-cast onto clean Si wafers, and dried at room temperature. Transmission electron microscopy (TEM), high-angle annular dark-field scanning TEM (HAADF-STEM), and EDS elemental mapping of Fe^0 particles were carried out using a FEI Talos F200x microscope operated at accelerating voltage of 200 kV and equipped with a Super-X EDS system. For MoS_2−NC, STEM was performed using a JEOL JEM-2100F operated at 200 kV. All TEM samples were prepared by ultrasonic dispersion in ethanol, drop-casting onto carbon-coated copper grids, and air drying.

X-ray diffraction (XRD) patterns were recorded on a Rigaku DXR-8000 diffractometer using Cu Kα radiation (40 kV, 40 mA) with a diffracted-beam graphite monochromator. Data were recorded over 2θ of 5°-90° with a step size of 0.01° and a scan rate of 5°/min. X-ray photoelectron spectroscopy (XPS) was conducted using a Thermo Scientific K-Alpha+ instrument equipped with a monochromated Al Kα X-ray source (1486.6 eV, 12 kV). High-resolution spectra were collected with a pass energy of 50 eV and a step size of 0.1 eV. Raman spectra were collected on a LabRAM HR Evolution confocal Raman microscope (Horiba) with a 514 nm excitation laser, and the spectral range was 50-500 cm^{-1}. Zeta potentials were measured on a Zetasizer NANO-ZS (Malvern Panalytical) using electrophoretic light scattering (laser Doppler micro-electrophoresis, M3-PALS). Samples were dispersed in the same buffer solutions used in the batch experiments to maintain pH (provided below), and the suspension was loaded into a disposable folded capillary cell (DTS1070) using a syringe. (Lines 512-531 in the revised main text)

Reviewer #2

General Comments:

The manuscript reports a MoS_2–NC/Fe^0 donor–acceptor system capable of rapidly reducing ClO_4^- under mild conditions. The concept is compelling, and the experimental performance together with the comprehensive characterization provides a strong foundation for the study. The mechanistic interpretation, particularly the electronic role of the NC support and the DFT analysis, would benefit from further clarification and more quantitative support. Certain aspects of the computational modeling and technoeconomic comparison appear simplified relative to the complexity of the proposed mechanism. Addressing the points below would substantially strengthen the mechanistic rigor and overall clarity of the work. I recommend major revision, and several main concerns are discussed below.

Thank you for your comments. We appreciate your concern on the mechanistic interpretation, computational modeling, and TEA. In the revised manuscript, we made major changes to address these points. We re-conducted the DFT analysis using updated slab models that represent the donor−mediator−acceptor configuration (MoS_2|NC|Fe^0) and compared the results with MoS_2|Fe^0 and MoS_2|NC. We also added new control experiments to strengthen the mechanistic discussion. to address the economics concern, we expanded the TEA to include key OPEX/CAPEX contributors. We believe these revisions strengthen the mechanistic rigor and overall clarity, consistent with your recommendations. Below are our detailed responses.

Specific Comments:

1. The DFT model used appears inconsistent with the proposed mechanism, failing to provide any evidence to support the core argument that NC is the key electronic mediator. Figure 1B shows almost no electron transfer between NC and MoS2, indicating a weak physical interaction between them. The authors should address this inconsistency, for example, by discussing the limitations of the model or providing computational results that more directly reflect the proposed mechanism.

Thank you. We agree that the originally presented DFT results did not sufficiently support the proposed role of the NC layer as an electronic mediator. In response, we have re-conducted the DFT calculations using revised models and clarified the mechanistic interpretation accordingly.

We constructed an updated slab model representing MoS_2|NC|Fe^0 and compared it with other models (MoS_2|Fe^0 and MoS_2|NC). The updated model of MoS_2|NC|Fe^0 reflects the donor-mediator-acceptor structure. The revised calculations show that electrons are transferred from Fe^0 to the MoS_2 slab in MoS_2|NC|Fe^0 as well as in the MoS_2|Fe^0 model. Bader charge analysis indicates that the MoS_2 layer in the MoS_2|NC|Fe^0 receives a larger net electron transfer (0.511 e^-) than in MoS_2|NC (0.343 e^-), while MoS_2 layer in MoS_2|Fe^0 shows the largest electron accumulation (4.045 e^-). These trends are consistent with the charge density difference plots, showing that electrons from Fe^0 can be transferred through NC layer (Fig. R7). The charge density difference plot of MoS_2|NC|Fe^0 relative to MoS_2 shows the electron

accumulation on MoS_2 (yellow) and depletion in the NC layer (cyan). The plot relative to NC shows the electrons donated from Fe^0 accumulate primarily within the NC interlayer.

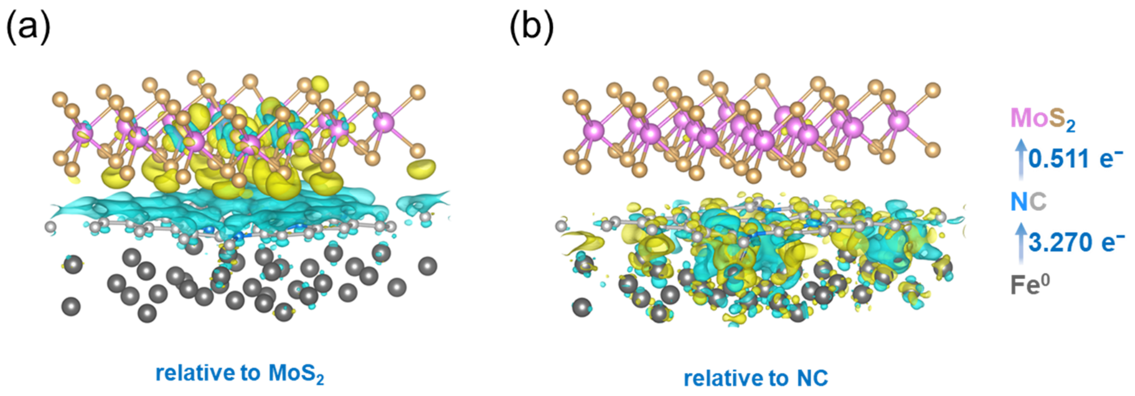

Fig. R7. Charge density difference plots of $MoS_2|NC|Fe^0$ relative to (a) MoS_2 and (b) NC.

Importantly, the larger electron transfer does not result in better catalytic performance. Despite the larger electron donation in $MoS_2|Fe^0$, this model exhibits weaker ClO_4^- adsorption and less favorable energetics for key activation steps than $MoS_2|NC|Fe^0$. This suggests that the electron-enriched MoS_2 surface in $MoS_2|Fe^0$ can increase electrostatic repulsion toward the anionic oxyanion (ClO_4^-),[1-3] thereby disfavoring the binding and subsequent O-atom transfer steps, despite the larger net charge transfer.

In contrast, the presence of the NC layer in $MoS_2|NC|Fe^0$ promotes a more favorable electronic environment for oxyanion binding at the surface: (i) more favorable ClO_4^- adsorption energies and (ii) lower free-energy barriers for the first Cl−O bond cleavage and free-energy changes for subsequent hydrogenation of Mo-bound O atoms. These effects, rather than a simple increase in electron transfer, explain the superior catalytic performance observed experimentally.

Accordingly, we have revised the manuscript to clarify that the role of NC is not to maximize electron transfer, but to modulate and optimize electronic environment at the catalytic Mo sites, thereby facilitating substrate activation and oxygen atom transfer. The updated DFT results and their interpretation are now presented in the revised Fig. 1, and Supplementary Fig. 4 discussed in detail in the main text and Supplementary Information.

> To further investigate the electron migration behavior, we conducted charge density difference analysis, and quantified net electron transfer by Bader analysis. The MoS_2 layer receives 0.343 e^- (0.257 e^-/nm^2) in $MoS_2|NC$ and 0.511 e^- (0.383 e^-/nm^2) in $MoS_2|NC|Fe^0$, consistent with charge density difference plots (Figs. 1a and 1b) that show electron accumulation on MoS_2 (yellow) and depletion in the NC layer (cyan). The charge density difference plot of $MoS_2|NC|Fe^0$ relative to NC (Supplementary Fig. 3) further shows that electrons donated from Fe^0 accumulate primarily within the NC interlayer. Quantitatively, Bader analysis indicates that Fe^0 donates 3.270 e^- to NC (2.453 e^-/nm^2), of which 0.511 e^- are transferred to NC (0.383 e^-/nm^2) in $MoS_2|NC|Fe^0$. These results confirm the electron transfer from Fe to the catalytic MoS_2 domain is effectively mediated by the interlayer NC, consistent with the D−A design. In contrast, direct contact in $MoS_2|Fe^0$ yields a larger

electron transfer of 4.045 e^- (3.034 e^-/nm^2) to MoS_2 (Fig. 1c), but this does not result in improved ClO_4^- activation (as discussed below), suggesting that catalytic promotion requires optimized electronic redistribution rather than simply increasing electron density of MoS_2.

We then compared the adsorption energy of ClO_4^- at undercoordinated Mo sites for the three models (Supplementary Fig. 4). Introducing Fe^0 to MoS_2|NC enhances ClO_4^- adsorption, with adsorption energy decreasing from −0.368 eV to −0.461 eV (Figs. 1d and 1e). In comparison, despite the stronger electron transfer in $MoS_2|Fe^0$, ClO_4^- adsorption is not facilitated (Fig. 1f). This can be attributed to the overly electron-rich environment in $MoS_2|Fe^0$ that results in electrostatic repulsion toward the anionic oxyanion (ClO_4^-). In comparison, the NC interlayer mediates electron transfer but avoids unfavorable electrostatic effects toward an anionic substrate, providing a more suitable electronic environment for ClO_4^- binding. In addition, in all the models, ClO_4^- preferentially adsorbs at undercoordinated Mo sites over S sites. For example, the ClO_4^- adsorption energy on the S sites of $MoS_2|NC|Fe^0$ model is unfavorable (+1.917 eV).

To probe OAT reactivity, we calculated the energy barrier for the cleavage of the first Cl−O bond of adsorbed ClO_4^- for the three models (Fig. 1g). In the absence of Fe^0, MoS_2|NC exhibits an energy barrier of 0.603 eV for O atom abstraction from ClO_4^-, whereas the introducing Fe^0 lowers this barrier to 0.479 eV for $MoS_2|NC|Fe^0$. By contrast, $MoS_2|Fe^0$ model exhibits the highest barrier among the three models. These results demonstrate the Mo−O bond formation coupled with Cl−O cleavage for initiating OAT was most favorable in the $MoS_2|NC|Fe^0$ configuration. We further evaluated the subsequent dissociation of the Mo-bound O atom from Mo sites, which involves the hydrogenation of O atom (*O → *OH) and the release of OH^- (*OH → * + OH^-) (Fig. 1h). In these steps, the $MoS_2|NC|Fe^0$ model shows the most favorable energetics, with the lowest ΔG for the hydrogenation step (−0.271 eV) and the total energy for the two step (0.203 eV). It should also be noted that although the dissociation energy of the O atom from Mo sites remains thermodynamically uphill for both the three systems, the overall reaction free energy accounting for both Cl−O bond cleavage and its O atom dissociation is negative, particularly in $MoS_2|NC|Fe^0$, which indicates the ClO_4^- reduction is thermodynamically favorable in the $MoS_2|NC|Fe^0$ model. (Lines 114-151 in the revised main text)

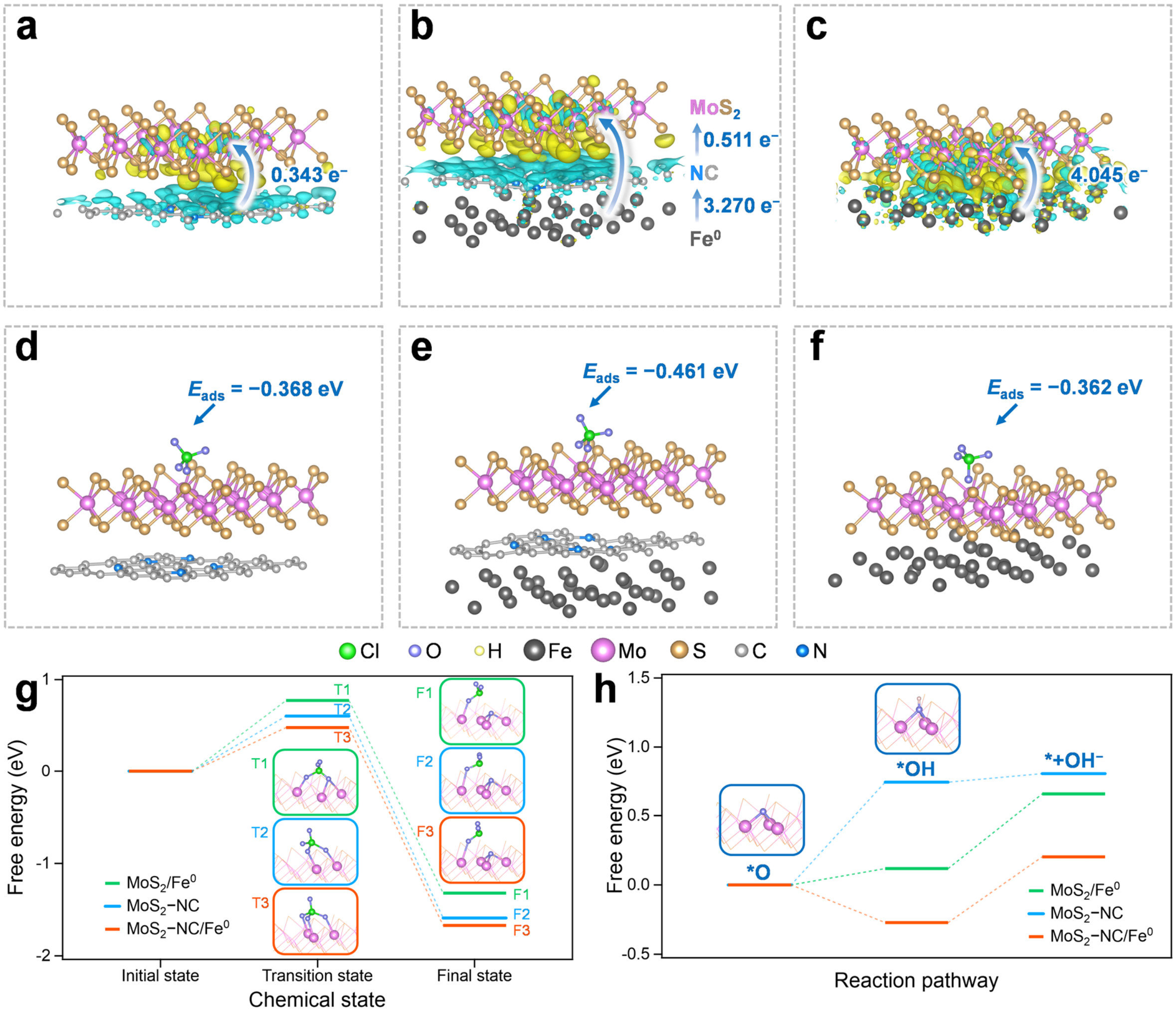

Fig. 1 | DFT calculations of MoS_2−NC/Fe^0. a-c, Charge density difference plot for (a) MoS_2|NC, (b) MoS_2|Fe^0, and (c) MoS_2|NC|Fe^0 models. d-f, Adsorption energies and configurations of ClO_4^- on (d) MoS_2|NC, (e) MoS_2|Fe^0, and (f) MoS_2|NC|Fe^0. g, Free energy barriers for first oxygen atom cleavage from adsorbed ClO_4^- on different surfaces. h, Energy diagrams for the dissociation of O atom from the Mo sites at pH 6.0. (In the revised main text)

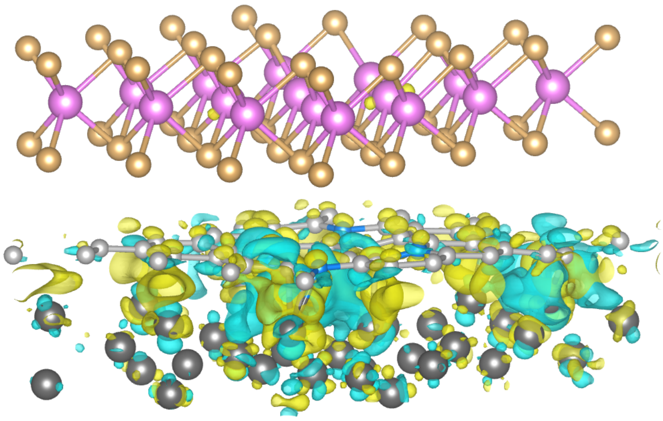

Supplementary Fig. 3. Charge density difference (CDD) plot of MoS_2|NC|Fe^0 relative to NC. (In the revised Supplementary Information)

References:

(1) Liu, X.; Shi, C.; Zhang, G.; Gao, R.; Pan, L.; Zhang, X.; Huang, Z.-F.; Gong, J.; Zou, J.-J. Oxyanions stabilized low-coordinated nickel species for efficient and durable nucleophilic electrooxidation. Nat. Commun. 2025, 16 (1), 11224..

(2) Nayak, S.; Lovering, K.; Bu, W.; Uysal, A. Anions enhance rare earth adsorption at negatively charged surfaces. The Journal of Physical Chemistry Letters 2020, 11 (11), 4436-4442.

(3) Stiehler, C.; Calaza, F.; Schneider, W.-D.; Nilius, N.; Freund, H.-J. Molecular adsorption changes the quantum structure of oxide-supported gold nanoparticles: chemisorption versus physisorption. Phys. Rev. Lett. 2015, 115 (3), 036804.

2. The claim that NC "introduces additional electronic states" to enhance conductivity lacks a proper comparative basis. The intrinsic conductivity of Fe is far superior to that of NC, and the DOS of bulk Fe is much higher at the Fermi level. To be substantiated, this would require a comparison of the electronic structures of an isolated defective MoS2 system against the composite MoS2-NC system, which is currently absent.

Thank you for this insightful comment. We agree that the original DOS results could be misleading. We have added DFT calculations of an isolated defective MoS_2 system to enable direct comparison with the MoS_2−NC composite system (MoS_2|NC model) for the DOS. The DOS analysis shows that the MoS_2|NC model exhibits enhanced electronic states near the Fermi level compared to MoS_2 alone (Fig. R8). Layer-projected DOS indicates that these near-Fermi states of MoS_2|NC are not solely contributed by NC, but also by MoS_2, which is attributed to the interfacial charge redistribution.

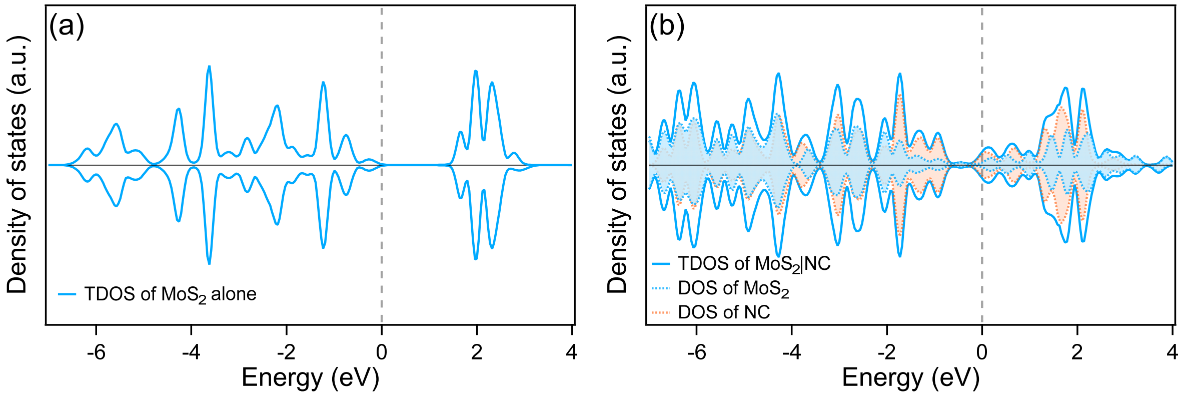

Fig. R8. Density of states (DOS) for (a) MoS_2 alone, (b) MoS_2|NC, with the layer-projected DOS shown for the contributions from MoS_2, and NC.

This result, along with the updated DFT results confirm that NC allows electron transfer through NC to MoS_2. In addition, NC plays a critical role in modulating electronic structure of MoS_2, enhancing its catalytic properties via improved adsorption and activation of ClO_4^-, as supported by the revised adsorption energy and energy barrier data (see Response to Comment 1). Although Fe^0 indeed has a much higher DOS at the Fermi level than NC, but the role of NC is not solely to increase the conductivity.

Accordingly, we added these results (Supplementary Fig. 2) and revised our manuscript:

> Density of states (DOS) analysis for the MoS_2 slab alone confirms its semiconducting feature, with the Fermi level located within the band gap (Supplementary Fig. 2). When combining with NC, electronic states appear near the Fermi level in the total DOS. Layer-projected DOS indicates that these near-Fermi states are not solely contributed by NC, but also by MoS_2, which is attributed to the interfacial charge redistribution. Similar trends are also observed for MoS_2|Fe^0 and MoS_2|NC|Fe^0 models. (Lines 109-114 in the revised main text)

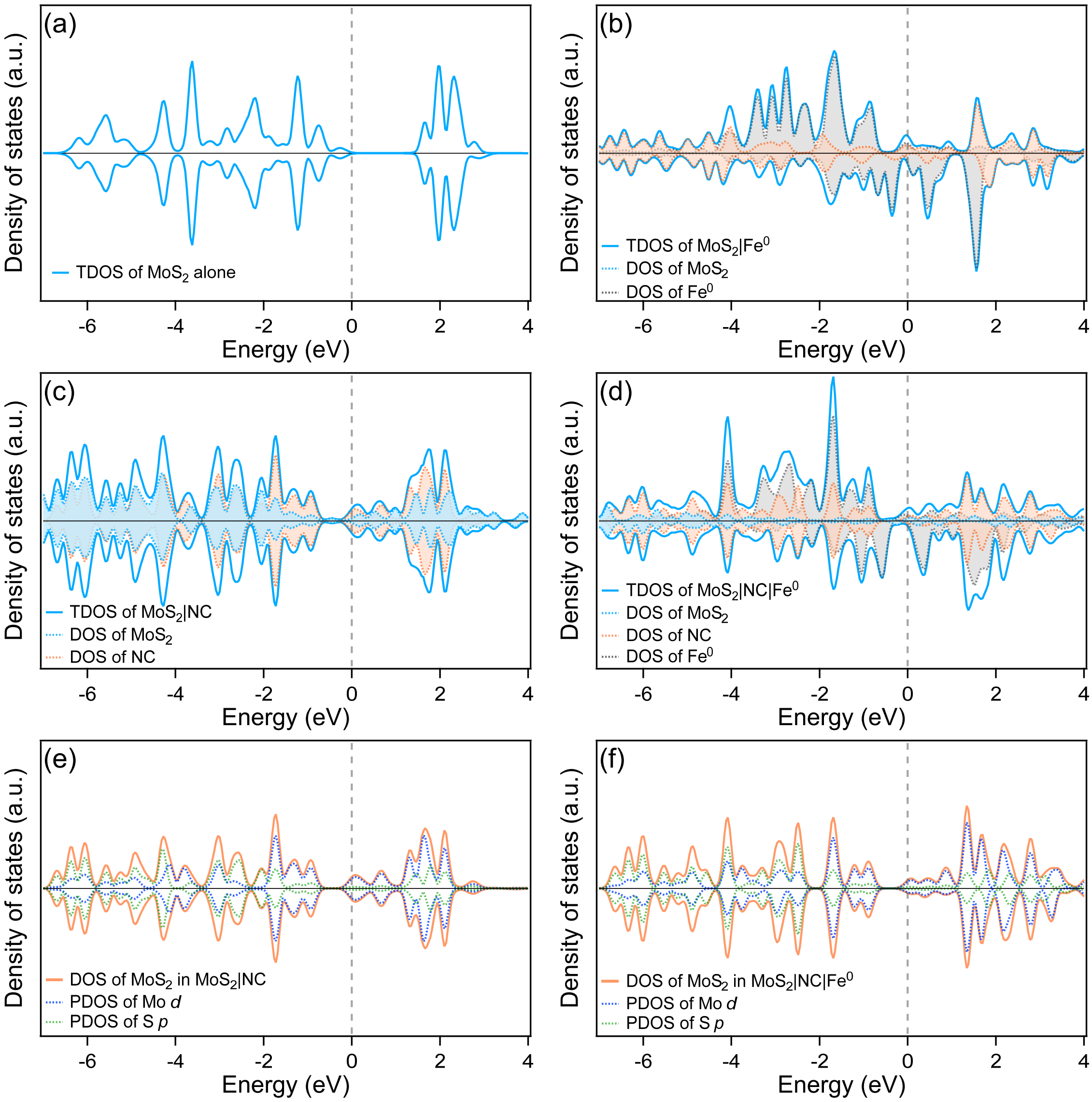

Supplementary Fig. 2. Density of states (DOS) for (a) MoS_2 alone, (b) $MoS_2|Fe^0$, (c) $MoS_2|NC$, (d) $MoS_2|NC|Fe^0$, with the layer-projected DOS shown for the contributions from MoS_2, NC, Fe^0 where applicable. (e, f) Projected DOS (PDOS) of MoS_2 in (e) $MoS_2|NC$ and (f) $MoS_2|NC|Fe^0$, in Mo *d* and S *p* orbits. Compared with MoS_2 alone, electronic states appear near the Fermi level (vertical dashed line) in $MoS_2|NC$, $MoS_2|NC|Fe^0$, which is not only contributed by NC, but also due to the MoS_2, indicating electron redistribution between the layers. For $MoS_2|NC$, $MoS_2|NC|Fe^0$, Mo 3d PDOS plots shows the near-Fermi states of MoS_2 are dominated by Mo sites. The DOS in panel b and c exhibit asymmetry between spin-up and spin-down channels, due to the spin-polarized electronic structure of metallic Fe^0. (In the revised Supplementary Information)

3. The interpretation of the calculated electronic structure warrants revision for accuracy. First, the characterization of MoS2 as a "semiconductor" is not rigorously supported by the DOS in Fig. 1a, which shows a significant DOS at the Fermi level. Second, the analysis of the Mo PDOS (Supp. Fig. 2) appears inconsistent with the data presented. The text claims the d-band shifts "away" from the Fermi level, whereas the plots indicate a slight shift toward the Fermi level. Therefore, theconclusion that this shift weakens the Mo-O interaction lacks basis and may contradict established theories (such as the d-band center theory). A more precise analysis is needed to support the manuscript's mechanistic claims.

Thank you for this comment. We agree that the previous electronic-structure discussion required correction for accuracy.

To support the description of MoS_2 as a semiconductor, we compared DOS data of MoS_2 alone with the that of MoS_2|NC. The results confirm that introducing NC exhibits a higher density of states near the Fermi level, which allows electron transfer through NC to MoS_2. In addition, NC plays a critical role in modulating electronic structure of MoS_2, enhancing its catalytic properties via improved adsorption and activation of ClO_4^-, as supported by the revised adsorption energy and energy barrier data (see the response to Comments 1 and 2 for details).

In addition, regarding the Mo projected density of states (PDOS) and d-band center analysis, we conducted direct comparisons between the MoS_2|NC, MoS_2|Fe^0, and MoS_2|NC|Fe^0 systems (Fig. R9). The calculated d-band center of Mo shifts from −3.40 eV in MoS_2|NC to −3.61 eV in MoS_2|NC|Fe^0, and is −3.90 eV in MoS_2|Fe^0. However, the trend in d-band center does not correlate directly with the DFT-calculated adsorption strengths (ClO_4^- adsorption is strongest in MoS_2|NC|Fe^0), indicating that the adsorption and activation is not governed by the d-band center alone. This is consistent with prior studies showing that the d-band center is not a universal descriptor of adsorption energetics, especially when other effects contribute substantially, e.g., electronic, geometric, and electrostatic effects.[1-3] For anionic adsorption, electrostatic and charge-transfer factor can play a major role and may not be captured by d-band positions.[4,5] We therefore removed the d-band-based statement and revised the mechanistic interpretation primarily on the calculated adsorption energetics and OAT barriers.

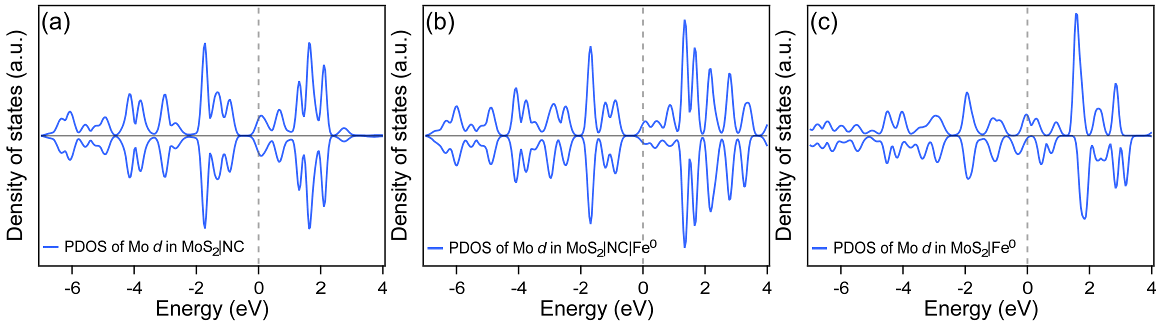

Fig. R9. Projected DOS (PDOS) of Mo in (a) MoS_2|NC, (b) MoS_2|NC|Fe^0, and (c) MoS_2|Fe^0 in Mo *d* orbits.

References:

(1) Chen, W.; Qian, G.; Wang, H.; Chen, D.; Zhou, X.; Yuan, W.; Duan, X. Rationalizing the d-Band Model from Theory to Practice in Catalyst Design. J. Am. Chem. Soc. 2025, 147 (51), 46729-46744..

(2) García-Muelas, R.; López, N. Statistical learning goes beyond the d-band model providing the thermochemistry of adsorbates on transition metals. Nat. Commun. 2019, 10 (1), 4687.

(3) Rivera Rocabado, D. S.; Nanba, Y.; Koyama, M. Density Functional Theory and Machine Learning Description and Prediction of Oxygen Atom Chemisorption on Platinum Surfaces and Nanoparticles. ACS Omega 2021, 6 (27), 17424-17432.

(4) Corrigan, K.; McCrum, I. T. The role of electron affinity in electrochemical anion adsorption on metals. The Journal of Chemical Physics 2025, 162 (14), 144703.

(5) Ringe, S. The importance of a charge transfer descriptor for screening potential CO2 reduction electrocatalysts. Nat. Commun. 2023, 14 (1), 2598.

4. The authors provide a comprehensive characterization of the defective nature of the MoS2 phase, which is convincingly linked to its catalytic activity. However, the nature of the interaction at the interface between the MoS2 crystallites and the NC support remains ambiguous (e.g., strong physisorption via van der Waals forces versus covalent bonding). Elucidating the properties of this interface is crucial for understanding the role of NC in the system.

Thank you for this comment. To clarify the nature of the interaction at the interface between the MoS_2 and NC, we provide two lines of evidence.

First, we made a comparison of spectra. As shown in Fig. R10a, C 1*s* spectra of bulk MoS_2 and MoS_2−NC are identical in peak position and shape. MoS_2−NC does not show additional features that could be assigned to Mo−C (or C−S) bonding. In the N 1*s* spectra, both bulk MoS_2 and MoS_2−NC show a peak at the same position, which is assigned to Mo 3p rather than N species (Fig. R10b). The fitting results of the N1*s* spectrum for MoS_2−NC (Fig. R10c) indicates the remaining peaks are attributable to the intrinsic N functionalities of the NC support.[1-3] Notably, the Mo−N bond, if present, would be expected in the range of 396.4-397.1 eV, as denoted with the shaded area of Fig. R10c. Whereas this range lies in the valley of the spectrum and introducing an additional peak could not lead to a satisfactory fit. Therefore, these XPS results indicate that Mo−C or Mo−N bonds are not detectable in the MoS_2−NC.

Second, as mentioned in the response to comment 2 of reviewer 1, we have conducted a control experiment with co-milled MoS_2 and NC. During its preparation, MoS_2 was milled for 3.5 h, followed by the addition of NC and the mixture was co-milled for 0.5 h. We observed a measurable reactivity (~30%) compared to the corresponding MoS_2 sample (Fig. R11). Because the co-milling time is short, and the low-temperature mechanical contact process is not expected to generate extensive covalent Mo−C or Mo−N bonds (which are more commonly formed under harsh thermal or chemical conditions[6-7]), we attribute the observed coupling effect of MoS_2 and NC primarily to interfacial contact between them, i.e., noncovalent interactions (van der Waals forces).

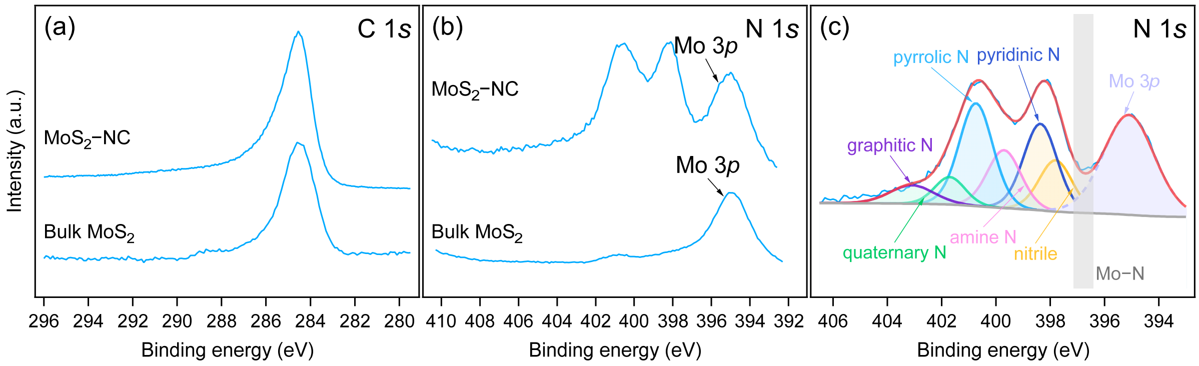

Fig. R10. XPS spectra of bulk MoS_2 and MoS_2−NC (4.2 wt.% Mo). (a) C 1*s* spectra of bulk MoS_2 and MoS_2−NC. (b) N 1*s* spectra of bulk MoS_2 and MoS_2−NC. (c) The fitting of the N 1s spectrum of MoS_2−NC. Panel c shows the several types of N species, including pyridinic N (398.4 eV), pyrrolic N (400.7), amine N (399.7 eV), nitrile (397.8 eV), quaternary N (401.7) and graphitic N (403.1 eV).[1-3] The peak in the right-hand side of panel c is assigned to Mo 3*p* based on panel b. The binding-energy range expected for Mo−N bonds (396.4-397.1 eV) is shaded in panel c,[4,5] where introducing an additional peak could not achieve satisfactory fit.

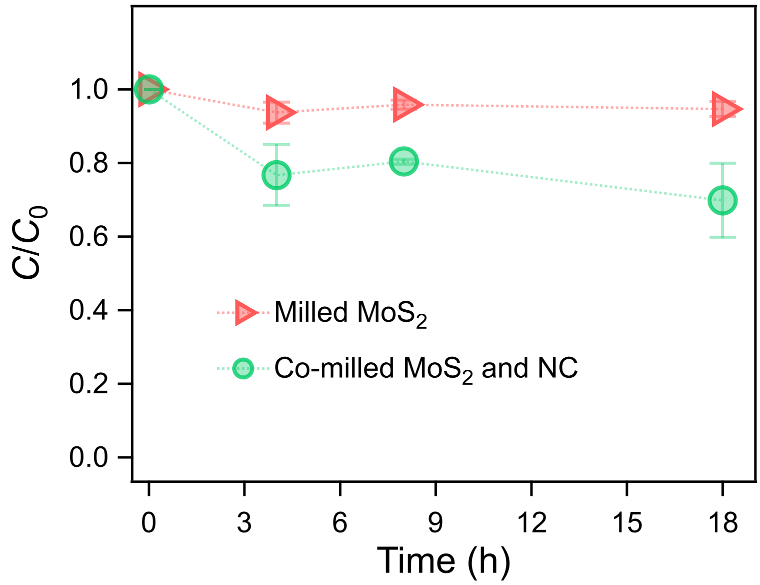

Fig. R11. ClO_4^- removal by ball-milled MoS_2 and co-milled MoS_2 and NC, with Fe^0 added during reaction. Milling followed the protocol in our previous study.[8] For milled MoS_2, milling time is 4 h; for co-milled MoS_2 and NC, MoS_2 was milled 3.5 h, after which NC was added and co-milled for 0.5 h. Reaction conditions: $[ClO_4^-]_0$ = 1.0 mM, $[Fe^0]_0$ = 1.2 g/L, $[MoS_2]$ = 0.28 g/L, [NC] = 3.72 g/L, all the concentrations refer to the final solution, and are identical to those in Fig. 3a. pH is maintained at 6.0 with 150 mM buffer, T = 25 °C.

Based on these results, we have revised the manuscript as below, and add the Fig. R1 as Supplementary Fig. 28.

Moreover, the C 1*s* spectrum of MoS_2−NC exhibits the same peak shape and position as bulk MoS_2, with no feature attributable to Mo−C (or C−S) bonds. Comparison of N 1*s* spectra of MoS_2−NC, together with the fitting results of MoS_2−NC spectrum, shows no detectable Mo−N bond (Supplementary Fig. 6). Therefore, the absence of detectable Mo−C/Mo−N bonds implies that the interfacial contact between MoS_2 and NC is dominated by noncovalent interactions (likely via van der Waals forces). (Lines 198-203 in the revised main text)

References:

(1) Kabir, S.; Artyushkova, K.; Serov, A.; Kiefer, B.; Atanassov, P. Binding energy shifts for nitrogen-containing graphene-based electrocatalysts–experiments and DFT calculations. Surf. Interface Anal. 2016, 48 (5), 293-300.

(2) Serov, A.; Artyushkova, K.; Atanassov, P. Fe−N−C oxygen reduction fuel cell catalyst derived from carbendazim: Synthesis, structure, and reactivity. Adv. Energy Mater. 2014, 4 (10), 1301735.

(3) Qin, H.; Zha, T.; Qian, K.; Sun, Y.; Guan, X.; Chen, C. Efficient full dechlorination of chlorinated ethenes on single enzyme-like Co−N_4 sites in nitrogen-doped carbons. Appl. Catal. B Environ. 2023, 328, 122459.

(4) Pan, X.; Song, X.; Lin, S.; Bi, K.; Hao, Y.; Du, Y.; Liu, J.; Fan, D.; Wang, Y.; Lei, M. A facile route to graphite-tungsten nitride and graphite-molybdenum nitride nanocomposites and their ORR performances. Ceram. Int. 2016, 42 (14), 16017-16022.

(5) Huang, Y.; Ge, J.; Hu, J.; Zhang, J.; Hao, J.; Wei, Y. Nitrogen-doped porous molybdenum carbide and phosphide hybrids on a carbon matrix as highly effective electrocatalysts for the hydrogen evolution reaction. Adv. Energy Mater. 2018, 8 (6), 1701601.

(6) Zhang, C.; Pan, Z.; Tao, Y. Synthesis and catalytic performance of Mo_2C/MoS_2 composite heterojunction catalysts. Materials (Basel) 2024, 17 (10).

(7) Zeng, L.; Chen, S.; van der Zalm, J.; Li, X.; Chen, A. Sulfur vacancy-rich N-doped MoS_2 nanoflowers for highly boosting electrocatalytic N_2 fixation to NH_3 under ambient conditions. Chem. Commun. 2019, 55 (51), 7386-7389.

(8) Chen, J.; Feng, Y.; Liu, Y.; Fan, P.; Ao, Z.; Qin, H.; Guan, X. MoS_2 modification to enhance the removal of various chlorinated hydrocarbons by zerovalent iron. Environ. Sci. Technol. 2025, 59 (22), 11330-11340.

5. Experimental results indicate that exposed Mo atoms with S vacancies are the catalytic sites. It is suggested that the authors provide comparative calculations (e.g., ClO_4^- adsorption energies and activation barriers) on the pristine MoS_2 surface compared to surfaces with varying degrees/types of S-vacancies to validate this key claim theoretically.

Thanks for your suggestion. We have performed adsorption energy calculations on different surface sites (Mo sites and S sites). In the (MoS_2|NC|Fe^0 model, the calculated adsorption energy of ClO_4^- on the S site was +1.917 eV, while Mo site with S vacancies exhibited an adsorption energy of −0.461 eV, with similar site preferences observed in MoS_2|NC and MoS_2 |Fe^0 models. These results support that the catalytically relevant adsorption/activation occurs at vacancy-exposed Mo sites rather than at S sites. While for the defect-free MoS_2 surface, the Mo sites have not been exposed, and thus is not expected to show comparable adsorption and activation for ClO_4^-.

In addition, we evaluated models (Fig. R12) with lower and higher vacancy densities around Mo sites, corresponding to (i) Mo sites associated with two S vacancies, (ii) a fully vacancy-exposed Mo layer, respectively. In both cases, structural optimization led to spontaneous Cl−O bond cleavage (i.e., barrierless dissociation during relaxation). This behavior likely arises from steric destabilization (model i) or over-stabilization of adsorbed O atoms on overly exposed Mo centers (model ii). These results are inconsistent with our experimental observations showing stable

first-order kinetics and the presence of an activation barrier**.** Thus, these models are unlikely to represent the experimentally relevant active site.

Based on this analysis, we selected a revised model with three S vacancies around the active Mo site, representing a moderate level of defect density. This model provides sufficient Mo exposure to enable adsorption and activation, while allows well-defined calculations without spontaneous dissociation. We used this 3-vacancy model as the representative defect surface for mechanistic comparisons, while treating low and high vacancy density cases as boundary checks.

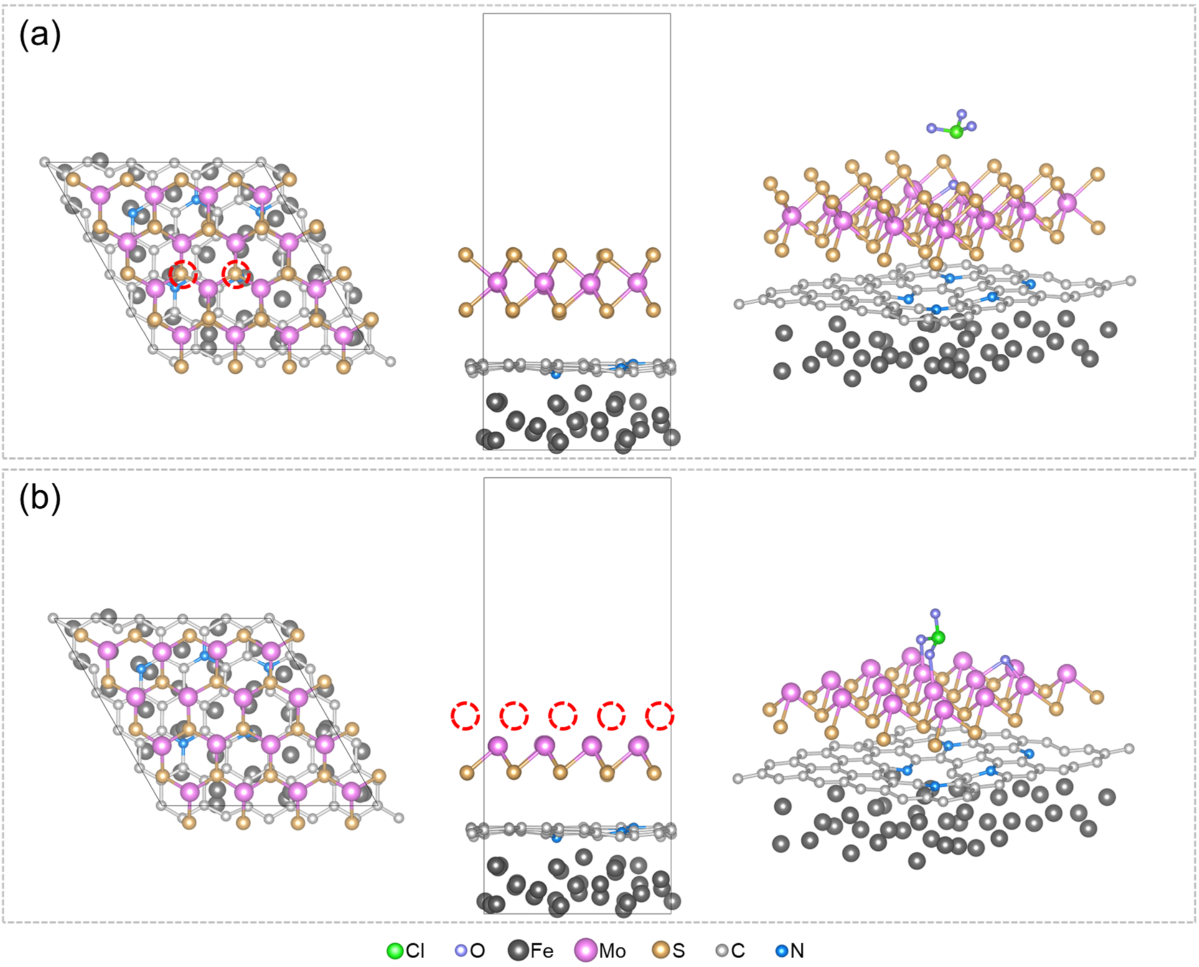

Fig. R12. MoS_2|NC|Fe^0 models with lower- and higher- defect density Mo sites: (a) Mo sites associated with two S vacancies, (b) a fully vacancy exposed Mo layer. For each panel, the left, middle, and right images show the top view, side view, and the optimized ClO_4^- adsorption configuration, respectively. S vacancies are indicated by red dashed circles, shown in the top view of (a) and the side view of (b) for clarity. The Na^+ ion used to maintain charge neutrality was omitted from the rendered structures.

6. The computational methods section lacks sufficient detail for reproducibility. Please provide more specifics on the computational setup—e.g., details on the slab model construction, the method used for transition state searching, and the specifics of the free energy calculations and any solvent models applied.

Thanks for this comment. We have added more specifics on the computational setup, including the slab model construction, (ii) the method used for transition state searching, and (iii) the specifics of the free energy calculations and (iv) solvent models applied. These additions are provided in the revised Supplementary Information:

DFT calculations.

Model construction. Structural models included MoS_2 alone (pure MoS_2 slab), MoS_2|NC, MoS_2|Fe^0, and MoS_2|NC|Fe^0 configurations (Supplementary Fig. 1). The MoS_2 slab was modeled as a 4×4 supercell of monolayer 2H-MoS_2, containing 16 Mo atoms and 32 S atoms in its pristine form. Sulfur vacancies were introduced in the top S layer by removing 3 S atoms, yielding 29 S atoms in the slab and creating undercoordinated Mo sites. The NC layer was modeled using a 5×5 graphene-based sheet (50 C atoms). Two C atoms were removed to introduce structural vacancies, and five N atoms were doped into the layer by replacing C atoms. The resulting NC model contained 43 C atoms and 5 N atoms, consistent with the XPS results showing the C/N ratio of approximately 8:1. The Fe^0 layer was modeled as a 3×3 Fe(110) slab consisting of 36 Fe atoms. These model sizes (4×4 for MoS_2, 5×5 for NC, and 3×3 for Fe) were selected to enable lateral alignment across layers with minimal lattice mismatch (<10%) after strain optimization. The MoS_2|NC|Fe^0 structure was constructed by vertically stacking the layers in the order of MoS_2, NC, and Fe slabs, with a vacuum region of 14 Å above the MoS_2 surface along the z-axis to avoid periodic interlayer interactions. To represent the aqueous reaction environment, we used the VASPsol implicit solvation model in all DFT calculations, therefore, the region above the surface represents a continuum solvent environment rather than a true vacuum. The MoS_2|NC and MoS_2|Fe^0 models were constructed similarly, with MoS_2 directly interfaced with the NC or Fe layer, respectively.

Computational settings. Spin-polarized density functional theory (DFT) calculations were performed using the Vienna Ab-initio Simulation Package (VASP) with projector augmented wave (PAW) pseudopotentials. The exchange-correlation effects were treated using the generalized gradient approximation (GGA) with the Perdew-Burke-Ernzerhof (PBE) functional. A plane-wave energy cutoff of 450 eV was applied. The sampling of the Brillioun zone was performed using a Monkhorst-Pack k-points grid of 2 × 2 × 1, corresponding to a k-point separation length of 0.05 $Å^{-1}$. Structural optimizations were carried out using the quasi-Newton l-BFGS method until the energy and force converged to less than 1.0×10^{-5} eV and 0.01 eV $Å^{-1}$, respectively. Long-range dispersion interactions were included using Grimme's DFT-D3 correction (VASP tag IVDW = 12) to account for noncovalent interactions at the MoS_2−NC, MoS_2−Fe^0, and NC−Fe^0 interfaces. All calculations were spin-polarized (ISPIN = 2) with MAGMOM initialized for Fe-containing slabs. To consider the solvation of the system, the value of EK_B for VASPsol was 78 to represent the water condition.

Charge analysis and electronic structure. Bader charge analysis was used to quantify charge transfer between layers. To enable comparison across models, the net transferred electrons (Δq, in e^-) were further normalized by the interfacial area of the simulation cell (A) in the a-b plane, calculated as $A = a\cdot b\cdot \sin\gamma$ (for the present hexagonal cell with $a = b = 12.41$ Å and $\gamma = 120°$). The area-normalized charge transfer was reported as $\Delta q/A$ (e^-/nm^2). Charge density difference plots were calculated using two-fragment decompositions in the same simulation cell and atomic geometry as the corresponding combined system (single-point charge densities). This approach highlights electron redistribution within the donor−acceptor structure. Spin density was obtained from CHGCAR and visualized to confirm the spin distribution. The converged magnetic moments were obtained from OUTCAR. Density of states (DOS) and projected DOS (PDOS) were calculated to evaluate the electronic conductivity and contributions from specific elements near the Fermi level.

All visualizations were generated using VESTA. The files of CDD and DOS were generated from VASPKIT.

Processing of free energies. Adsorption energies (E_{ads}) were calculated as:

$E_{ads} = E_{surface + adsorbate} - E_{surface} - E_{adsorbate}$

where $E_{surface + adsorbate}$ and $E_{surface}$ are the total energies of the relaxed adsorbate-surface complex and the clean surface, respectively, and $E_{adsorbate}$ is the energy of the isolated adsorbate computed in the same settings.

To simulate aqueous-phase conditions, all adsorption and reaction calculations were performed with the VASP implicit solvation model (VASPsol). For anionic adsorbates (e.g., ClO_4^-), a Na^+ counterion was introduced in the cell to maintain charge neutrality. The Na^+ was initially placed in the solvent-accessible region away from the adsorption site and allowed to relax during geometry optimization. In optimized structures, Na^+ did not form direct coordination bonds to the active site and remained solvated (Supplementary Fig. 4). In Fig. 1, the Na^+ was not included for clarity, because it resides far from the surface and may misleadingly suggest the direct involvement in the reaction mechanism.

Reaction energetics were reported using a free-energy approximation. Gibbs free energies at 298 K (U = 0 V) were calculated as $G(T) = E_{DFT} + E_{ZPE} + \Delta H(T) - TS(T)$. The ZPE-corrected energy was used as the 0 K enthalpy reference. The ZPE-corrected energy was used as the 0 K enthalpy reference. E_{ZPE}, ΔH, and S values were obtained from vibrational frequency calculations using VASPKIT. The energy barriers for Cl−O bond cleavage from adsorbed ClO_4^- ($*ClO_4 \rightarrow *ClO_3 + *O$) were computed using the climbing image nudged elastic band (CI-NEB) method. Subsequent steps including $*O \rightarrow *OH$ hydrogenation and OH^- desorption, were evaluated using free energy changes (ΔG) with ZPE and entropy contributions at 298 K. For the hydrogenation of surface O atom (e.g., $*O + H^+ + e^- + \rightarrow *OH$), the reaction energy change was calculated by $\Delta E = E_{OH*} - E_{O*} - (E_{H^+} + E_{e^-})$, where E_{OH*} is the total energy of the surface with OH, and, according to the reaction of $H^+ + e^- \rightarrow 1/2H_2{}^-$, $E_{H^+} + E_{e^-}$ is estimated from the half the energy of H_2 gas ($1/2E_{H2} - kT\ln[H+]$). Accordingly, pH effects were included by applying a thermodynamic correction of 0.0592×pH at 298 K per transferred ($H^+ + e^-$). Unless otherwise noted, pH was set to 6.0 to match experimental conditions. (Lines 65-136 in the revised Supplementary Information)

7. The evidence for the second role of the NC support (lines 336-357) is not clear. A contradiction exists between the experimental XPS results (showing no Mo-N bonds) and the proposed mechanism from cited literature (Refs 52, 53), which relies on Mo-N bond formation to create S-vacancies. It is suggested that the authors provide more direct evidence, such as comparative EPR data for the MoS_2-AC and MoS_2-AC+N control materials, to substantiate the claim that N-doping increases S-vacancy concentration.

We agree with you that this point required clarification. First, we confirm that the XPS results do not show evidence of Mo−N bond in MoS_2−NC, as we discussed in the response to your comment 4. However, this does not exclude a transient role of N species during synthesis. Under high-temperature pyrolysis, Mo−N coordination may form as an intermediate (as suggested in Refs. 52, 53), facilitating local lattice distortion and S removal. The subsequent N loss can eliminate Mo−N bonding in the final material while leaving S vacancies.

To further support this hypothesis, we performed EPR measurements on MoS_2−AC and MoS_2−NC+N. The MoS_2−NC+N sample exhibits significantly stronger EPR signals at g ≈ 2.003, which attributed to S-vacancies. This result provides direct

experimental evidence that N-doping during synthesis enhances the concentration of S-vacancies, despite the absence of detectable Mo−N bonds in the final material.

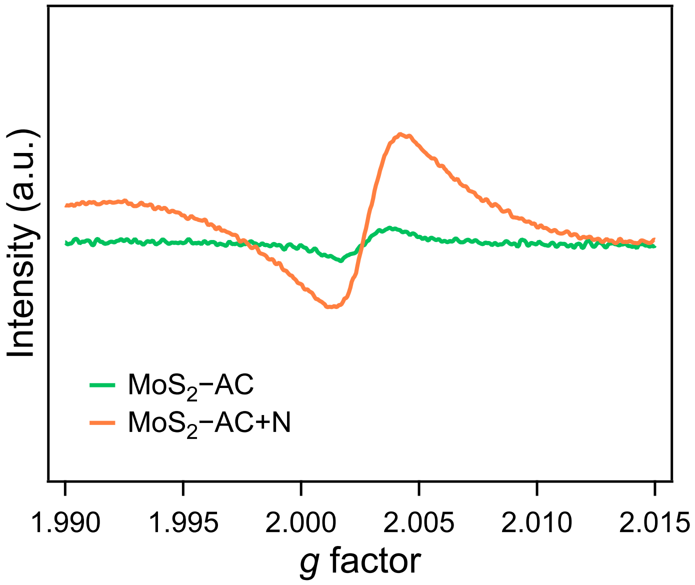

Fig. R13. The EPR spectra for MoS_2−AC and MoS_2−AC+N.

These new EPR results are now included in the revised Supplementary Information (use the Fig. R13 as Supplementary Fig. 31) and are discussed in the revised main text.

> To provide direct experimental support, we performed EPR analysis, which revealed a stronger S vacancies signal for MoS_2−AC+N compared to MoS_2−AC (Supplementary Fig. 31), confirming that N doping increases S-vacancy concentrations. (Lines 397-400 in the revised main text)

8. The technoeconomic analysis is useful but appears oversimplified. Considering only the metal cost does not fully capture the OPEX and CAPEX differences between Fe^0-based and Pd/H_2 systems. Fe^0 corrosion, iron sludge generation, solid–liquid separation, catalyst replacement frequency, and carbon-support cost can significantly influence the overall economics and should be discussed for a more realistic comparison. A performance-normalized cost comparison (e.g., equal treatment capacity or equivalent benefit-cost ratio) would further enhance the robustness of the analysis. Clarifying the assumed catalyst lifetimes and including basic sensitivity analyses would also help substantiate the claim of cost competitiveness.

Thank you for this constructive comment. We agree that the original TEA was too simplified. In the revised manuscript, we expanded the TEA to provide a performance-normalized comparison between the MoS_2−NC/Fe^0 and the Pd/H_2-based system, incorporating Fe^0 consumption, iron sludge generation and solids handling, solid-liquid separation, catalyst replacement, and other factors. We also added a CAPEX discussion. The detailed revised TEA is provided in Supplementary Note 1. The TEA section in the manuscript was revised accordingly.

In the Supplementary Note 1:

Cost Assumptions for Catalysts and Reagents

Based on the experimental results using coarser Fe^0 powder, we conducted TEA based on realistic commercial prices and industrial prices for both MoS_2−NC/Fe^0 and Pd/H_2-based system.

A preliminary consumables-based cost comparison was performed on a 1 L treatment basis with an initial ClO_4^- concentration of 1 mM. For the MoS_2−NC/Fe^0 system, 4 g/L of MoS_2−NC (4.2 wt.% Mo) and 12 g/L of coarser Fe^0 were applied. Under these conditions, 12 g/L Fe^0 removed 80% of 1 mM ClO_4^- in 18 h; thus, the Fe^0 cost was normalized by 1/0.8 to enable comparison on an equivalent (per 1 mM removed) basis. Using industrial bulk prices for coarser Fe^0 of $4.4-$10.22 per kg (from iron-filing.com, chemicalstore.com, and ScienceKitStore.com), the normalized Fe^0 cost was $0.066-$0.153 per L. The Mo precursor for MoS_2−NC synthesis, ammonium tetrathiomolybdate, was priced at $18-$33 per kg (from scrapmonster.com), contributing $0.0082−$0.0150 per L based on the Mo loading (4.2 wt.% Mo). The NC fraction in MoS_2−NC was estimated as ~93 wt.% (3.72 g/L). The base carbon price was taken as $1.77-$2.54 per kg using industrial activated carbon and carbon black references (from businessanalytiq.com, indexbox.io, businessanalytiq.com, and imarcgroup.com), and the NC price was estimated by applying a 1.5-6.5× multiplier to account for nitrogen doping and the associated yield penalty, processing energy, etc., following published cost-estimation approaches for functionalized carbons, giving an NC price of $2.66-$16.51 per kg. This corresponds to an NC cost contribution of $0.0099-$0.061 per L. Therefore, the total cost of the MoS_2−NC/Fe^0 system is $0.084-$0.229 per L, i.e., 21-87× lower than the Pd/H_2 comparator under the same removal basis.

For the Pd/H_2-based system, we estimated the cost of the [$(NH_2)_2$*bpy*]MoO_x−Pd/C system, where 0.2 g/L catalyst (5 wt.% Pd, 5 wt.% Mo) achieved complete removal of 1 mM ClO_4^-. Using bulk-pack supplier catalog prices for 5 wt.% Pd/C, the catalyst cost was estimated as $11,450-$17,840 per kg (from strem.com, fuelcellearth.com, srlchem.com), corresponding to $2.30-$3.57 per L at 0.2 g/L. The Mo precursor $Na_2MoO_4 \cdot 2H_2O$ was priced at $13-$15 per kg (from made-in-china.com and contributing negligibly ($0.00033-$0.00038 per L). The ligand $(NH_2)_2$*bpy* (assumed 1:1 molar with Mo) was priced at $125.8-$189.4 per g (from chemicalbook.com, biosynth.com, and tcichemicals.com), contributing $2.44-$3.68 per L. Therefore, the consumables cost for the Pd/H_2 system was $4.74-$7.25 per L without operational costs. In addition, Pd/H_2-based operation typically requires acidification to pH 3 and subsequent neutralization (estimated ~$0.1 per L depending on alkalinity) and a continuous H_2 supply (~$0.003 per L), resulting in a total estimated cost of $4.84-$7.35 per L.

All the prices used to estimate the cost were obtained from publicly available online listings for bulk-pack reagents from major suppliers and commercial distributors. Therefore, these values should be interpreted as order-of-magnitude estimates rather than absolute costs. In addition, the calculations above represent a first-run estimate, i.e., the full material inputs were charged to a single treatment run to enable a consistent comparison of the dominant cost contributors between the two systems. In continuous operation, catalyst reuse could reduce the effective cost per treated volume. We further calculated the operating expenditure below.

Operating expenditure (OPEX) comparison

For the MoS_2−NC/Fe^0 system, the OPEX includes (i) Fe^0 consumption (supplemental Fe^0 dosing), (ii) solid/sludge handling and disposal, (iii) MoS_2−NC supplementation, and (iv) utilities/maintenance. For 1 mM ClO_4^- removed per m^3 (1 mol/m^3), the stoichiometric Fe^0 demand is 0.223 kg/m^3. With an electron efficiency of 20%-40%, the net Fe^0 demand is 0.56-1.12 kg/m^3, corresponding to $2.5-$11.5/m^3 using the industrial Fe^0 price mentioned above. The consumed Fe is converted to $Fe(OH)_2$/$Fe(OH)_3$ solids, which requires separation, dewatering, and disposal. Using reported dewatered sludge disposal cost benchmarks (e.g., 300-800/dry ton), and kg-scale dry solids generation per m^3 (~1-2 kg dry solids per m^3, depending on $Fe(OH)_x$ form), the sludge handling/disposal cost is estimated as $0.3-$1.9/m^3. For MoS_2−NC supplementation, the initial

catalyst input was estimated to \$18-\$76/m^3 as discussed above. The deactivation fraction per run was calculated from the half-life and run time. From the Fig. 3h, the half-life was estimated to be 3.3 days, with a run time of 18 h (Supplementary Fig. 32) and 1% catalyst lost for each run. Because each run removes only 80% of ClO_4^-, the catalyst cost per m^3 treated was normalized to report cost per 1 mM removed. The cost of the catalyst supplementation would be \$3.50-\$14.80/m^3. Finally, for utilities and maintenance, wastewater treatment energy use commonly is 0.30-1.89 kWh/m^3, while routine maintenance is site-specific but ignorable relative to material supplementation and solids handling. Given the U.S. industrial electricity price benchmark (~\$0.081/kWh), the electricity cost is \$0.02-\$0.15/m^3. Summing these terms, the estimated OPEX for MoS_2−NC/Fe^0 is ~\$6.27-\$28.25 per m^3 (for 1 mM ClO_4^- removed), dominated by Fe^0 consumption and sludge management.

For the Pd/H_2-based [$(NH_2)_2$*bpy*]MoO_x−Pd/C system, the OPEX should include (i) catalyst supplementation, (ii) H_2 supply, (iii) acidification to pH 3 and subsequent neutralization, and (iv) utilities/maintenance. On the same removal basis used above (1 mM ClO_4^- removed per m^3), the conversion of ClO_4^- to Cl^- is an 8-electron reduction, implying a stoichiometric H_2 demand of 4 mol H_2 per mol ClO_4^- (~0.008 kg H_2/m^3). Assuming 1-2× excess to cover inefficiencies, this corresponds to 0.008-0.016 kg H_2/m3 and account for \$0.05-\$0.29 m^{-3} (\$6.2-18/kg H_2 from NREL Annual Technology Baseline (ATB) 2024). For pH adjustment, the chemical cost is estimated as \$0.03-\$0.11 m^{-3}. Electricity is the same as above, which is \$0.02-\$0.15/m^3. In addition, catalyst supplementation dominates the OPEX of Pd/H_2 system. The first-run estimate of the catalyst is \$4.74-\$7.25 per L if treated as single-pass dosing (i.e., no reuse). The half-life was calculated to be 4.8 days according to the reported rate decay. Assuming 1 h hydraulic retention time (HRT, estimated with batch cycle time) and 1% of the catalyst lost for each run, the cost of catalyst supplementation would be \$75.9-\$116.2/m^3. The total cost would be estimated as \$76.0-\$116.8/m^3.

Capital expenditure (CAPEX) comparison

For the MoS_2−NC/Fe^0 system, capital costs are primarily driven by (i) reactor volume and (ii) solid separation and dewatering systems. Under the long HRT (18 h) used for the reduction process, the reactor requires large tankage, with mixers, pumps, and piping designed for slurry. Because Fe^0 is sacrificial and converts to $Fe(OH)_2$/$Fe(OH)_3$ solids during reaction, the system typically requires solid-liquid separation followed by mechanical dewatering (e.g., clarifier or thickener coupled with belt press, centrifuge, or filter press), along with sludge handling and loadout. Sludge management (including dewatering, handling, and transport interfaces) is widely identified as a major design and costing element in municipal and industrial wastewater facilities, and thus is expected to be a dominant CAPEX contributor for Fe^0-based treatment. Overall, this system has moderate mechanical complexity (no combustible gas system), but high installed cost sensitivity to reactor volume and sludge management.

For the [$(NH_2)_2$*bpy*]MoO_x−Pd/C system, A gas-liquid reactor is generally required, which can be packed bed, trickle bed, or stirred reactor with gas sparging. Because H_2 delivery to the liquid phase can be rate-limiting, it requires facilities to enhance mass transfer, like fine-bubble gas diffusion and mild pressurization, which increases mechanical and controls complexity relative to an all-liquid system. The core CAPEX item is the hydrogen supply and safety infrastructure, including H_2 storage (gas container or onsite supply), regulation and piping, and safety systems (hydrogen detection, alarms/interlocks, and ventilation) to prevent accumulation of flammable mixtures. Meanwhile, the acidification and subsequent neutralization generally requires chemical storage tanks, metering pumps, mixers. Overall CAPEX implication: Lower tank volume (shorter HRT) can reduce reactor civil costs, but the hydrogen supply system with safety system and pH adjustment tends to push CAPEX to moderate-high, and it is more permitting-/hazard-driven than the Fe^0 system. Consequently, although shorter HRT can reduce reactor costs, the hydrogen supply and safety systems and pH-adjustment infrastructure lead to overall moderate-high CAPEX.

Overall, the Fe^0-based system tends to have CAPEX dominated by reactor volume and solids management, whereas the Pd/H_2 is dominated by hydrogen supply and safety systems and pH adjust infrastructure. Practically, this implies that Fe^0 systems often have lower hazard-driven

CAPEX but higher solids-management CAPEX, while Pd/H_2 systems are the opposite due to hydrogen handling and safety compliance.

CAPEX contributor table for the two systems

Category	MoS_2−NC/Fe^0 system	Pd/H_2 system	Notes
Reactor	Large volume (long HRT)	Smaller volume (short HRT)	
Gas supply system	None	H2 storage, regulation and piping	H_2 infrastructure is a distinct capital item (gas supply, controls, venting).
Hydrogen safety system	None	Detectors, alarms/interlocks, ventilation	H_2 facilities typically require detector-triggered shutdown/alarms and ventilation facilities to keep H_2 below flammability thresholds.
Solid separation	Required	Required for catalyst recycle	Fe^0 generates solids continuously, so solid-liquid separation is necessary. Pd/C may need filtration if fines need to be retained for recycling.
Dewatering equipment	Required	None	Mechanical dewatering is often a major cost contributor.
Sludge handling facilities	Required	None	$Fe(OH)_x$ solids require handling space and equipment.
Acid/base storage and dosing system	Optional	Required for acidification and neutralization	Pd/H_2 at pH 3 needs acid and base feed systems (tanks, mixers, metering pumps)
Materials of construction	Corrosion considerations	Acid and H_2 compatibility	Fe slurries require wear-resistant design; Pd/H_2 requires acid-resistant wetted parts and H_2-safe components.
Instrumentation and controls (I&C)	Standard water controls	Enhanced safety for I&C	Pd/H_2 typically requires additional safety devices (detectors, control system, hardware/software for H_2 ventilation status)

(Lines 309-414 in the revised Supplementary Information)

We also updated the TEA section in the revised main text accordingly:

Technoeconomic analysis for the MoS_2−NC/Fe^0 system

A preliminary technoeconomic analysis (TEA) was conducted to compare the MoS_2−NC/Fe^0 system with representative Pd/H_2-based catalytic systems for ClO_4^- reduction. They share a similar conceptual process design (fixed-bed or slurry contactor followed by solids separation), making the Pd/H_2-based systems the most representative comparator among abiotic ClO_4^- reduction approaches. In Pd/H_2-based systems, the typical catalyst loading is 0.2 g/L containing 5 wt.% Pd and 5 wt.% Re or Mo, operating under 1 atm H_2 at pH 3. For the MoS_2−NC/Fe^0 system, we evaluated a coarser Fe^0 powder (20 mesh) to reflect practical cost considerations. At 4.0 g/L MoS_2−NC (4.2 wt.% Mo) with 12 g/L Fe^0, the system achieved ~80% ClO_4^- removal in 18 h (Supplementary Fig. 33), and all costs were normalized to an equivalent basis (per 1 mM removed), and the detailed TEA is provided in the Supplementary Note 1.

On a 1 L treatment basis (1 mM initial ClO_4^-), a consumables-based evaluation using bulk commercial pricing yields a total materials cost of \$0.084-\$0.229 per L for MoS_2−NC/Fe^0, versus \$4.84-\$7.35 per L for the typical Pd/H_2-based comparator when including typical acidification/neutralization and H_2 supply. The cost of MoS_2−NC/Fe^0 is dominated by Fe^0 and the N-doped carbon fraction, while the Pd/H_2-based system cost is dominated by noble-metal catalyst and required ligand. Since these values represent a first-run estimate (i.e., full material inputs charged to a single treatment run), we further extended the analysis to operating expenditure (OPEX) for continuous operation on a 1 mM removed per m^3 basis. Under the assumptions described in the Supplementary Information, MoS_2−NC/Fe^0 is estimated at \$6.27-\$28.25 per m^3, dominated by net Fe^0 consumption (Fe^0 supplementation) and slurry management, whereas Pd/H_2 is estimated at \$76.0-\$116.8 per m^3, dominated by catalyst supplementation, with H_2 and pH-control costs. It should be noted that these estimates should be interpreted as order-of-magnitude benchmarks, instead of absolute values, because pricing and operating conditions are site-specific and the calculations adopt conservative assumptions on losses and deactivation, but the results consistently indicate a cost advantage for MoS_2−NC/Fe^0 under practical pricing.

From a capital expenditure (CAPEX) perspective, the MoS_2−NC/Fe^0 system is expected to be driven mainly by reactor volume (long hydraulic retention time (HRT)) and solids separation/dewatering infrastructure. In comparison, the Pd/H_2-based system has the advantage of shorter HRT but requires H_2 supply and safety systems to prevent the accumulation of flammable H_2 mixtures, which increase mechanical and permitting complexity. In addition, the acid/base storage and dosing infrastructure contributes substantially to the CAPEX. Overall, the MoS_2−NC/Fe^0 system offers advantages in safety and operational simplicity (near-neutral pH, no combustible gas), supporting its potential for cost-effective and scalable ClO_4^- treatment. In future studies, the Fe^0 could be replaced by an electrochemical cathode as the electron donor, which could further increase the cost-effectiveness, by reducing Fe^0 consumption, solid generation, and the associated slurry management. (Lines 421-457 in the revised main text)

Reviewer #3

General Comments:

The manuscript "Donor−acceptor catalysis with defective MoS2 and Fe0 breaks barriers to perchlorate reduction under mild conditions" is a joint computational-experimental study, and I was asked for consideration of the computational part.

My problem with the computational part of the manuscript is that, in its current form, it is irreproducible, the computational model is not well described, the methods are questionable, and the conclusions are not sufficiently supported by the obtained data.

Thank you for your careful evaluation of the computational part. We agree that the original version lacked sufficient detail for reproducibility and that some computational interpretations were not adequately supported. In the revised manuscript and SI, we re-conducted the whole DFT part using revised, well-defined interface models and updated computational settings. We also re-wrote the Method section (in SI) to clearly document the model construction, key settings, and the protocol for Gibbs free-energy calculations. In addition, we revised the DFT interpretation and added supporting calculations and figures so that the mechanistic conclusions are consistent well with the updated computational results. We believe these changes strengthen the computational part and its support for the mechanistic conclusions. Below are our detailed responses.

Specific Comments:

1. Computational model: In "Computational methodology" section (page 20), it is just stated "The computational models were constructed based on characterization results, with sulfur vacancies introduced into the MoS2 crystal structure to simulate defect sites" and link to Supplementary Figure 1, however, there is only top and side structural model and it is not clear, a) what is the unit cell, b) how MoS2/CN interface was constructed (incommensurate supercell?), c) why just six atoms is included in Fe cluster, etc.

Thank you for pointing out the need for more detail about model construction. In the revised manuscript, we have updated the computational model to better reflect the experimental system.

a) Unit cells. The computational model is based on a 4×4 2H-MoS_2 supercell (containing 16 Mo and 32 S atoms in the pristine configuration).[1,2] The sulfur vacancies are introduced to the MoS_2 surface by removing 3 S atoms, the MoS_2 slab contains 29 S atoms (relative to 32 S in pristine 4×4 MoS_2), i.e., three S vacancies in this supercell, yielding undercoordinated Mo sites. To model the NC layer, we built a 5×5 graphene-like carbon layer. Two C atoms were removed to reflect the vacancies of the NC model and 5 N atoms were doped into the layer.[3] In the current model, the NC layer contains 43 C atoms and 5 N atoms, consistent with the XPS results showing the C/N ratio is about 8:1. The Fe^0 layer was modeled as a Fe(110) slab consisting of 36 Fe atoms.[4] In all the models, a vacuum region of >14 Å above the surface along the z-axis to avoid periodic interlayer interactions (Fig.

R14). These model sizes (4×4 for MoS_2, 5×5 for NC, and 3×3 for Fe) were selected to enable lateral alignment across layers with minimal lattice mismatch (<10%) after strain optimization.

b) Construction of the MoS_2/NC interface. First, the 5×5 NC layer was designed to match the MoS_2 basal plane with minimal lattice mismatch after strain optimization. All components were relaxed together to determine interfacial registry and equilibrium distances. The in-plane lattice vectors of all layers are constrained to match the MoS_2 4×4 cell to avoid artificial strain and incommensurate supercell artifacts.

c) Fe slab. To more accurately reflect the role of NC and represent Fe^0 as a bulk electron donor, we have replaced the Fe6 cluster used in preliminary models with a periodic Fe^0 slab, exposing the Fe(110) surface, which is the most stable and widely used facet of body-centered cubic (bcc) iron in DFT studies. The slab in the current model consists of 36 Fe atoms, spanning multiple atomic layers. This Fe(110) surface allows consistent modeling of electron transfer to the NC and MoS_2 layers.

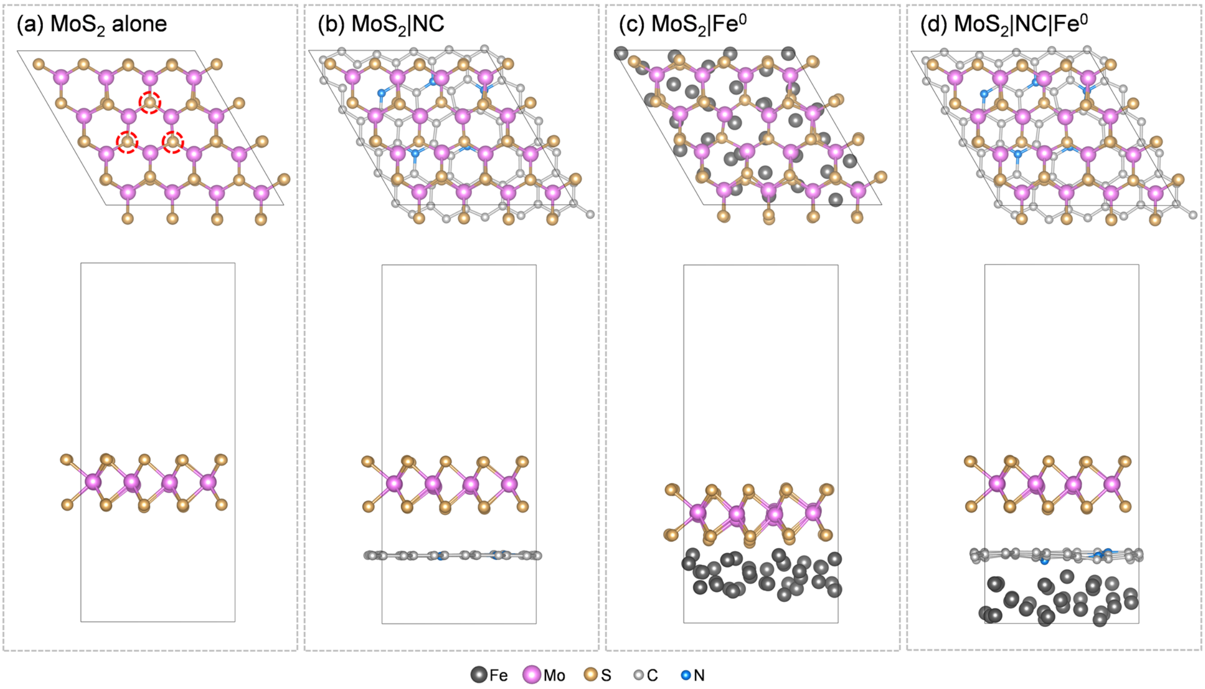

Fig. R14. Structural models of (a) MoS_2 alone, (b) MoS_2|NC, (c) MoS_2|Fe^0, and (d) MoS_2|NC|Fe^0. Each panel shows both the top view (upper) and side view (lower) of the optimized structures. Red dashed circles in (a) represent the positions of sulfur vacancies, which are identically introduced in all four models but are not denoted in panels (b-d) for clarity.

Accordingly, we add the model structure (use the Fig. R14 as Supplementary Fig. 1) and revised the Model construction method:

> *Model construction*. Structural models included MoS_2 alone (pure MoS_2 slab), MoS_2|NC, MoS_2|Fe^0, and MoS_2|NC|Fe^0 configurations (Supplementary Fig. 1). The MoS_2 slab was modeled as a 4×4 supercell of monolayer 2H-MoS_2, containing 16 Mo atoms and 32 S atoms in its pristine form. Sulfur

vacancies were introduced in the top S layer by removing 3 S atoms, yielding 29 S atoms in the slab and creating undercoordinated Mo sites. The NC layer was modeled using a 5×5 graphene-based sheet (50 C atoms). Two C atoms were removed to introduce structural vacancies, and five N atoms were doped into the layer by replacing C atoms.[3] The resulting NC model contained 43 C atoms and 5 N atoms, consistent with the XPS results showing the C/N ratio of approximately 8:1. The Fe^0 layer was modeled as a 3×3 Fe(110) slab consisting of 36 Fe atoms. These model sizes (4×4 for MoS_2, 5×5 for NC, and 3×3 for Fe) were selected to enable lateral alignment across layers with minimal lattice mismatch (<10%) after strain optimization. The $MoS_2|NC|Fe^0$ structure was constructed by vertically stacking the layers in the order of MoS_2, NC, and Fe slabs, with a vacuum region of 14 Å above the MoS_2 surface along the z-axis to avoid periodic interlayer interactions. To represent the aqueous reaction environment, we used the VASPsol implicit solvation model in all DFT calculations, therefore, the region above the surface represents a continuum solvent environment rather than a true vacuum. The $MoS_2|NC$ and $MoS_2|Fe^0$ models were constructed similarly, with MoS_2 directly interfaced with the NC or Fe layer, respectively. (Lines 66-83 in the revised Supplementary Information)

References:

(1) Tsai, C.; Li, H.; Park, S.; Park, J.; Han, H. S.; Nørskov, J. K.; Zheng, X.; Abild-Pedersen, F. Electrochemical generation of sulfur vacancies in the basal plane of MoS_2 for hydrogen evolution. Nat. Commun. 2017, 8 (1), 15113.

(2) Li, H.; Tsai, C.; Koh, A. L.; Cai, L.; Contryman, A. W.; Fragapane, A. H.; Zhao, J.; Han, H. S.; Manoharan, H. C.; Abild-Pedersen, F.; Nørskov, J. K.; Zheng, X. Activating and optimizing MoS_2 basal planes for hydrogen evolution through the formation of strained sulphur vacancies. Nat. Mater. 2016, 15 (1), 48-53.

(3) Błoński, P.; Tuček, J.; Sofer, Z.; Mazánek, V.; Petr, M.; Pumera, M.; Otyepka, M.; Zbořil, R. Doping with graphitic nitrogen triggers ferromagnetism in graphene. J. Am. Chem. Soc. 2017, 139 (8), 3171-3180.

(4) Xu, L.; Kirvassilis, D.; Bai, Y.; Mavrikakis, M. Atomic and molecular adsorption on Fe(110). Surf. Sci. 2018, 667, 54-65.

2. In addition, it is not clear if there is a right ground state of the system. On the one hand, it is stated (page 20) that "Spin-polarized density functional theory" was used; on the other hand, no information on the resulting spin distribution (spin density) is provided. The most complicated part of the model, the iron cluster, is not an easy task, and, typically, a high-spin solution is preferred. In Phys. Chem. Chem. Phys., 2020, 22, 23258, e.g., Fe4 cluster used for molecular adsorption embodied 13-tet configuration (i.e., multiplicity 13, magnetic moment 12 muB, ESI there) or Fe7 cluster is of multiplicity 23.

Thank you. In both the original and updated calculations, spin-polarized DFT was employed for all geometry optimizations and electronic structure calculations. The spin magnetic moments were allowed to relax self-consistently during geometry optimization.

In the original manuscript, the Fe^0 particle was represented by a small Fe6 cluster, and spin polarization was enabled to allow convergence to a physically reasonable high-spin state. In the updated model, the Fe6 cluster has been replaced by a periodic Fe(110) slab consisting of 36 Fe atoms (a 3×3 supercell with multiple layers, see updated model in Fig. R14 in the response to the Comment 1 and revised SI). By using the slab, we avoid the cluster-related spin-state ambiguities. All revised DFT calculations were performed with spin polarization enabled (ISPIN = 2 in VASP). Spin-polarized calculations were initialized with a magnetic moment of 4.0 μB per Fe atom. After geometry optimization, the slab retained a ferromagnetic solution with an average local Fe moment of 2.57 μB per Fe (sum of site-projected moments

divided by 36 Fe atoms). This value is slightly higher than bulk bcc Fe (~2.2 μB), which is expected due to surface moment enhancement in thin slabs.

We further provide the spin-density distribution of the optimized MoS_2|NC|Fe^0 model (Fig. R15). The spin density is primarily localized on the Fe atoms, confirming that the optimized structure remains spin-polarized. The negligible spin density on the NC and MoS_2 layers indicates that the magnetic properties are dominated by the Fe slab, as expected.

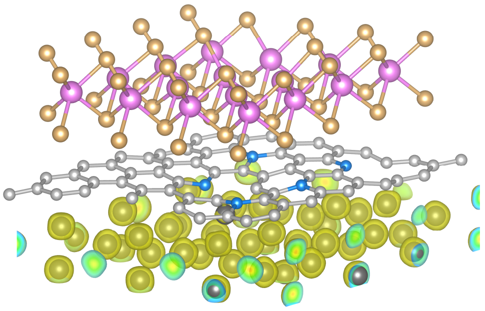

Fig. R15. Spin density distribution of the relaxed MoS_2|NC|Fe^0 model.

Therefore, our updated slab model reflects a realistic ferromagnetic Fe^0 model, and all calculations were carried out with spin polarization to describe the magnetic electronic structure of the system.

3. Moreover, it is stated that "ClO4− preferentially adsorbs at undercoordinated Mo sites over S sites" (page 5). But negatively charged ions in periodic boundary conditions are a very challenging task (lattice sums over charged cells diverge), and there is no mention of how exactly this was modelled. By restricting the number of electrons in the input file? By some other counterion? Via some special corrections?

Thank you for raising this important point. We agree that modeling charged species in periodic boundary conditions requires proper charge compensation to avoid unphysical divergence of electrostatic energy.

In the revised manuscript, all calculations involving ClO_4^- species were re-performed using charge-neutral models, where a Na^+ ion was introduced into the cell whenever ClO_4^- was present.[1-3] It should also be noted that we used the VASPsol implicit solvation model in all DFT calculations to simulate the water environment, therefore, the Na^+ ion exist in a continuum solvent environment rather than a true vacuum. This ensures overall charge neutrality of the periodic system and avoids the divergence issues associated with charged supercells.

The Na^+ ion was initially placed in the solvent-accessible region, away from the adsorption site, and allowed to relax during geometry optimization. In all optimized structures, Na^+ did not participate directly in Mo−O bonding or alter the adsorption geometry at the active site (Fig. R16). This treatment is consistent with commonly

adopted approaches for modeling anionic adsorbates in periodic systems. With the revised model, the conclusions on ClO_4^- adsorption preference remain unchanged. For example, in the MoS_2|NC|Fe^0 model, the adsorption energy of ClO_4^- at Mo sites remains more favorable (−0.461 eV) than at S sites (+1.917 eV).

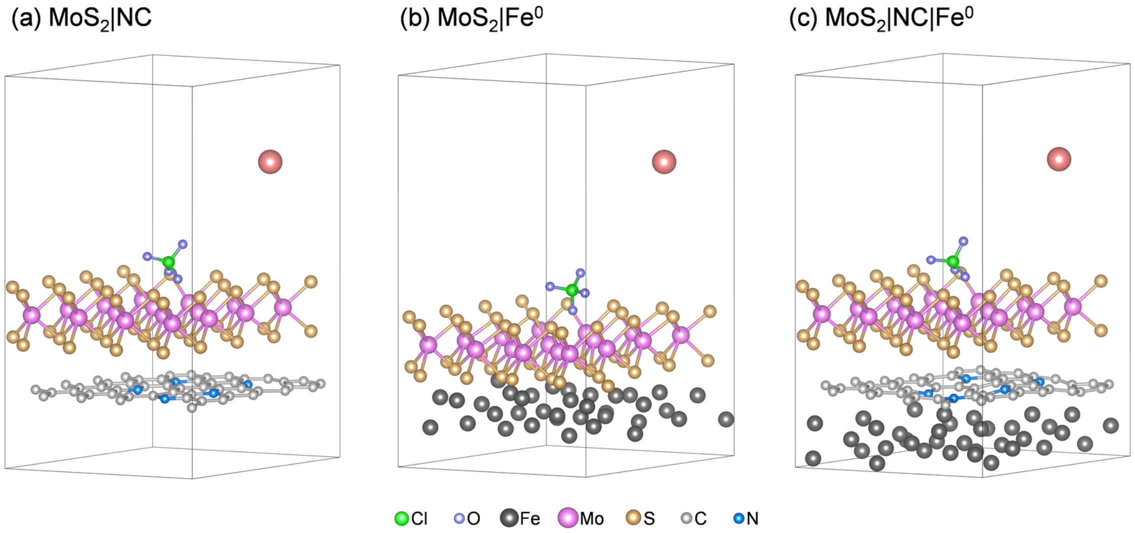

Fig. R16. Optimized adsorption configurations of ClO_4^- on (a) MoS_2|NC, (b) MoS_2|Fe^0, and (c) MoS_2|NC|Fe^0 models. A Na^+ counterion is included in each simulation cell to maintain overall charge neutrality for the anionic ClO_4^- adsorbate under periodic boundary conditions. The Na^+ ion resides in the solvent-accessible region away from the active site and does not form direct coordination with the adsorbed ClO_4^- or Mo sites after relaxation.

We have revised the DFT method section to describe the charge-compensation strategy.

> For anionic adsorbates (e.g., ClO_4^-), a Na^+ counterion was introduced in the cell to maintain charge neutrality. The Na^+ was initially placed in the solvent-accessible region away from the adsorption site and allowed to relax during geometry optimization. In optimized structures, Na^+ did not form direct coordination bonds to the active site and remained solvated (Supplementary Fig. 4). In Fig. 1, the Na^+ was not included for clarity, because it resides far from the surface and may misleadingly suggest the direct involvement in the reaction mechanism. (Lines 116-122 in the revised Supplementary Information)

We have also added structures to the SI (use Fig. R16 as Supplementary Fig. 4). In the main text, in Fig. 1, the Na^+ was not included for clarity, because it resides far from the surface and may misleadingly suggest the direct involvement in the reaction mechanism. In the Supplementary Fig. 4, the position of Na^+ was presented.

References:

(1) Ringe, S.; Hörmann, N. G.; Oberhofer, H.; Reuter, K. Implicit solvation methods for catalysis at electrified interfaces. Chem. Rev. 2022, 122 (12), 10777-10820.

(2) Karimadom, B. R.; Meyerstein, D.; Kornweitz, H. Calculating the adsorption energy of a charged adsorbent in a periodic metallic system – the case of BH_4^- hydrolysis on the Ag(111) surface. Phys. Chem. Chem. Phys. 2021, 23 (45), 25667-25678.

(3) Raju Karimadom, B.; Varshney, S.; Zidki, T.; Meyerstein, D.; Kornweitz, H. DFT study of the BH_4^- hydrolysis on Au(111) surface. Chem. Phys. Chem. 2022, 23 (13), e202200069.

4. Method: "generalized gradient approximation (GGA) with the Perdew-Burke-Ernzerhof (PBE) functional" was used (page 20), but the interface depicted in Supplementary Figure 1 contains non-covalent interactions (surely between MoS2 and CN), which are not included in pure GGA density functionals. Geometry optimizations (finding local minima) should be done using dispersion-corrected DFT (either by semiempirical correction like D3 or using density functionals from the family of nonlocal van der Waals density functionals as optB86b-vdW), see e.g. J. Am. Chem. Soc. 135(16), 6372-6377, 2013. What is the nature of the interaction between MoS2 and CN, or between MoS2 and Fe6?

Thanks for this comment. We would like to clarify that all revised DFT calculations were performed with dispersion corrections. We employed the PBE functional with D3 dispersion correction, as implemented in VASP (IVDW = 12 in the INCAR file). This approach captures long-range van der Waals interactions.

Accordingly, the interfacial geometries shown in Fig. R14 above (Supplementary Fig. 1, updated model structures) were fully optimized using dispersion-corrected DFT, and the weak interactions between MoS_2 and NC are therefore properly described. The interaction between MoS_2 and NC is dominated by van der Waals interactions, rather than covalent bonding, consistent with the absence of Mo−N (or Mo−C) bonds in XPS (Supplementary Fig. 6). We also note that the preliminary Fe6 cluster model has been replaced in the revised work by a periodic Fe(110) slab to represent Fe^0 as a bulk electron donor. Interactions involving Fe^0 at the NC−Fe^0 and MoS_2−Fe^0 interfaces therefore reflect interfacial contact and electron redistribution under dispersion-corrected relaxation.

We have revised the DFT method section (in SI) to state that dispersion corrections were included in all geometry optimizations and energy calculations:

> Long-range dispersion interactions were included using Grimme's DFT-D3 correction (VASP tag IVDW = 12) to account for noncovalent interactions at the MoS_2−NC, MoS_2−Fe^0, and NC−Fe^0 interfaces. (Lines 91-94 in the revised Supplementary Information)

5. In addition, it is stated "Density of states (DOS) analysis shows that MoS2 retains a semiconducting feature, while the NC support introduces additional electronic states near the Fermi level, narrowing the band gap." (Page 5) But the Fermi level (0 eV) crosses both MoS2 and CN states; therefore, it does not make sense.

Thank you for pointing this out. To address this concern, we have re-performed the DOS calculation for MoS_2 slab alone. The updated results (Fig. R17) clearly show that MoS_2 retains its semiconducting character, with the Fermi level located within the band gap and no states crossing it, which is consistent with the expected behavior of semiconducting MoS_2.[1] When combining with NC, additional electronic states appear near the Fermi level in the total DOS. Layer-projected DOS indicates that these near-Fermi states of MoS_2|NC are not solely contributed by NC, but also by MoS_2, which is attributed to the interfacial charge redistribution.

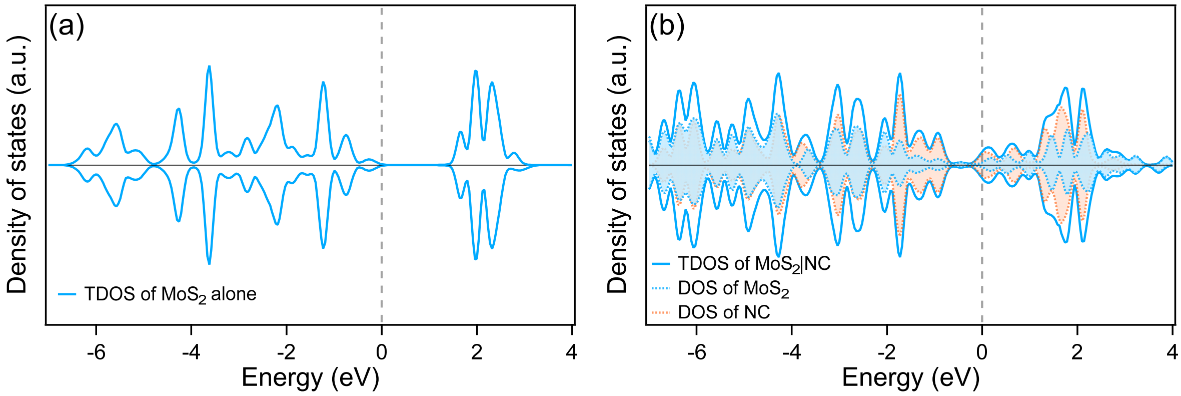

Fig. R17. Density of states (DOS) for (a) MoS_2 alone, (b) MoS_2|NC, with the layer-projected DOS shown for the contributions from MoS_2, and NC.

To clarify the semiconducting feature of MoS_2 DOS analysis, we revised the manuscript, and included the Fig. R17, and other layer-projected DOS in Supplementary Fig. 2.

> Density of states (DOS) analysis for the MoS_2 slab alone confirms its semiconducting feature, with the Fermi level located within the band gap (Supplementary Fig. 2). When combining with NC, electronic states appear near the Fermi level in the total DOS. Layer-projected DOS indicates that these near-Fermi states are not solely contributed by NC, but also by MoS_2, which is attributed to the interfacial charge redistribution. (Lines 109-113 in the revised main text)

References:

(1) Zhao, Y.; Tripathi, M.; Čerņevičs, K.; Avsar, A.; Ji, H. G.; Gonzalez Marin, J. F.; Cheon, C.-Y.; Wang, Z.; Yazyev, O. V.; Kis, A. Electrical spectroscopy of defect states and their hybridization in monolayer MoS_2. Nat. Commun. 2023, 14 (1), 44.

6. Gibbs free energies for all the states were calculated based on the zero-point energy-corrected DFT total energy, which was set as the enthalpy at 0 K" (page 20). No details are provided; does it mean that phonon calculations were performed? If this is enthalpy at 0 K, which pressure was considered?

Thanks for this comment. We did not compute full phonon dispersions, instead, the Gibbs free energies were approximated using zero-point energy (ZPE) and, thermal enthalpy, and entropy corrections obtained from harmonic vibrational frequency analysis. The ZPE-corrected DFT total energy (from static calculations) was used as the enthalpy at 0 K. Regarding pressure, a standard state pressure of 1 atm was assumed, following conventional method.

The Gibbs free energy was estimated as $G(T) = E_{DFT} + E_{ZPE} + \Delta H(T) - TS(T)$, where T = 298K, and E_{ZPE}, ΔH, and S values were obtained from vibrational frequency calculations using VASPKIT.[1]

We have revised the DFT method section to clearly describe it, with other method details in the revised DFT method section in SI.

> Reaction energetics were reported using a free-energy approximation. Gibbs free energies at 298 K (U = 0 V) were calculated as $G(T) = E_{DFT} + E_{ZPE} + \Delta H(T) - TS(T)$. The ZPE-corrected energy was

used as the 0 K enthalpy reference. E_{ZPE}, ΔH, and S values were obtained from vibrational frequency calculations using VASPKIT. (Lines 123-127 in the revised Supplementary Information)

References:

(1) Wang, V.; Xu, N.; Liu, J.-C.; Tang, G.; Geng, W.-T. VASPKIT: A user-friendly interface facilitating high-throughput computing and analysis using VASP code. Comput. Phys. Commun. 2021, 267, 108033.

Response to Reviews of NCOMMS-25-81561A

Donor−acceptor catalysis with defective MoS_2 and Fe^0 breaks barriers to perchlorate reduction under mild conditions

Hejie Qin, Han Yang, Zhenmin Zhang, Yiran Feng, Yuankui Sun, Peng Fan, Zhimin Ao, T. David Waite, and Xiaohong Guan

We appreciate the careful and positive comments from the reviewers. Below, we provide a detailed point-by-point response to each comment. The corresponding revisions have been highlighted in the revised manuscript and are also reproduced below in green text.

Reviewer #2

General Comments:

The authors have carefully revised the manuscript and addressed the main concerns raised in the first round of review. The use of periodic DFT slab models, the clarification of the technoeconomic analysis, and the expanded mechanistic discussion have substantially improved the manuscript. In my view, the main issues raised previously have been satisfactorily resolved, and the conclusions are now adequately supported by the experimental and computational results. Overall, the manuscript is scientifically sound and close to being suitable for publication. I recommend acceptance after minor revision to address the following points.

We sincerely thank you for the positive and encouraging evaluation of our revised manuscript. We are grateful that you find the major concerns raised in the first round have been satisfactorily addressed, and that the revised computational models, clarified technoeconomic analysis, and updated mechanistic discussion have strengthened the manuscript. Following your further suggestions, we have made additional minor revisions to improve the precision, clarity, and readability of the manuscript, as detailed below.

Specific Comments:

1. The disappearance of ClO_4^- should be described as pseudo-first-order kinetics, not first-order kinetics. Please revise this terminology consistently in the main text and Supplementary Information.

Thank you for this suggestion. We have revised this terminology consistently throughout the main text and Supplementary Information, including the Results section and the relevant figure captions:

The disappearance of ClO_4^- followed pseudo-first-order kinetics under all tested conditions. (Lines 229-230 in the main text)

The solid line is the pseudo-first-order fit. **b**,**c**,**d**, Influences of Fe^0 dose (**b**), Mo loading (**c**) and pH (**d**) on the pseudo-first-order rate constants. (Caption of the Fig.2 in the main text).

The solid line is the pseudo-first-order fit. (Captions of the Supplementary Fig. 12, 13, 14, 15, 17, 18, and 23 in the SI).

2. The HRTEM discussion should be slightly refined. Distorted or discontinuous lattice fringes support structural disorder or defective domains, but standard HRTEM does not directly identify individual sulfur vacancies. Please link the vacancy assignment more explicitly to the EPR results and related evidence.

Thanks for this suggestion. We agree that the HRTEM does not directly identify individual sulfur vacancies. Accordingly, we have revised the HRTEM discussion to state that the observed distorted and discontinuous lattice fringes support structural disorder and defective domains in MoS_2, instead of sulfur vacancies.

Furthermore, the HRTEM image also displays a transition between lattice domains, distorted lattice fringes, and discontinuous fringes, marked by red, yellow, and white circles, respectively, suggesting the structural disorder and defective domains the MoS_2 phase. (Lines 171-174 in the main text)

3. The description of the MoS_2–NC interface could be slightly moderated. The absence of detectable Mo–C or Mo–N bonds supports a predominantly noncovalent interface, but the DFT results also indicate interfacial charge redistribution. The wording could therefore be slightly moderated to clarify that the interface is predominantly noncovalent while still involving electronic coupling between the two components.

Thank you. We have moderated the description of the MoS_2–NC interface to clarify that the absence of detectable Mo–C or Mo–N bonds support a predominantly noncovalent interface, while the DFT results indicate interfacial charge redistribution and thus electronic coupling between the two components.

Meanwhile, the DFT results indicate interfacial charge redistribution, implying that this predominantly noncovalent interface still involves electronic coupling between two components. (Lines 192-194 in the main text)

4. The revised manuscript is somewhat dense in places. A light editing pass to reduce minor redundancies and improve readability may help clarify the main storyline, although this is not essential for acceptance.

Thanks for this suggestion. In response, we performed a light editing pass throughout the revised manuscript to reduce minor redundancies and improve readability.

Together, these results confirm the MoS_2 on NC supports contains abundant defects and that the defect density can be tuned by adjusting the Mo loading. (Lines 214-216 in the main text)

Therefore, reduction in this system proceeds through an OAT mechanism, with Fe^0 serves as the direct electron donor. (Lines 298-299 in the main text)

ClO_4^-, present exclusively in the cathode compartment at an initial concentration of 1 mM, achieved 23.6% reduction over 72 h, confirming that the electrons were supplied solely by Fe^0. (Lines 303-305 in the main text)

Reviewer #3

General Comments:

The manuscript "Donor−acceptor catalysis with defective MoS2 and Fe0 breaks barriers to perchlorate reduction under mild conditions" is a joint computational-experimental study, and I was asked to consider the computational part.

I appreciate the authors' efforts in the computational part of the manuscript. It is surprisingly completely recalculated; however, it seems it was necessary. The computational model is now well described, the methods are clear and transparent, and the statements based on calculations are sufficiently supported by the obtained data.

Thank you for the careful evaluation of the computational aspects of this work. We are encouraged that you find the updated computational model to be well described, the methodology clear and transparent, and the calculation-based interpretations to be adequately supported by the obtained results. In response to your additional suggestions, we have made several further minor revisions and clarifications, as detailed below.

Specific Comments:

1. ClO4− configuration on MoS2 changes from the last version. Could the authors confirm they tried more adsorption sites and ClO4− orientations?

Thank you for this question. Yes, we tested several adsorption sites and ClO_4^- orientations on the MoS_2 model with three S vacancies. Specifically, we examined adsorption at different undercoordinated Mo sites as well as different initial orientations of the ClO_4^- molecule relative to the surface.

Across these trials, the outcomes were generally less suitable than the configuration shown in the revised manuscript. In some cases, ClO_4^- interacted only weakly with the sites and relaxed away from them. In other cases, spontaneous Cl−O bond cleavage occurred during structural optimization, suggesting an unrealistically barrierless pathway that was not appropriate for reaction-energy analysis.

The configuration presented in the manuscript was therefore selected because it represents a stable adsorbed state and enables a physically meaningful evaluation of the subsequent reaction steps. We have clarified this point in the revised manuscript/Supplementary Information.

> For ClO_4^- adsorption on Mo sites, we examined several initial configurations, including adsorption at different undercoordinated Mo sites and different initial orientations of the ClO_4^-. The configuration presented in the manuscript was selected because it converged to a stable adsorbed state and enabled a physically meaningful evaluation of the subsequent reaction steps. (Lines 116-120 in the SI)

2. The authors used the PBE-D3 method, which is the Perdew-Burke-Ernzerhof functional with Grimme's D3 dispersion correction, for optimisations compared to PBE (Perdew-Burke-Ernzerhof functional without dispersion correction) in the original versions. What is the nature of the interaction between ClO4− (perchlorate anion) and MoS2 (molybdenum disulfide)? Following

the interaction energies, it should be a non-covalent interaction. But if this is dominating, how is it possible that the interaction energies drastically decreased from the last version (PBE) to the new version (PBE-D3)? (about one half: -0.70 electronvolts (eV) (old) to -0.37 eV (new), MoS2-NC case, Figure 1) . It seems contra-intuitive.

Thank you for this insightful comment. We would like to clarify that the decrease in adsorption energy from the previous version to the revised one should not be attributed to the D3 correction alone, because several methodological changes were introduced together in the revised results, including D3 dispersion correction, implicit solvation, and a Na^+ counterion for charge neutrality.

For an anionic adsorbate such as ClO_4^-, the solvation is an important factor of adsorption energy. In the aqueous phase, free ClO_4^- is stabilized relative to a gas-phase or unsolvated reference state, which reduces the thermodynamic driving force for adsorption and thus leads to less negative adsorption energies. In addition, the Na^+ counterion modifies the electrostatic environment of the periodic cell and provides a more realistic description of the charged adsorption system. Accordingly, the revised adsorption energies are best understood as resulting from the combined methodological updates, which provide a more realistic description of ClO_4^- adsorption under aqueous reaction conditions.

3. The authors stated, "For anionic adsorbates (e.g., ClO4−), a Na+ counterion was introduced in the cell to maintain charge neutrality." This seems fine. But can the authors prove that the actual quantum-mechanical solution of the electronic Schrodinger equation provides the right ions ClO4− and Na+? Another solution, ClO4 and Na (both radicals), is also possible and analysis (e.g., by Bader analysis) is missing.

Thanks. Following your suggestion, we performed Bader charge analysis for the optimized adsorption configuration to verify that the converged electronic structure corresponds to an ionic Na^+/ClO_4^- pair rather than neutral radicals. The calculated Bader charge on Na is +0.9996 e, indicating that Na has fully lost its valence electron. Meanwhile, the total Bader charge of the ClO_4 moiety is −0.9092 e, confirming that the adsorbate remains an anionic perchlorate species. These results support that the converged electronic state is well described as Na^+ and ClO_4^-, rather than a neutral radical pair. Accordingly, the use of Na as a counterion to maintain charge neutrality is electronically justified in our calculations. We have added this clarification to the revised Supplementary Information.

Bader charge analysis further confirmed that the introduced Na counterion exists essentially as Na^+ (+0.9996 e), while the perchlorate moiety retains an overall negative charge (−0.9092 e), supporting the intended ionic description of Na^+ as the counterion and ClO_4^- as the anionic adsorbate in the charge-compensated calculations. (Lines 121-124 in the SI)

4. The model seems fine now, e.g., in Supplementary Figure 1. If bonds in MoS2 and CN are depicted by tubes, could the author use the same for iron? At this moment, the iron layer seems rather as amorphous, but it is declared as a (110) slab.

Thank you for this comment. we have revised Supplementary Figure 1 to depict the Fe layer using tube bonds, consistent with the representation used for MoS_2 and

NC. This revision makes the crystallinity of the Fe(110) slab clearer in the top view. In addition, we found that an incorrect figure had been used for the MoS_2|NC|Fe^0 model (the image of the MoS_2|NC model). This has now been corrected, and the revised Supplementary Figure 1 shows the proper MoS_2|NC|Fe^0 structure.

Reviewer #4

General Comments:

1. Reviewer 1: In the general comments, Reviewer 1 commented that "this work is technically sound, provides a comprehensive dataset combining experimental and computational results, and addresses an environmentally important challenge." However, the reviewer challenged that "the conceptual advance in the catalyst design relative to the authors' previous MoS_2/ZVI study focused on the removal of chlorinated hydrocarbons (Chen et al., 2025, DOI: 10.1021/acs.est.5c00564) appears limited. Furthermore, the technoeconomic analysis relies on unrealistic assumptions regarding nZVI costs, undermining one of the paper's main claims about practical competitiveness." Consequently, Reviewer 1 claimed that "I do not believe the manuscript reaches the level of novelty and impact expected for publication in Nature Communications. However, it could make a valuable contribution to a more specialized journal in environmental catalysis or water treatment.

Authors' responses: About the novelty level and the prior work with MoS2/ZVI (DOI: 10.1021/acs.est.5c00564), the authors provided a thorough justification: 1) the new material has N-doped carbon (NC) added and addressed the unique roles of NC with additional experimental data (especially comparison of the new material with the prior material was quite convincing of the novelty) and computational evidence. 2) The target contaminant perchlorate was indeed different from chlorinate hydrocarbons – the prior material was not working to perchlorate.

For the other major point about the technoeconomic analysis, the authors completely revised the section to provide an order-of-magnitude preliminary estimated and benchmarked with the Pd/H2 system to showcase the advantage.

My comments: The authors provided solid evidence addressing both of the major concerns of Reviewer 1. In addition to the large amount of additional experimental data, the authors completely revised the computational models and added additional characterization data to reveal deeper insights of the material synergy and underlying mechanisms governing the donor-acceptor process.

Overall, the amount of data, the computational evidence, and the depth of the analysis make this paper a strong case and well worthy of publishing in this journal.

The authors spent a great deal of effort in the preliminary cost analysis, as indicated by the other reviewers as well, while such information is important for practical application, such estimates at this level carry lots of uncertainties. As such, benchmarking again some other known technologies should be enough to make a case.

In the subsequent specific comments, Reviewer 1 criticized the claim that "DFT calculations guided the catalyst design is not convincing.", which I agreed, and "The purported enhancement of electronic conductivity by N-doped carbon cannot be substantiated from the calculations." Again, the authors did a fine job in addressing these concerns by updating their models used.

In addition, I would like to suggest the authors also consider the effect of N-C on the surface hydrophobicity – especially considering perchlorate has a much lower hydration energy compared to common inorganic anions such as sulfate and chloride.

Overall, the authors addressed the comments very well. This work contains tremendous amounts of experimental data and insightful analysis, and is worth publishing.

We sincerely thank you for the careful and highly encouraging assessment of our revised manuscript. We greatly appreciate your recognition that the major concerns raised previously regarding the conceptual advance relative to our earlier work and the technoeconomic analysis have been adequately addressed. We are also grateful that you find the additional experimental data, the revised computational models, and the added characterization results to provide deeper insight into the material synergy and the donor−acceptor mechanism, and to make the manuscript suitable for publication in this journal.

We also appreciate your thoughtful suggestion regarding the possible influence of the N-C support on surface hydrophobicity. We agree that this may be a factor that in the behavior of the system and tried to measure it. However, in the present system, we do not have a suitable comparator that would allow the effect of hydrophobicity to be isolated in a meaningful way. For example, unsupported MoS_2 or MoS_2 on a different carbon support differs not only in surface hydrophobicity, but also in other key properties such as defect structure, electron-transfer behavior, etc. As a result, any difference in reactivity could not be unambiguously attributed to hydrophobicity alone. Therefore, although hydrophobicity may contribute to the system behavior, we did not include it as a mechanistically resolved conclusion in the present manuscript.
